# Intrinsic Disorder in Proteins with Pathogenic Repeat Expansions

**DOI:** 10.3390/molecules22122027

**Published:** 2017-11-24

**Authors:** April L. Darling, Vladimir N. Uversky

**Affiliations:** 1 Department of Molecular Medicine, College of Medicine, Byrd Alzheimer’s Institute, University of South Florida, Tampa, FL 33612, USA; 2 James A. Haley Veteran’s Hospital, Tampa, FL 33612, USA; 3 Institute for Biological Instrumentation of the Russian Academy of Sciences, Pushchino, Moscow Region 142290, Russia

**Keywords:** protein repeat expansion, intrinsically disordered protein, intrinsically disordered protein region, homorepeats, protein aggregation, proteinopathies

## Abstract

Intrinsically disordered proteins and proteins with intrinsically disordered regions have been shown to be highly prevalent in disease. Furthermore, disease-causing expansions of the regions containing tandem amino acid repeats often push repetitive proteins towards formation of irreversible aggregates. In fact, in disease-relevant proteins, the increased repeat length often positively correlates with the increased aggregation efficiency and the increased disease severity and penetrance, being negatively correlated with the age of disease onset. The major categories of repeat extensions involved in disease include poly-glutamine and poly-alanine homorepeats, which are often times located in the intrinsically disordered regions, as well as repeats in non-coding regions of genes typically encoding proteins with ordered structures. Repeats in such non-coding regions of genes can be expressed at the mRNA level. Although they can affect the expression levels of encoded proteins, they are not translated as parts of an affected protein and have no effect on its structure. However, in some cases, the repetitive mRNAs can be translated in a non-canonical manner, generating highly repetitive peptides of different length and amino acid composition. The repeat extension-caused aggregation of a repetitive protein may represent a pivotal step for its transformation into a proteotoxic entity that can lead to pathology. The goals of this article are to systematically analyze molecular mechanisms of the proteinopathies caused by the poly-glutamine and poly-alanine homorepeat expansion, as well as by the polypeptides generated as a result of the microsatellite expansions in non-coding gene regions and to examine the related proteins. We also present results of the analysis of the prevalence and functional roles of intrinsic disorder in proteins associated with pathological repeat expansions.

## 1. Introduction

It is clear now that the protein universe includes not only globular, transmembrane, and fibrous proteins but also intrinsically disordered proteins (IDPs) and hybrid proteins with ordered domains and intrinsically disordered protein regions (IDPRs) [1,2]. In fact, there is a growing amount of evidence supporting the idea that many protein regions, and even entire proteins, lack stable tertiary and/or secondary structure in solution, and instead exist as dynamic conformational ensembles of interconverting structures [3,4,5,6,7]. Although these IDPs and IDPRs fail to form specific 3D structures, they are biologically active [2,8,9,10,11,12,13].

Being typically involved in regulation, signaling and control pathways, such as those involved in the cell cycle, IDPs/IDPRs are characterized by specific functionality [14,15] that complement the functional repertoire of ordered proteins, which have evolved mainly to carry out efficient catalytic and transport functions. Some illustrative biological activities of IDPs/IDPs include regulation of cell division, transcription and translation, signal transduction, protein phosphorylation and other posttranslational modifications, storage of small molecules, chaperone action, and regulation of the self-assembly of large multi-protein complexes such as the ribosome [2,9,11,14,15,16,17,18,19,20,21,22,23,24,25]. As a matter of fact, the lack of rigid globular structure under physiological conditions provides IDPs/IDPRs with a remarkable set of unique functional advantages [26,27,28,29,30,31]. For example, large conformational plasticity allows IDPs/IDPRs to be promiscuous binders and interact efficiently with multiple different targets [2,12,32,33]. The functional importance of being disordered has been intensively analyzed and systemized in several dedicated reviews [2,10,18,24,34]. Many IDPs/IDPRs are known to undergo a disorder-to-order transition upon functioning [10,24,34,35]. When IDPRs bind to signaling partners, the free energy required to bring about the disorder to order transition takes away from the interfacial, contact free energy, with the net result that a highly specific interaction can be combined with a low net free energy of association [10,36]. High specificity coupled with low affinity seems to be a useful pair of properties for a signaling interaction so that the signaling interaction is reversible. In addition, a disordered protein can readily bind to multiple partners by changing shape to associate with different targets [32,33]. In addition to decoupled high binding specificity and low affinity, disorder has several clear advantages for functions in signaling, regulation, and control [2,8,10,11,13,17,18,19,20,25,37,38,39].

Many IDPs/IDPRs are involved in the pathogenesis of numerous human diseases (disorders), giving rise to the “disorder in disorders” or D^2^ concept [40]. According to this concept, involvement of IDPs/IDPRs in the development of various diseases is defined by the unique structural and functional properties of these representatives of protein universe. Such diseases originate not only from the misfolding of causative IDPs/IDPRs but also from their misidentification, misregulation, and missignaling. Among human maladies associated with misbehavior of IDPs/IDPRs are various neurodegenerative diseases [41,42,43].

The absence of ordered structure in IDPs/IDPRs proteins has been associated with some specific features of their amino acid sequences, such as the presence of numerous uncompensated charged groups (often negative); i.e., a high net charge at neutral pH, which is a result of the extreme pI values in such proteins and a low content of hydrophobic amino acid residues [9]. In fact, IDPs/IDPRs were shown to be significantly depleted in order-promoting amino acid residues, such as Trp, Tyr, Phe, Ile, Leu, Val, Cys, and Asn, and enriched in disorder-promoting amino acids, such as Ala, Arg, Gly, Gln, Ser, Glu, Lys, and Pro [10,44,45,46]. Furthermore, IDPs/IDPRs are typically characterized by low sequence complexity and might contain numerous sequence repeats [37,47,48,49].

Amino acid tandem repeats are abundant in eukaryotes, where they are found within many physiologically important proteins playing important roles in protein functionality [50,51]. In general, the length of units in protein repeats can be different, with a special type being repeats with the repetitive unit equals to one amino acid residue (homorepeats). Repeats, especially those with poly-glutamine and poly-alanine sequences, are overrepresented in DNA-binding proteins and transcription factors [50], which, compared to the average protein, are engaged in more protein-protein interactions [50,52]. It is likely that proteins utilize homorepeats for non-specific protein-protein interactions or in modulation of specific interactions [53]. However, although homorepeats are engaged in important physiological interactions, they are prone for expansion via strand slippage of encoding DNA, which can lead to increased aggregation propensity of resulting proteins and subsequent disease. It was also pointed out that among various types of the protein repeats, the homorepeats have the highest tendency to aggregate [54].

Curiously, several human diseases are associated with pathological expansions of repeated sequences in specific proteins [55,56]. Most known homorepeat expansions that correlate with disease are found in exons, where translation occurs in a canonical fashion leading to long stretches of peptide homorepeats [55,57]. In most homorepeat disorders, length of the causing homorepeat expansion is correlated with disease severity and penetrance. Genetically, homorepeat disorders are characterized by the lack of conventional Mendelian transmission and exhibit a phenomenon called anticipation, where the following generation is likely to inherit a longer repeat than the previous one, and this results in increased disease severity with earlier onset [58,59,60]. This is because at the genetic level, the expanded repeats are characterized by meiotic or intergenerational instability and change in size when transmitted from parents to offspring [61]. On the other hand, mitotic instability or somatic mosaicism of such homorepeats defines their size variation within the tissues of an affected individual [61].

In rarer cases, the repeat expansions occur in non-coding regions that normally are not translated. However, some expansions have been shown to lead to unique peptide species through non-canonical mechanisms of translation [62]. In fact, these regions, like those found in exons, commonly undergo frame-shifts that lead to the production of several repeat peptide species [62]. This leads to the increased complication that the interaction between different translation products must be considered because biochemical interactions between different species could alter aggregation propensity, depending on the species, and could cause the peptides to undergo structural changes.

The other correlative feature of peptide repeat length is structure, with the longer repeats generally corresponding to increased disorder [63]. These long disordered stretches of repeat peptides have a propensity to aggregate, which contrasts with shorter peptides that remain diffuse throughout the cell. The appearance of large aggregates leads to a cascade of cellular events that causes toxicity and cell death. Cell death from aggregated repeat peptides is emerging as a common feature in many neurological and developmental diseases but the mechanism that causes it is not yet known.

Proteins affected by repeat expansion mutations are listed in Table 1. The goal of this work is to systematically examine proteins associated with proteinopathies caused by the poly-glutamine and poly-alanine homorepeat expansions, as well as by the polypeptides generated as a result of the microsatellite expansions in non-coding gene regions, with the major focus on the roles of intrinsic disorder in proteins associated with pathological repeat expansions. Here, we first discuss molecular mechanisms of various pathologies associated with repeat expansion and represent related proteins. Next, we represent the results of a systematic analysis of the intrinsic disorder status and the presence of disorder-based functional features (such as sites of posttranslational modifications and disorder-based protein binding sites, known as molecular recognition features, MoRFs) in proteins with pathological repeat expansions.

## 2. Polyalanine Repeat Expansions

**Figure 1:**
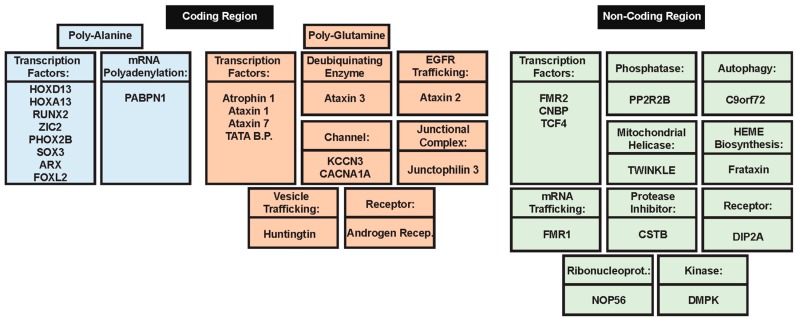
Major known functions of proteins with pathogenic repeat expansions. Proteins with pathogenic expansions have varied functions depending on the type of expansion present. Poly-alanine (poly-Ala) expansions have the least variability in functions, with 8 of the 9 engaging in some sort of transcription regulation. Poly-glutamine (polyQ) expansions that cause pathology have more varied functions, but the majority participate in transcription regulation as well. Pathogenic repeats in non-coding regions occur in genes encoding proteins with the most varied functions. They include everything from catalytic proteins to receptors. Since the repeat extension occurs in the non-coding region of the gene, it is conceivable that there are not more synonymous functions among pathogenic repeat proteins in non-coding regions.

Polyalanine (poly-Ala) tract expansions are linked to 9 inherited human diseases, such as blepharophimosis-ptosis-epicanthus inversus syndrome (BPEIS), cleidocranial dysplasia (CCD), congenital central hypo-ventilation syndrome (CCHS); hand–foot–genital syndrome (HFGS), holoprosencephaly (HPE), oculopharyngeal muscular dystrophy (OPMD), synpolydactyly syndrome (SPD), X-linked mental retardation and abnormal genitalia (XLAG), and X-linked mental retardation and growth hormone deficit (XLMR + GHD) [129]. At the genetic level, the expansion of homopolymeric alanine is caused by the expansion of translated GCN trinucleotide repeats (where N refers to any of the four bases, among which GCG is significantly over-represented in the poly-Ala coding sequences and [130] in the disease-associated genes. It was emphasized that genes coding for poly-Ala stretches longer than four alanines are rather common in human genome that contains 494 such proteins possessing 604 poly-Ala domains [130]. Importantly, as shown in Figure 1, transcription factors are involved in 8 of the 9 poly-Ala expansion diseases and account for 36% of human proteins with poly-Ala tracts [131]. Poly-Ala repeats hang on the cryptic edge of toxicity depending on their length, which is directly correlated with their structure. Unlike other repeat expansions seen in disease, there is a low degree of polymorphism for poly-Ala tract repeats, most likely due to an altered mechanism of expansion as opposed to expansion of other repeats [76]. Poly-Ala tracts are thought to extend by unequal allelic homologous recombination during meiosis, while other disease relevant expansions extend due to DNA polymerase slippage during translation [70]. These observations, in addition to the fact that proteins containing poly-Ala tracts are highly conserved in mammals, makes it conceivable that diminutive extensions to alanine tracts are sufficient to cause cellular dysfunction with subsequent disease pathology [129].

### 2.1. Molecular Mechanisms of Poly-Ala Expansion Diseases

Extension of poly-Ala tracts lead to structural changes in the repeat peptide that may indicate mechanisms of disease pathogenesis. Shorter length peptides are predominantly disordered with short α-helical sections, which, upon expansion, become more prevalent [132]. Increased length tends to push the peptides towards either the degradation or aggregation pathway depending on repeat number, with the longest tending towards aggregation due to length dependent formation of stable beta sheets that are resistant to degradation [68]. In fact, poly-Ala repeat peptides involved in disease have been shown to form large intracellular inclusions of the aggregated peptides. These large inclusions cause cellular dysfunction; however, this is not observed unless their length exceeds 19 repeats [65,76]. Because poly-Ala extensions cause misfolding and subsequent protein aggregation, they are now grouped into a growing class of maladies termed as protein misfolding diseases.

All poly-Ala tract expansions involved in disease function in the nucleus as transcription factors, with one exception. The exception is PABP-2, a polyadenylate-binding protein 2 (or polyadenylate-binding nuclear protein 1) responsible for controlling the length of polyadenylate tails after mRNA processing. Because of the different functions of PABP-2 in contrast to the other 8 genes involved in poly-Ala expansion disorders, the molecular mechanism of disease pathogenesis varies. Extension of the poly-Ala containing transcription factors leads to formation of dense aggregates that mis-localize to the cytoplasm. These aggregates can contain both the mutant extended peptide as well as the wild-type peptide, indicating that the mutant can sequester the normal functioning protein thus producing a dominant effect [77,133,134].

In contrast, expansion of the poly-Ala tract in PABP-2 does not cause protein mis-localization. Instead, PABP-2 remains in the nucleus, where it can perform its normal physiological function. However, only a very small extension to the poly-Ala tract causes the protein to aggregate and appear as large nuclear inclusions. It seems that the inclusions are resistant to degradation once they reach a certain size as evidence by their co-localization with ubiquitin and proteasomal subunits as well as the increase in beta sheet structures [77,133,134]. Although there is some evidence regarding the mechanism by which poly-Ala repeats leads to disease pathogenesis, it is not well understood.

### 2.2. Genes Associated with Poly-Ala Expansion Diseases

Poly-Ala tract expansions in transcription factors are commonly associated with birth defects in humans that cause malformations of the brain, digits, limbs, and heart [64]. For example, homeobox genes involved in the regulation of patterning during limb development can contribute to developmental defects when expansions of their poly-Ala tracts occur above identified thresholds [66]. One such gene is homeobox D13 (*HOXD13*). HOXD13 protein contains a poly-Ala tract in the first exon, which upon expansion above an 8-alanine threshold, is associated with human synpolydactyly (SPD) [64,65,66]. SPD is an autosomal-dominant disease resulting in limb malformations, usually including digit duplication [64,66]. The mechanism by which the expansion contributes to malformation is unclear, but the localization of the protein changes from nuclear to cytoplasmic as a result of poly-Ala tract expansion, with the cytoplasmic protein appearing as amorphous aggregates [64,65,66].

Another HOX gene involved in developmental abnormalities due to a poly-Ala expansion is *HOXA13*. Mutations that cause an additional 6 alanines in the poly-Ala stretch of the corresponding protein have been linked to hand-foot-genital syndrome (HFGS) [67]. HFGS is another dominantly inherited developmental defect that causes malformations in the genitourinary tract and distal limbs [66]. The expansion causes a disruption in HOXA13 protein-protein interactions, but it is not clear exactly how developmental malformations occur [67]. Similar to the manifestations of HOXD13 poly-Ala expansion, HOXA13 expansion also results in mislocalization of the protein to the cytoplasm as well as misfolding when expansion occurs [65].

Homeobox genes represent an additional set of genes that are developmentally important, specifically being highly involved in neural development. Therefore, mutations associated with homeobox genes can lead to devastating neurological defects. Two such genes, *ARX* and *SOX3*, are involved in development of the central nervous system and function as transcription factors containing several poly-Ala stretches [71,72]. Expansion of the poly-Ala stretch in SOX3 protein is associated with X-linked mental retardation with growth hormone deficiency as well as X-linked hypopituitarism [72,74]. The latter is due to two specific poly-Ala expansions, addition of 7 or 11 alanines, with the longer being associated with a more severe disease phenotype [74].

ARX is exceedingly dangerous due to the presence of multiple poly-Ala tracts, potentiating the risk of disease due to expansion. Because ARX has multiple stretches with expansion potential, it is associated with several developmental diseases of varying severity. The most common malady arising from the aberrant poly-Ala expansion is ARX-linked mental retardation, a non-syndromic form of X-linked mental retardation; however, syndromic X-linked mental retardation is also a common outcome [77,133,134]. Other disorders that have been linked to ARX poly-alanine tract expansions include hydrocephaly with abnormal genitalia, myoclonic epilepsy with spasticity and mental retardation, Partington syndrome, and X-linked infantile spasms [77,133,134].

Two additional transcription factors associated with developmental defects due to poly-Ala expansions include RUNX2 and ZIC2. RUNX2 is a bone-specific transcription factor with a poly-alanine tract [77,133,134]. When expansion occurs above a threshold of 10 repeats cleidocranial dysplasia, a bone developmental defect, occurs [68,131]. ZIC2 is a zinc finger transcription repressor that has been extensively linked to holoprosencephaly, a developmental defect where the midline of the brain does not properly form and therefore there is no separation of the brain into hemispheres [73,131]. Similar to other poly-Ala expansion disorders, expansion in these two transcription factors causes protein mislocalization, misfolding, and aggregation [68].

Development and regulation of the autonomic nervous system occur by various transcription factors in addition to homeobox genes. PHOX2B is an example that is involved in autonomic nervous system development [72]. PHOX2B contains a 20-residue C-terminal poly-alanine tract that, upon expansion, can result in congenital central hypoventilation syndrome (CCHS) [72,73]. CCHS is a disorder that effects breathing due to a disruption in autonomic nervous system regulation, which is especially problematic when sleeping [72]. The poly-Ala expansion in PHOX2B results in nuclear localization of the repeat peptide and defects in nuclear import [72]. Unlike in the previously mentioned poly-Ala expansion related disorders, expansion in PHOX2B causes nuclear localization of the protein as opposed to cytoplasmic, offering evidence that not all poly-Ala tract expansions in transcription factors result in similar cellular defects.

Transcription factors in the Forkhead family are known to play important roles in the maintenance of differentiated cells lines, embryogenesis, and tumorigenesis [132]. Therefore, they are commonly involved in human developmental diseases with most of them resulting in aberrant ocular manifestations [78]. One member of this family, FOXL2, is a transcription factor involved in both eye and ovary development [77]. It contains a poly-alanine stretch and upon expansion is correlated with blepharophimosis syndrome (BPES), a developmental disorder that causes defects in the ovary and eyelid [78,135]. The WT protein functions in the nucleus and appears diffuse throughout, but poly-Ala expansions cause FOXL2 to mislocalize to the cytoplasm in aggregated form and diminish its role as a transcription regulator [78].

The only gene associated with disease due to poly-Ala repeat expansions that is not a transcription factor is *PABPN1* encoding PABP-2 protein. This gene contains the shortest poly-alanine expansion associated with disease, with the addition of only 2 alanines sufficient to cause a disease phenotype [65]. Like in most other cases, the repeat length closely correlates with disease penetrance and severity, and extended stretches lead to muscle weakness with accompanying nuclear inclusions that are slow to evolve [77]. Expansions in the poly-alanine tract of PABP-2 have been linked to oculopharyngeal muscular dystrophy (OPMD), which is an adult-onset disorder marked by the presentation of progressive dysphagia, eyelid ptosis, and proximal limb weakness [81]. In addition, the skeletal muscle of those affected contains intranuclear filament inclusions that contain PABP-2 and biopsies show that inclusions are accompanied by myopathic and neurogenic changes [81]. OPMD is the only poly-Ala expansion disorder where onset occurs in adulthood and its presentation, pathology, and onset is more similar to polyglutamine expansion diseases than to other poly-Ala tract expansions [77]. PABP-2 demonstrates the ability of poly-Ala expansions to cause dysfunction even when they are not part of a transcription regulatory protein.

Therefore, while most proteins-carriers of pathogenic poly-Ala expansions behave similarly and engage in analogous functions, there are exceptions. Those exceptions demonstrate the ability for poly-Ala tract extensions to cause pathology in a multitude of ways, depending on the functionality and structure of the translated protein product. The very short extensions needed for poly-Ala tract expansion disease phenotypes to penetrate is directly correlative to structural changes in the resultant translational product. This highlights the fact that the structural transition of the protein containing the extended alanine repeat region may be the defining step that leads to cellular dysfunction and subsequent disease presentation.

## 3. Poly-Glutamine Repeat Expansions

Currently, there are at least twelve known hereditary diseases in which the expansion of a CAG repeat in the gene leads to neurodegeneration [136,137]. Table 1 shows that these poly-glutamine repeat diseases includes Huntington’s disease, Huntington’s disease-like 2, Kennedy disease (also known as spinal and bulbar muscular atrophy, SBMA), dentatorubral-pallidoluysian atrophy (DRPLA), spinocerebellar ataxia type 1 (SCA1), spinocerebellar ataxia type 2 (SCA2), SCA3 (also known as Machado-Joseph disease, MJD), SCA6, SCA7, SCA17, and schizophrenia. Note that although the CAG repeat tract length is somehow correlated with schizophrenia, this is not a pathological repeat expansion and a cause of disease. Similarly, expanded poly-glutamine (polyQ) tracts may occur in the case of the *JPH3* gene too, but the related HDL2 is not a typical polyQ disease. The majority of these diseases are accompanied by the progressive death of neurons, with insoluble, granular, and fibrous deposits being found in the cell nuclei of the affected neurons. The neurotoxicity in these diseases is due to the expansion of the (CAG)_N_-encoded polyQ repeat, which leads to the formation of amyloid fibrils and neuronal death. As a matter of fact, polyQ repeat expansions represent the most well studied group of trinucleotide repeat expansions involved in disease, with the discovery of the link between repeat expansion regions and disease being made when a polyQ expansion in the gene that encodes the androgen receptor was linked to SBMA [90,138]. The CAG trinucleotide repeat is highly unstable and therefore the repeat tract length has a high level of polymorphism across affected individuals as well as across different tissue types. Similar to poly-Ala expansions, most polyQ expansions occur in proteins that share common functions. Most disease-related proteins with polyQ expansions are involved in the regulation of neurogenesis or transcription in a DNA dependent manner [66]. In addition, most proteins with polyQ expansions are engaged in physiologically and functionally important promiscuous binding and interact with multiple partners [139]. PolyQ containing proteins have the potential to cause cellular dysfunction in a variety of ways. However, because of the multitude of functional interactions that most of such homorepeat-containing proteins participate in, their ability to aggregate in particular has several pathological implications.

### 3.1. Molecular Mechanisms of PolyQ Repeat Expansion Diseases

PolyQ expansion diseases are considered protein misfolding diseases that arise by a toxic gain of function mechanism that is not well understood (see Figure 1). The categorization as protein misfolding disease comes from the fact that polyQ expansions are associated with highly stable β-rich amyloid-like protein inclusions [140]. Patients affected by the polyQ-expansion-related diseases present with polyQ-containing intracellular inclusions, which serve as a hallmark of this category of diseases [139]. These inclusions have been identified as both nuclear and cytoplasmic, and in addition to proteins with polyQ repeat expansions, contain ubiquitin, chaperone proteins, proteasome units, and various proteinaceous complexes with which the functional proteins are known to be associated [140,141]. Furthermore, these polyQ-expansion-containing proteins become resistant to degradation once they form these large inclusions.

Proteins with polyQ expanded repeats can cause pathology in several ways. First, expansion of the homorepeat region increases the probability that the polyQ containing protein will interact with itself, thereby forming pathological aggregates and deposits [142]. Additionally, when proteins with polyQ expansions aggregate they sequester other polyQ containing proteins, both of biological and pathological repeat lengths, rendering them unable to perform their biological function [143,144]. Aggregation can also cause the sequestration of non-repeat containing proteins such as molecular chaperones, which can be trapped by aggregates when unable to facilitate proper folding [145]. Since the homorepeat region is often involved in protein-protein interactions, expansion accompanied by an increased propensity to aggregate can alter the binding of biological partners and thus lead to pathology [144,145].

However, while it is simplistic to consider that these aggregates are pathological, there are a few perplexing examples of polyQ expansion diseases that cause neuronal toxicity in the absence of any visible intracellular inclusions [146]. In fact, some studies have shown that large amyloid-like inclusions of polyQ, instead of being cytotoxic, play a protective role in the cell by sequestering misfolded toxic proteins [147]. Therefore, these large aggregates may not be the toxic species, and instead the small soluble β-sheet rich oligomers may be the species responsible for pathology [146].

Mechanistically, the linkage of the CAG repeat expansions to cytotoxicity involves the tendency of longer polyQ sequences, regardless of protein context, to form insoluble aggregates [148,149,150,151,152,153,154,155,156]. Some biophysical properties of a series of simple polyglutamine peptides have been analyzed to gain information on potential mechanisms of cytotoxicity [154]. In this study, the close similarity of the far-UV CD spectra of polyQ peptides with repeat lengths of 5, 15, 28 and 44 residues to each other and to that of a polypeptide with a high degree of random coil structure suggested that the length-dependence of disease is not related to a conformational change in the monomeric states of proteins with expanded polyQ sequences [154]. However, spontaneous formation of amyloid-like fibrils was dramatically accelerated for polyQ peptides with repeat lengths exceeding 37 residues [154].

### 3.2. Genes Associated with PolyQ Expansion Diseases

The human genome contains many genes with CAG repeat stretches that are translated into polyQ repeats involved in neurogenesis, transcription factor regulation, and modulate the binding of transcription factor co-activators [157]. All polyQ containing proteins have not been linked to pathology, but several have successfully been identified in repeat expansion diseases. All polyQ expansion-related diseases are inherited in an autosomal dominant manner except for SBMA, which is X-linked. In addition, expansion of the polyQ tract does not lead to a disease phenotype unless a certain threshold repeat number is reached. Unlike in poly-Ala expansions, the pathogenic repeat length is significantly longer and has a stronger inverse relationship with disease severity, age of onset, and penetrance [61].

The most well studied polyQ expanded gene is HTT, which upon expansion of the homopeptide region, is responsible for the pathogenesis associated with Huntington’s disease. Huntington’s disease is a dominantly inherited motor neuron disease that generally affects middle aged adults but can also present as early-onset and in a juvenile-form if repeat lengths exceeds 70. The decline in motor functioning generally begins with chorea and progresses over an average period of 10–15 years [148]. Huntington’s disease ultimately results in death, most commonly from bulbar dysfunction and its related complications [148].

The gene product from *HTT* is the Huntingtin protein, a highly interactive protein. Huntingtin contains several hydrophobic alpha-helices responsible for the mediation of several protein-protein interactions [86]. Using a yeast two-hybrid system, it was shown that Huntingtin directly interacts with 186 other proteins in its interaction network [87]. Because of the hydrophobic nature of many structural features in Huntingtin and its physiologically important promiscuous binding propensity, long extensions in its N-terminus tend to be poorly tolerated by the cell.

Huntington’s Disease Like-2’s (HDL2) only known genetic link comes from a CAG expansion in the *Junctophilin-3* gene, which is detected in 100% of cases [83,84]. The expansion is inherited in an autosomal dominant manner and can be traced back to Africa [158]. When the expansion exceeds the threshold of 50 repeats, it results in disease [159]. HDL2’s clinical manifestations are very similar to those seen in Huntington’s disease, but motor and cognitive symptoms are more variable between patients [158,159]. The disease generally presents itself with chorea, and its progression results in fatality after an average of 15–20 years, although this is inversely correlated with repeat length [84,87].

Junctophilin-3 is normally expressed in the brain and functions to enable the establishment of a junctional complex established between the cytoplasmic membrane and endoplasmic reticulum membrane [86]. The exact mechanism by which the repeat expansion leads to cell death is not known; however there is a decrease is the expression of Junctophilin-3 when the mutation occurs, suggesting that haploinsufficiency of the gene may be key to driving disease pathogenesis in HDL2. The expression is down-regulated when the expansion occurs due to the sequestration of the wild type version of the gene into aggregates of the mutant protein [83].

*Atrophin 1* (*ATN1*) is a gene coding transcription repressor, that upon expansion of a CAG repeat, leads to dentatorubral and pallidoluysian atrophy (DRPLA). DRPLA is a neurodegenerative disease characterized by epilepsy, cerebral ataxia, dementia, chorea, and myoclonus [160]. Healthy individuals have a repeat length between 7–23 in ATN1, but when the expansion exceeds 48, disease ensues [160]. There is an inverse correlation between repeat size and age of onset and disease progression [160].

The gene that encodes the androgen receptor has been convincingly linked to spinal and bulbar muscular atrophy (SBMA). SBMA is a slowly progressing motorneuron disease possessing X-linked inheritance; therefore, only males are affected. Disease presentation includes muscle weakness and atrophy, gynecomastia, testicular atrophy, reduced fertility, and mild androgen insensitivity [90,161,162]. The X-linked inheritance of SBMA stems from the fact that disease is genetically linked to a CAG expansion in the *AR* gene (encoding the androgen receptor) located on the proximal arm of the X-chromosome. The polyQ expansion is located on the amino-terminus of the androgen receptor and upon elongation causes the translated protein to assume an altered structure going from an unfolded state to a stable beta sheet structure [90,161,162]. The change in the structure of the protein is believed to be in favor of the rate limiting structure of aggregation, a soluble oligomer capable of seeding additional aggregation reactions [162]. In fact, histological staining has revealed large insoluble fibers present in the nucleus of SBMA patients [163]. In the human androgen receptor, there are three polyglutamine repeats ranging in size from 5 to 22 residues, stretches of seven prolines and five alanines, and a polyglycine repeat of 24 residues. Polymorphisms of the largest polyglutamine and the polyglycine repeats of this protein were found in a number of human diseases, such as prostate cancer, benign prostatic hyperplasia, male infertility, and rheumatoid arthritis [164].

The seven remaining genes that undergo disease causing expansion of their polyQ regions are all involved in spinocereberal ataxia (SCA), but different genes lead to different forms of SCA. SCA is a dominantly inherited disorder with the primary feature being ataxia, which involves problems with balance, speech, and eye movements. There have been 40 characterized SCAs which differ in age of onset and disease presentation, and so far, 28 have been genetically linked [165].

SCA1, 2, 3, and 17 are all linked to expansions in the coding regions of related *ATXN* genes. SCA1, 3, and 17 are all associated with *ATXN* genes that specifically encode for a nuclear version of the ataxin protein, while SCA2 is associated with a cytoplasmic ataxin protein [140]. The cytoplasmic protein is referred to as ataxin 2 and causes a version of ataxia that resembles Parkinson’s disease but has associated eye degeneration as well [93]. SCA1 is specifically caused by a CAG expansion in *ATXN1* [166]. It causes peripheral neuropathy and hypometric cascades [97,167]. CAG expansions in *ATXN3* lead to SCA3, which is characterized by peripheral neuropathy and ophthalmic change [97]. Expansions in the CAG repeat region in *ATXN7* cause SCA7. SCA7 is mainly a degenerative eye disorder characterized by retinal degeneration with associated visual loss [167]. All three genes involved in the above listed in the nuclear ataxin associated SCAs are involved in functions where DNA binding is necessary, and upon expansion their localization changes from nuclear to cytoplasmic, meaning the protein is no longer able to perform its wild type functions.

Another nuclear protein involved in spinocereberal ataxia that undergoes mis-localization to the cytoplasm upon expansion is the TATA-box-binding protein, TBP [97]. This protein is encoded by the *TBP* gene, in which expansion of either CAG or CAA repeat regions (both codes for the polyQ tracts in the corresponding protein) leads to SCA17 [141]. SCA17 is an ataxia with symptoms ranging from involuntary movements and dementia to psychosis [97]. It can present in children in their first two years and lead to developmental delays and an early death [163].

Not only are DNA-binding proteins involved in ataxia upon extension of CAG repeats, but certain phosphatases and channel proteins are also engaged. For example, CAG repeat expansions in the gene *CACNA1A* that encodes for the P/Q voltage-dependent calcium channel can cause SCA6 [168]. These expansions are generally very small and lead to a very slowly progressing disease that is presented as pure ataxia and occurs for the lifetime of the patient [157,168]. Similarly, CAG long repeat expansions in the *KCNN3* gene that affect the N-terminal region of the small conductance calcium-activated potassium channel KCNN3 (also known as hKCa3 or SK3) might be related to the pathology of schizophrenia and bipolar disorder [169,170,171,172]. Curiously, it was also reported that the polymorphism of schizophrenia symptom can be associated with both the CAG repeat numbers and the difference in allele sizes [82,170].

One of the general trends found in many polyQ extension-related diseases is a noticeable correlation between the number of CAG repeats and the probability of disease onset. For example, in the 3142-residue-long huntingtin, polyQ repeat encoded by the CAG repeat expansion of the exon 1 varies between 16 and 37 residues in healthy individuals, whereas patients with Huntington’s disease have repeats of >38 glutamine residues [173]. Similarly, the age of onset and the severity of the progression of SCA1 are both directly linked to the length of the polyQ tract in ataxin-1 [174,175,176], with the length of the polyQ tract exceeding a threshold of 39–44 glutamine residues being associated with the formation of granular or fibrillar intranuclear aggregates of ataxin-1 and eventual cell death [177,178]. In SBMA, which is associated with the polyQ expansion of the androgen receptor, healthy individuals have a polyQ segment of 15 to 31 residues, whereas the SBMA afflicted individuals have 40–62 glutamine residues [179]. Finally, the age of onset of the DRPLA is inversely correlated with the length of the polyQ track repeat size in atrophin-1, which varies from 7–23 in normal individuals and is expanded to 49–75 in DRPLA patients [88].

PolyQ-related diseases have received the most attention among any repeat expansion disease due to a higher prevalence in the population. These diseases, while considered protein misfolding diseases, have many possible mechanisms that lead to cell pathology. However, there is a common theme of loss of function of the wild type protein in which the extension occurs. Because of the complex nature of the possibly toxicity mechanisms created by polyQ extensions, much more work needs to be done to understand their role in disease to develop effective therapies for those suffering with disease.

## 4. Non-Coding Region Repeat Expansion

Microsatellites are tandem arrays of short (usually <10 bp) units commonly found in eukaryotic genomes [180]. Microsatellite expansions in non-coding regions of genes are the most peculiar type of genetic alteration, since despite the lack of start codons, in some cases, non-canonical translation still ensues generating polypeptides with highly repeated sequences. This aberrant translation complicates the cellular mechanisms of pathology by throwing further insults onto an already injured cell. Generally, these polypeptides are involved in a gain of function toxicity, and their translation is often correlated with promoter methylation of the gene they are located on, rendering it inactive. Therefore, expansions in the noncoding region of genes are especially dangerous and lead to several developmental and neurological diseases.

### 4.1. Molecular Mechanisms of Diseases Associated with Non-Coding Region Repeat Expansions

The mechanisms of pathology for non-coding repeat expansions are not as straight forward as expansions in coding regions since non-canonical translational processes often occur, and there is still little understood about these processes themselves. In addition, not all non-coding expansions have identified repeat peptide products associated with DNA expansions, so while it is plausible that aberrant peptides may arise in all cases, this has not been confirmed. However, for a few expansions, such as those in the 5′ UTR of C9orf72, there is confirmation of peptide translation due to expansions in non-coding regions [108,109,181]. These aberrant peptide products may contribute to cell death through a gain of function toxicity.

In addition, expansions in non-coding regions of DNA lead to the transcription of long mRNA transcripts known to form stable structures that are toxic to the cell. The mRNA itself can cause damage, but the main toxicity is due to its sequestration of RNA binding proteins [55]. Once the RNA transcript has successfully sequestered the RNA binding protein, it forms nuclear foci in cells that are visible using fluorescent histology. In addition, expansions in non-coding regions often lead to loss of expression of the translated protein, which causes a loss of function toxicity [55]. It is not clear whether one of these mechanisms is suffice to cause some expansion diseases or if multiple mechanisms ensue that add insult to injury and lead to cell death [55].

### 4.2. Genes Associated with Non-Coding Region Repeat Expansions

Many of the genes involved in disease that undergo expansions in non-coding regions contain what are known as fragile sites. These fragile sites are specific loci on chromosomes which, following partial inhibition of DNA synthesis, during metaphase, appear as visible gaps [55]. In addition, the sites are associated with the activation of the DNA damage response at stalled replication forks and are considered to create a high level of genome instability [55]. Fragile sites can be classified into two main categories depending on the frequency with which they occur in the population. They can be classified as either common fragile sites or rare fragile sites. Rare fragile sites occur in less than 5% of the population and have increased incidents of breakage that are generally associated with expansion of nucleotide repeats [55]. Rare fragile sites are then further classified by the conditions by which they are induced when in cell culture, with folate-sensitive fragile sites representing the largest group. These folate-sensitive fragile sites have a high prevalence of being located on the X-chromosome and upon expansions of nucleotide repeats, are involved in several diseases.

Fragile X Mental Retardation (FXMR), the most prevalent form of mental retardation in males, has been genetically linked to a fragile site on the X-chromosome known as FRAXA [182]. FXMR causes intellectual disabilities stemming from defects in cognitive development and learning [183]. It is generally more severe in males due to the X-linked inheritance pattern and can cause the characteristic appearance of a long face and prominent forehead and ears in those severely affected [183]. Individuals affected with this disorder also tend to engage in behavioral abnormalities such as hyperactivity, especially at adolescent age, and commonly mimic symptoms that appear in autism [182].

The FRAXA site implicated in FXMR is located on *FMR1* [183,184,185]. *FMR1* encodes an RNA binding protein that has both a nuclear export and import signal, implicating that it may have some role in the nuclear transport of mRNA [184]. Upon an expansion of CGG in FRAXA, the promoter region of *FMR1* is methylated leading to the loss of gene expression. The loss of expression of *FMR1* is the driving factor for disease because the expansion in the absence of downregulation does not lead to a disease phenotype [186].

Although methylation of the promoter region in *FMR1* is required for pathology, the phenotype produced from the CGG expansion is dependent on length and comes in several forms. The first form is present in healthy individuals and contains 6–40 repeats, followed by the intermediate form which has 41–60 repeats, both of which do not lead to any disease phenotypes [182,183]. When the expansion is extended to 61–200 repeats, it is called a pre-mutation and is involved in less severe diseases than FXMR [183]. The permutation is involved in Fragile X-associated tremor/ataxia syndrome, which is a late onset disease characterized by motor degeneration and FMR1-related primary ovarian insufficiency [183,187,188,189].

FRAXE is another rare folate-sensitive fragile site located on the *FMR2* gene of the X-chromosome and is also associated with mental retardation [106,107,110]. The *FMR2* transcript is expressed in placenta and adult brains and is found in high levels in the fetal brain [106]. The gene is translated into a 1311-amino acid protein that is nuclear localized and possess putative transcription transactivation potential [106]. Like in *FMR1* expansions, expansion in FRAXE in *FMR2* is not sufficient to causes disease and must also accompany methylation of the promoter region of the gene and down-regulation [186]. The degree of the amplification of the GCC region is also classified in *FMR2* as either normal, permutation, or full mutation with only the full mutation leading to disease [107]. The mental retardation associated with this expansion is similar to FXMR except it is generally much milder [106].

The last rare folate-sensitive fragile site that undergoes disease causing CGG nucleotide expansions and subsequent methylation is referred to as FRA12A [104]. It is located on the 5’ UTR region of *DIP2b*, which encodes a protein involved in DNA methylation [104]. Upon expansion of CGG, methylation of the promoter region of DIP2b occurs leading to loss of expression that results in a disease phenotype [104]. The expansion induced silencing of *DIP2b* leads to mental retardation that is milder than that seen in FXMR.

The fragile nature of the expansion sites in non-coding regions is becoming more and more evident. Recently, it was found that another expansion involved in disease located at a locus on the *FXN* gene for frataxin displays chromosomal fragility [190]. Expansions of GAA occur in the first intron of *FXN* at an Alu repeat region and lead to a reduction of GAA in the transcribed product due to the impediment of elongation during transcription [110]. This phenomenon can be exacerbated upon an increase in the repeat tract length [110]. Long expansions in frataxin lead to the most common form of ataxia, Frederich’s Ataxia (FRDA). FRDA is an autosomal recessive disease characterized by degeneration in the central and peripheral nervous system as well as the heart [111]. It is one of the more severe forms of ataxia and generally reduces mobility and causes early death most commonly through cardiac complications [111].

One of the most recently discovered repeat expansions involved in disease is a G_4_C_2_ hexanucleotide repeat expansion (HRE) found on the 5’ untranslated region of *C9orf72* [108,109,181]. This expansion is the major genetic cause of Amyotrophic Lateral Sclerosis (ALS) and Frontotemporal Dementia (FTD). ALS is a motor neuron disease characterized by degeneration of upper and lower motor neurons that leads to paralysis and eventually death. FTD is a neurodegenerative disorder where neuron death causes atrophy of the frontal lobe in the brain and leads to a loss in executive functioning and changes in behavior and personality. Since the discovery of the genetic link, rapid progress has been made to identify the molecular mechanism involved in disease.

The *C9orf72* (C9) HRE leads to the partial loss in functioning of the C9 protein, a multifunctional homologue of DENN proteins [191]. DENN proteins function as guanine nucleotide exchange factors for small GTPases [192]. Due to the similarity in structure of DENN proteins to C9, it is predicted that C9 engages in similar functioning, specifically acting as a guanine nucleotide exchange factor for RAB [191,192]. C9 has also been found to play a functional role in endosomal trafficking and autophagy in neurons [191,192]. HRE expansions in C9 lead to decreased expression of the translational product which, in zebrafish, leads to age dependent motor deficits [193]. In addition, the expansions are transcribed into long stretches of mRNA which form nuclear foci in cells that cause toxicity through the sequestration of RNA binding proteins [57].

The last known mechanism by which C9 expansions may lead to pathology is through a gain of function toxicity mechanism. The gain of function comes from the non-canonical repeat associated non-ATG (RAN) translation products of the C9 expansion [194,195]. These products contain tandem peptide repeats and are termed dipeptide repeat (DPR) proteins. The C9 expansion results in six DPRs, one from each of the three reading frames of the sense mRNA (poly-GA/GP/GR) and one from each of the antisense (poly-PR/PG/PA). Note that although C9 expansions are translated into the six reading frames and although all these reading frames are utilized in protein biosynthesis, only five different DPRs are synthesized. This is because at the protein level, it is impossible to discriminate poly-GP and poly-PG generated from the sense and antisense mRNAs. The sense and antisense peptides are sometimes translated in the same cell and have been shown to cause toxicity though various mechanisms such as blocking nuclear transport and impairing the assembly of membrane-less organelles [196,197,198,199,200]. These aberrant peptides are newly defined products of intronic repeat expansions and offer evidence into the possibility of the translation of non-coding DNA upon expansion of repeat regions. Therefore, further studies should be performed to try to identify similar peptide products in non-coding microsatellite expansions that are implicated in disease.

Like in C9, many of the disease-causing expansions in non-coding regions lead to neuro and muscular degenerations. For example, myotonic dystrophy (DM), the most common form of muscular dystrophy that occurs in adults, is linked to DNA repeat expansions [112]. The symptoms of disease include myotonia, muscular dystrophy, cataracts, diabetes, and cardiac conduction defects [201]. DM comes in several forms, and the forms DM1 and DM2 are linked to regions of repeat extensions. DM1 and DM2 are distinguishable by the fact that DM2 is generally a later onset disease and is not present from birth like DM1 [201]. DM1 is associated with CTG expansions on the 3’ end of the gene DMPK, while DM2 is genetically linked to CCTG expansions on the first intron of the gene ZNF9 [112,201]. Both diseases are inherited in a dominant manner and present with multisystemic clinical features [201].

Other form of spinocerebellar ataxias (SCA) are associated with repeats in non-coding regions as opposed to the majority which are linked to polyQ expansions. These include SCA8, 10 and 36, which are dominantly inherited and characterized by seizures, cerebellar ataxia, and anticipation [115,116,202]. SCA36 can be further classified by distinct tongue atrophy and motor neuron degeneration [202], while SCA8 commonly has oculomotor incoordination as a main symptom [123]. SCA8 was the first form of SCA that was linked to a non-CAG repeat expansion [123]. It is genetically linked to a CTG expansion of the *ATXN8OS* gene [122,123] and a complementary CAG repeat expansion in the *ATXN8* gene [124]. In fact, SCA8 is caused by the bidirectional transcription at the SCA8 locus containing *ATXN8OS (ataxin 8 opposite strand)* and *ATXN8* genes and is therefore considered as the ′CTG*CAG′ repeat expansion disease, referring to the complementary expanded base pairs of the *ATXN8OS* (CTG) and *ATXN8* genes (CAG) [124]. Although ataxin-8 protein encoded by the *ATXN8* gene represents a polyglutamine protein, SCA8 is not considered as typical polyQ disease. SCA10 is linked to an ATTCT expansion in the ninth intron of ataxin 10 [115]. The expansion on SCA10 does not have to be continuous to cause disease. In fact, it was shown that several repeat interruptions of varying lengths and sequences can be present in an individual expressing the disease phenotype [115]. SCA36 is linked to a GGCCTG hexanucleotide expansion on nop56 [202]. The disease causing repeat expansion seen on nop56 mRNA exceeds 1500 repeats and is longer than that seen in any other neuromuscular disorder associated with repeat expansions and shows the most dramatic shift from the wild type number of 3–8 repeats to the disease-causing expansion [202].

Progressive myoclonus epilepsy (EPM1) is a mitochondrial myopathy, meaning that the cell pathology is produced from the mitochondria and without sufficient energy production, high energy consuming tissue is compromised. EPM1 is inherited as an autosomal recessive disorder and is characterized by severe, stimulus-sensitive myoclonus and tonic-clonic seizures and myoclonus that is stimulus-sensitive [203]. The disease has been genetically linked to an extension of a repeat region in the promoter region of the cystatin B gene [125]. Cystatin B, without an extension mutation, generally only contains two copies of the dodecamer repeat CCCCGCCCCGCG, but extension beyond 14 repeats leads to EPM1 [125]. However, unlike in most repeat expansion diseases, the length of extension is not correlated with age of onset or severity of disease most likely due to the fact that it occurs in the promoter region [204]. This also suggests that once the repeat is beyond a critical threshold, gene expression is reduced and disease ensues at the same rate regardless of the expansion size [125].

Another repeat expansion disease linked to the non-coding region is involved in ocular degeneration, specifically Fuchs’ endothelial corneal dystrophy (FECD). FECD is an inherited degenerative disease that affects the corneal endothelium which helps to maintain corneal clarity [117]. It generally is an asymptomatic disease in the early stages but later stages present with corneal edema, associated eye pain, and vision loss [117]. FECD has been genetically linked to an intronic CTG expansion in a transcription factor, namely, TCF4 [117]. Cells containing the repeat expansion contain RNA foci and have reduced expression of TCF4, both of which may contribute to cellular pathogenesis.

CAG repeat expansions in the gene *PPP2R2B*, which encodes for protein phosphatase 2, has been genetically linked to spinocerebellar ataxia type 12 (SCA12) [205]. Although CAG repeat expansion mutations are located in exon 7 of *PPP2R2B*, there is no evidence that this CAG expansion results in polyQ production [206]. In fact, it was emphasized that the CAG expansion in *PPP2R2B* has a promoter function [206], and it was also mentioned that this expansion occurs in a 5′-untranslated region of the of *PPP2R2B* gene [102]. CAG repeats numbers 7–28 in normal individuals and 55–78 in SCA12 patients [206]. SCA12 is an ataxia that most closely resembles Parkinson’s disease with symptoms such as loss of movement, tremors, and dementia [90] and which is relatively rare worldwide [207].

Repeat expansion mutations found in non-coding regions are the most variable due to the diversity in the functionality of genes that involved in disease. However, like in the other expansion mutation categories, non-coding expansions still share some common traits with a few exceptions. Most of the non-coding region repeats have an inverse correlation between repeat size and disease severity and penetrance, with the exception of the cystatin B extension. Also, like the polyQ and poly-Ala repeats, most associated diseases are neurodegenerative or neuromuscular in nature. The exception to this is the TCF4 extension, which is associated with opthamalic problems; however these are generally closely associated with neurodegenerative disease presentation.

## 5. Intrinsic Disorder in Proteins Associated with Pathological Repeat Expansions

**Figure 2:**
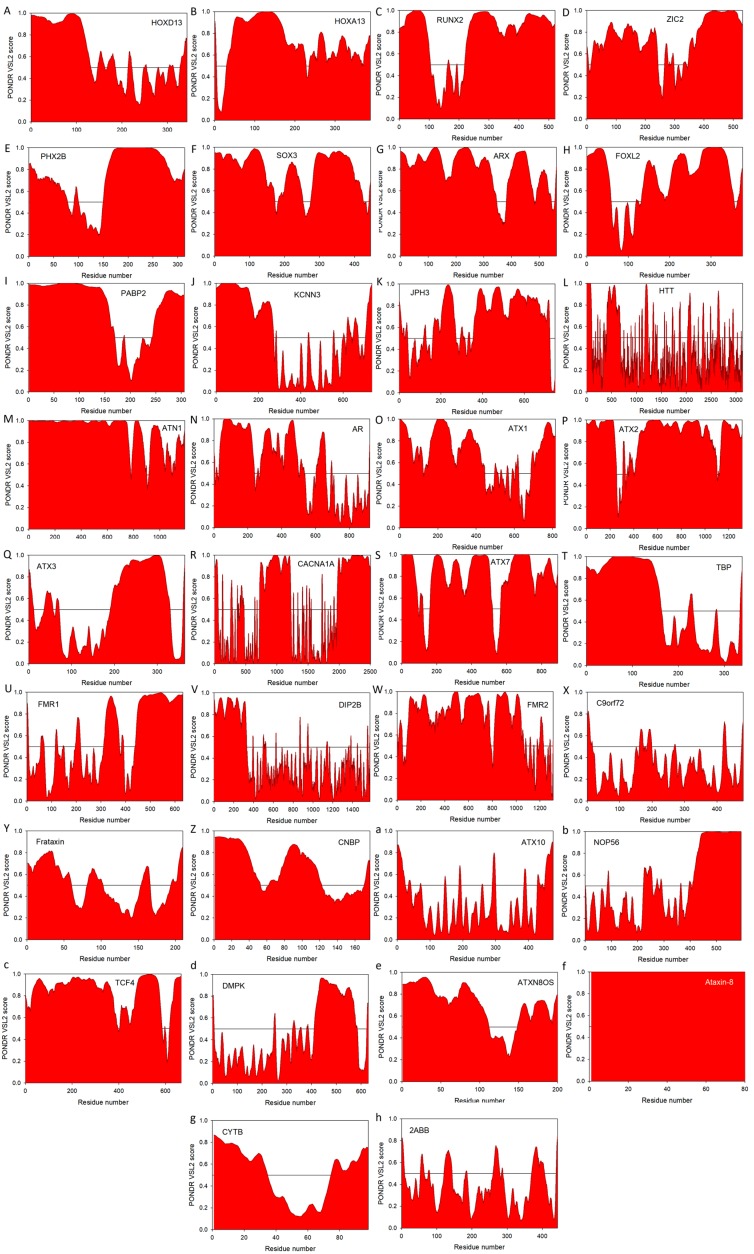
Evaluation of intrinsic disorder propensities of 33 proteins associated with the proteins caused by the nucleotide expansions. Intrinsic disorder predisposition was evaluated by PONDR^®^ VSL2 predictor, which is one of the more accurate stand-alone tools for prediction of the intrinsic disorder status of a target protein. This tool is known to be statistically better for proteins containing both ordered and disordered regions [208,209]. (**A**) Homeobox protein HOXD13 (UniProt ID: P35453); (**B**) Homeobox protein HOXA13 (UniProt ID: P31271); (**C**) runt-related transcription factor 2, RUNX2 (UniProt ID: Q13950); (**D**) Zinc finger protein ZIC2 (UniProt ID: O95409); (**E**) Paired mesoderm homeobox protein 2B (UniProt ID: Q99453); (**F**) Transcription factor SOX3 (UniProt ID: P41225); (**G**) Homeobox protein ARX (UniProt ID: Q96QS3); (**H**) Human FOXL2 (UniProt ID: P58012); (**I**) PABP2/PABPN1 (UniProt ID: Q86U42); (**J**) Small conductance calcium-activated potassium channel protein 3 (SK3, UniProt ID: Q9UGI6); (**K**) Human junctophilin-3 (JP-3, UniProt ID: Q8WXH2); (**L**) Human huntingtin (UniProt ID: P42858); (**M**) Atrophin-1 (UniProt ID: P54259); (**N**) Human androgen receptor (AR, UniProt ID: P10275); (**O**) Ataxin-1 (UniProt ID: P54253); (**P**) Ataxin-2 (UniProt ID: Q99700); (**Q**) Human ataxin-3 (UniProt ID: P54252); (**R**) Voltage-dependent P/Q-type calcium channel subunit α1A (CACNA1A, UniProt ID: O00555); (**S**) Ataxin-7 (UniProt ID: O15265); (**T**) TATA-box-binding protein (TBP, UniProt ID: P20226); (**U**) Synaptic functional regulator FMR1 (UniProt ID: Q06787); (**V**) Disco-interacting protein 2 homolog B (UniProt ID: Q9P265); (**W**) AF4/FMR2 family member 2 (UniProt ID: P51816); (**X**) C9orf72 (UniProt ID: Q96LT7); (**Y**) Frataxin (UniProt ID: Q16595); (**Z**) Cellular nucleic acid-binding protein (CNBP, UniProt ID: P62633); (**a**) Ataxin-10 (UniProt ID: Q9UBB4); (**b**) Nucleolar protein 56 (UniProt ID: O00567); (**c**) Transcription factor 4 (UniProt ID: P15884); (**d**) Myotonin-protein kinase (UniProt ID: Q09013); (**e**) Ataxin-8 (UniProt ID: Q156A1); (**f**) ATXN8OS protein (UniProt ID: P0DMR3); (**g**) Cystatin-B (UniProt ID: P04080); (**h**) Serine/threonine-protein phosphatase 2A 55 kDa regulatory subunit B β isoform (PPP2R2B, UniProt ID: Q00005). In this analysis, scores above 0.5 correspond to intrinsic disorder.

Another important feature linking various diseases associated with the pathogenic repeat expansions is the presence of noticeable disorder in the corresponding carrier proteins, even before the introduction of repeat expansion mutations. This observation is illustrated by Figure 2 that represents the PONDR^®^ VSL2 predictor, which is one of the more accurate stand-alone tools for evaluating intrinsic disorder status in a target protein, being statistically better for proteins containing both ordered and disordered regions [208,209]. Additional information on the intrinsic disorder status and on the presence of disorder-based functional features (such as sites of posttranslational modifications and disorder-based protein binding sites, known as molecular recognition features, MoRFs) for the majority of proteins considered in this review is presented in Supplementary Materials (see Figure S1) as outputs of the D^2^P^2^ database (http://d2p2.pro/) [210] that provides disorder evaluations by several computational tools (such as IUPred [211], PONDR^®^ VLXT [212], PrDOS [213], PONDR^®^ VSL2B [208,209], PV2 [210], and ESpritz [214]). These D^2^P^2^ outputs of multiple disorder predictors are complemented with some important disorder-related functional information (such as location of various curated PTMs and ANCHOR-predicted disorder-based protein-protein interaction sites [215,216], known as molecular recognition features, MoRFs, see [35,217,218,219]). Therefore, for each protein, D^2^P^2^ represents location of IDPRs predicted by various computational tools (shown by differently colored bars). Next, positions of known and predicted functional domains are indicated. This is followed by a blue-green-white bar in the middle of the plot that shows the predicted disorder agreement between nine predictors, with blue and green parts corresponding to disordered regions by consensus. Yellow bars show locations of the predicted disorder-based binding sites (molecular recognition features, MoRFs), whereas differently colored circles at the bottom of the plot show location of various PTMs. Note that D^2^P^2^ information is not available for KCNN3 and CACNA1A, both associated with the polyQ expansion diseases. We also utilized the outputs of the MobiDB database (http://mobid.bio.unipd.it/) [127,128], for further characterization of the disorder status of query proteins. This tool was used because MobiDB generates consensus disorder scores by aggregating the output from ten predictors, such as two versions of IUPred [211], two versions of ESpritz [214], two versions of DisEMBL [220], JRONN [221], PONDR^®^ VSL2B [208,209,222], and GlobPlot [223]. MobiDB also has manually curated annotations related to protein function and structure derived from UniProt [224] and DisProt [225], as well as from Pfam [226] and PDB [227]. Sections below provide a brief overview of the disorder status of all 33 proteins listed in Table 1.

### 5.1. Disorder in Proteins Associated with Poly-Ala Expansion Diseases

Since the majority of proteins related to the poly-Ala expansion diseases are transcription factors, it is not surprising to find that they are expected to be highly disordered proteins. In fact, this is in agreement with the known notion that eukaryotic transcription factors and other proteins involved in the transcription regulation are, in general, highly disordered [228,229,230,231,232]. Furthermore, in addition to be associated with various poly-Ala tract extension-related diseases, many of these proteins are related to the pathogenesis of different cancers, clearly indicating important roles of these proteins in regulation of a multitude of diverse biological processes, which is another important functional characteristic of IDPs.

Figure 2A and Figure S1A show that homeobox protein HOXD13 (UniProt ID: P35453) is a highly disordered protein, with the MobiDB-based predicted consensus disorder content of 33.82%. This protein is associated not only with the poly-Ala tract expansion-based human synpolydactyly (SPD) [233], but deregulation of HOXD13 expression has been detected in breast cancer, melanoma, cervical cancer, astrocytomas, and, more recently, neoplastic tissue samples from 79 different tumor categories, being especially prominent in pancreatic cancer [234,235].

High disorder status of the homeobox protein HOXA13 (UniProt ID: P31271) is illustrated by Figure 2B and Figure S1B and is supported by MobiDB, which indicated that HOXA13 is another highly disordered protein with the consensus disorder content of 34.28%. Similar to HOXD13, HOXA13 might be related to the pathogenesis of both poly-Ala tract expansion-related hand-foot-genital syndrome (HFGS) [67,236] and some types of cancer, such as thyroid [237] and gastric cancers [238] characterized by the aberrant expression of HOXA13, both at gene and protein levels. Both HOXD13 and HOAD13 transcription factors were shown to form DNA-binding trimeric complexes with the TALE superclass proteins MEIS1A and MEIS1B [239]. Curiously, it was shown that multiple peptides derived from HOXD13 and HOAD13 can efficiently interact with MEIS proteins [239], suggesting the presence of complex HOXD13-MEIS and HOAD13-MEIS interfaces, which potentially originated as a result of folding-upon-binding reaction [33,240,241,242].

According to Figure 2C and Figure S1C, as well as based on the MobiDB analysis that showed the disordered residue content of 62.96%, it is clear that runt-related transcription factor 2, RUNX2 (UniProt ID: Q13950), is the most disordered protein with the pathogenic poly-Ala expansion. In addition to the poly-Ala tract (residues 73–89) this protein has a polyQ tract (residues 49–71) and a Pro/Ser/Thr-rich domain (residues 237–521). D^2^P^2^ shows also that RUNX2 has a multitude of disorder-based binding sites (see Figure S1C), some of which coincide with the binding regions known to be involved in interaction with FOXO1, KAT6A, and KAT6B (residues 242–258, 336–439, and 374–468, respectively). Importantly, it was also shown that not only poly-Ala expansion, but also deletion within the poly-Ala tract (reducing its length from 17 to 11 alanines, the 11A allele) of RUNX2 might be pathogenic, being able to significantly enhance fracture risk in post-menopausal females in a site-selective manner related to intramembranous bone ossification [243].

Zinc finger protein ZIC2 (UniProt ID: O95409) has a MobiDB-defined disorder content of 54.89%. Figure 2D and Figure S1D illustrate that this protein has long IDPRs located in its N- and C-terminal tails. The protein is predicted to have a multitude of functional domains, possess several disorder-based binding regions, and have several PTM sites (see Figure S1D). Zic2 has multiple regions with compositional biases, such as a poly-Gly region (residues 490–508), two poly-His regions (residues 20–23 and 231–239), and four poly-Ala regions (residues 25–33, 89–97, 226–230, and 456–470). It has multiple zinc-finger domains (C2H2-type 1, 2, 3, 4, and 5, residues 256–291, 300–327, 332–357, 363–387, and 393–415, respectively) needed for transcription activation. Furthermore, a 100–255 region of ZIC2 known to be necessary for interaction with MDFIC and transcriptional activation or repression is predicted to have multiple disorder-based protein-protein interaction sites (see Figure S1D). Finally, similar to HOXD13 and HOAD13, deregulated expression of ZIC2 was shown to be associated with hepatocellular carcinoma, with this protein being required for the self-renewal maintenance of liver cancer stem cells [244].

Paired mesoderm homeobox protein 2B (PHOX2B homeodomain protein, UniProt ID: Q99453) is expected to have 54.14% disordered residues (as evaluated by the MobiDB-based consensus disorder content). In agreement with these MobiDB predictions, Figure 2E and Figure S1E show high levels of intrinsic disorder in N- and C-terminal regions of this protein. There are two poly-Ala tracts in human PHOX2B (residues 159–167 and 241–260) complemented by a poly-Gly region (residues 212–217). In addition to involvement in the poly-Ala expansion-related congenital central hypoventilation syndrome [245,246,247], mutations in PHOX2B are associated with neuroblastoma-2 [248,249]. Figure S1E shows that PHOX2B has several C-terminally-located disorder-based protein-protein interaction sites and also possess several PTM sites.

With its MobiDB consensus disorder score of 51.12%, transcription factor SOX3 (Sex-determining region Y-box3, UniProt ID: P41225) definitely belongs to the category of highly disordered proteins. This is further illustrated by Figure 2F and Figure S1F both showing high levels of intrinsic disorder almost evenly distributed throughout the entire protein sequence. Human SOX3 has a poly-Gly and poly-Pro tracts (residues 129–133 and 290–294, respectively) and four poly-Ala regions (residues 234–248, 324–330, 340–347, and 353–364). The protein is predicted to have 9 MoRF regions and multiple phosphorylation sites (see Figure S1F). Besides being associated with X-linked mental retardation with growth hormone deficiency (via its poly-Ala tract expansion mutations) [250], as well as with X-linked hypopituitarism (via its over- and under-dosage) [251] and SOX3 copy number variation-related 46, XX sex reversal 3 (SRXX3) [252], SOX3 overexpression was shown to play a crucial role in tumor progression [253,254,255,256,257], placing this transcription factors into the oncogene category.

Homeobox protein ARX (Aristaless-related homeobox, UniProt ID: Q96QS3) is predicted by MobiDB consensus to have 59.07% disordered residues. It is not surprising since ARX has two long Ala-rich regions (residues 100–155 and 425–544), as well as a long Pro-rich region (residues 395–459) and a long Glu-rich region (residues 224–253). In fact, Figure 2G and Figure S1G indicate that intrinsic disorder is spread over the entire protein sequence and Figure S1G shows that this disorder is of functional importance, since ARX is predicted to have 8 MoRFs (two of very significant length, 44 and 157 residues), as well as several phosphorylation and methylation sites. Again, besides being related to the poly-Ala expansion-driven X-linked mental retardation [258], mutations ARX are related to agenesis of the corpus callosum in females and X-linked lissencephaly with abnormal genitalia in males [259], early infantile epileptic encephalopathy-1 [260,261], Partington syndrome [261], and X-linked lissencephaly-2 [259,262]. It was also pointed out that duplication mutation of ARX can cause benign bilateral cystic-like cavities in the cerebral and cerebellar hemispheres [263].

Figure 2H and Figure S1H show that human forkhead box protein L2 (FOXL2, UniProt ID: P58012) is predicted to have very high levels of intrinsic disorder, possessing the MobiDB consensus disorder score of 47.34%. As a matter of fact, it is unlikely that any significant part of this protein can spontaneously gain ordered structure. Instead, several regions of FOXL2 can fold at interactions with specific binding partners and this protein is shown to have multiple sites of phosphorylation, acetylation and SUMOylation (see Figure S1H). As the majority proteins discussed in this section, human FOXL2 contains a poly-Gly, a poly-Pro, and two poly-Ala stretches (residues 35–43, 284–292, 221–234, and 301–304, respectively). Besides multiple point mutations several different poly-Ala tract expansions associated with blepharophimosis, ptosis, and epicanthus inversus syndrome [264] several mutations in this proteins are directly linked to the premature ovarian failure 3 [265,266]. Furthermore, C134W mutation in FOXL2 was shown to be one of the causative agents of the adult granulosa cell tumor, one of the malignant ovarian sex cord-stromal tumors [267].

A last member of the diverse family of proteins with pathogenic poly-Ala expansions is polyadenylate-binding protein 2/polyadenine-binding protein nuclear-1 (PABP2/PABPN1, UniProt ID: Q86U42) that is predicted to be highly disordered by MobiDB (consensus disorder score of 59.80%), PONDR^®^ VSL2 (Figure 2I) and D^2^p^2^ (Figure S1I). In fact, all computational tools agree that the first 160 and the last 60 residues of PABP2/PABPN1 are expected to be disordered, whereas central region consisting of residues 170–250 should be ordered. In agreement with these predictions, X-ray structure was solved for the RNA binding domain also known as RNA recognition motif (RRM) of this protein spanning residues 167–254 (see, e.g., PDB ID: 3B4D; [268]). Figure S1I suggests that N- and C-terminally located intrinsic disorder is differently used in functionality of PABP2/PABPN1, with long N-terminal IDPR clearly serving protein-protein interaction roles (it has all 6 MoRFs found in this protein and possesses multiple phosphorylation and ubiquitination sites), whereas disordered C-tail mostly functions in PABP2/PABPN1 regulation, possessing a whole host of methylation sites. In agreement with this hypothesis, known protein interaction sites, such as regions needed for interaction with SKIP (residues 2–145), stimulation of poly(A) polymerase alpha (PAPOLA, residues 119–147) and coiled-coil-based interaction (residues 115–151), are all located within the N-terminal IDPR. Being the only non-transcription factor with the pathogenic poly-Ala tract, PABP2/PABPN1 does not form an exception from the multipathogenicity rule, being associated with oculopharyngeal muscular dystrophy via the expansion mutations in its poly-Ala tract [269] and also being involved in metastatic duodenal cancer [270] and non-small cell lung cancer [271].

### 5.2. Disorder in Proteins Associated with PolyQ Expansion Diseases

Surprisingly, despite being a multi-pass transmembrane protein (it has six transmembrane α-helical regions, residues 293–313, 320–340, 371–391, 410–430, 459–479, and 528–548 and an intramembrane pore-forming region, residues 499–519), small conductance calcium-activated potassium channel protein 3 (SK3, UniProt ID: Q9UGI6) is predicted to be have high levels of intrinsic disorder (it has a MobiDB consensus disorder score of 37.50%). Figure 2J shows that disorder is preferentially concentrated within the 270 N-terminal residues. Since SK3 is one of the two proteins considered in this article for which D^2^P^2^ information is not available as of yet, we formed a relationship between the SK3 intrinsic disorder and function directly using the ANCHOR algorithm for prediction of the disorder-based protein-protein interaction sites [215,216]. This analysis revealed that human SK3 has six MoRFs, all located within the long disordered N-terminal tail (residues 1–29, 43–66, 86–149, 160–205, 210–228, and 235–241). The N-terminal region of SK3 has a long Q-rich region (residues 30–99) that includes two polyQ tracts (residues 30–41 and 67–85) and a Pro-rich region (residues 42–64). Furthermore, there are a polyQ and a poly-Ser regions in the C-terminal tail of the protein (residues 688–692 and 732–735, respectively). Besides being linked to schizophrenia via its polyQ tract expansion [272], SK3 is related to the pathogenesis of several types of cancer, being involved in regulation of human cancer cell migration and bone metastases [273,274,275,276].

According to the MobiDB analysis, human junctophilin-3 (JP-3, UniProt ID: Q8WXH2) has a consensus disorder score of 51.07%. This is in line with the outputs of PONDR^®^ VSL2 (Figure 2K) and D^2^P^2^ (Figure S1K), which clearly show very high levels of intrinsic disorder in this protein (especially in its C-terminal part). The N-tail of JP-3 contains a long Gly-rich region (residues 4–143), whereas an Ala-rich segment is located in the central part of this protein (residues 366–416). Furthermore, JP-3 contains a series of 8 MORN repeats (residues 15–37, 39–60, 61–82, 83–105, 107–129, 130–152, 288–310, and 311–333), which are membrane occupation and recognition nexus regions potentially involved in interaction with phospholipids and contributing to the binding of plasma membrane. The fact that there are 17 MoRFs and multiple phosphorylation sites in JP-3 (see Figure S1K) clearly shows that disorder is crucial for functionality of this protein. CAG/CTG expansion in the gene encoding junctophilin-3 is related to the Huntington’s disease-like 2 pathology [158,277,278].

Figure 2L and Figure S1L show that human huntingtin (UniProt ID: P42858) is predicted to be moderately disordered. In fact, according to the MobiDB consensus analysis, the disorder content of this protein is 19.1%, with the majority of disordered regions being concentrated in its N-terminal region. Importantly, this N-terminal region with a high disorder content is a home for the polyQ expansion track. Furthermore, the N-terminus of huntingtin is known to be responsible for interaction with several nuclear proteins such as HYPA/FBP-11, which functions in pre-mRNA processing (splicesome function) [279]; nuclear receptor co-repressor protein (NCoR) [280], which plays a role in the repression of gene activity; and p53 [281], a tumor suppressor involved in regulation of the cell cycle, and also contains multiple binding sites for other proteins with nuclear functions. The fact that huntingtin includes a P*X*DLS motif that serves as a binding site for the transcriptional corepressor C-terminal binding protein (CtBP) [282] suggests that this protein may also play a role in transcriptional repression. Huntingtin was shown to be a very promiscuous binder, being engaged in interaction with more than 200 proteins [283]. One of these huntingtin interactors, huntingtin yeast-two hybrid protein K (HYPK), was indeed identified as a typical IDP [283]. The major pathological involvement of huntingtin is its defining role in the Huntington’s disease development, which is therefore considered as a single gene degenerative disorder [284].

The *DRPLA* gene encoding for atrophin-1 (UniProt ID: P54259) is widely expressed in brain and other tissues [89,160,285]. Although the predicted molecular mass of the atrophin-1 is 124 kDa, this protein migrates on SDS-PAGE at about 200 kDa [286], suggesting the high levels of intrinsic disorder. In agreement with these experimental data are the results of disorder prediction for this protein by multiple computational tools. For example, MobiDB suggests that this protein contains 86.05% disordered residues, whereas Figure 2M and Figure S1M also illustrate a very disordered nature of human atrophin-1. Protein has both nuclear localization and export signals (residues 16–32 and 1033–1041, respectively) and multiple regions with composition biases: four poly-Pro regions (residues 302–305, 442–447, 509–512, and 709–712), three poly-Ser regions (376–382, 386–397, and 569–579), a Glu/Ser-rich region (residues 73–82), a poly-His region (residues 479–483), a polyQ tract (residues 484–502), an Ala/Arg-rich region (residues 807–820), and two Arg/Glu-rich regions with mixed charges (residues 821–832 and 930–939). Two regions of human atrophin-1 were established to play a role in interaction with BAIAP2 (residues 517–567) and FAT1 (residues 879–894) [287]. Figure S1M shows that almost the entire protein can be engaged in the disorder-based interactions with protein partners. Finally, atrophin-1 is heavily decorated with a multitude of different PTMs. Human atrophin-1 is related to dentatorubral-pallidoluysian atrophy via pathological expansion of its polyQ tract [288] and was also shown to be at the center of the protein-protein interaction network related to the serrated colorectal carcinoma [289].

Human androgen receptor (AR, UniProt ID: P10275) is a 919 residue-long protein that migrates in SDS-PAGE as a polypeptide with an apparent molecular weight of 110 kDa [290]. The protein can be separated on a modulating N-terminal domain (NTD) that includes functional AF1 transactivation domain (residues 142–485), a conserved centrally-located DNA-binding domain (DBD) consisting of two zinc-coordinated modules, and a C-terminally located ligand-binding domain (LBD) [290]. Human AR has a Gln-rich region (residues 58–120) that includes two polyQ tracts (residues 58–80 and 86–91), another polyQ stretch (residues 195–199), as well as a poly-Pro, a poly-Ala, and a poly-Gly region (residues 374–383, 398–404, and 451–473, respectively). All these regions are located within the NTD. According to MobiDB, AR has a consensus intrinsic disorder score of 54.13%. Intrinsic disorder is preferentially concentrated in the N-terminal half of this protein (see Figure 2N and Figure S1N). In agreement with these predictions, experimental analysis of a region of the androgen receptor N-terminal domain lacking the largest polyglutamine stretch, but containing the remaining repeats, showed that it lacked stable tertiary structure in aqueous solutions [164]. Detailed conformational studies using a combination of experimental and computational techniques revealed that the AF1 transactivation domain is in the molten globule-like conformation [291,292]. PolyQ tract expansion of AR is related to spinal and bulbar muscular atrophy X-linked 1 [293], whereas multiple mutations preferentially located within the C-terminal half of this protein are associated with several androgen insensitivity syndromes [294]. Furthermore, AR abnormalities are identified in benign prostatic hyperplasia and prostate cancer [294].

Ataxin-1 (UniProt ID: P54253) is a 815 residue-long chromatin-binding factor that repress Notch signaling [295], interacts with RNA via the 540–766 region [177] and is predicted by MobiDB to contain 54.97% disordered residues. Ataxin-1 has a self-association domain (residues 494–604) that partially overlaps with the AXH domain, a nuclear localization signal (residues 794–794) and a polyQ stretch (residues 197–225). The C-terminal region of this protein is known to interact with a ubiquitin-specific protease USP7 [296]. According to Figure 2O and Figure S1O, the N-terminal half of this protein is more disordered than its C-terminal half containing the AXH domain (residues 562–693) that is characterized by a significant sequence and structural similarity to the transcription factor HMG-box containing protein 1 (HBP1) [297]. The structure of this AXH domain is known (e.g., see PDB ID: 1OA8 [297]), which is the only structurally characterized part of ataxin-1. In fact, almost the entire 450-residue-long N-terminal domain is predicted to be mostly disordered, whereas in the C-terminal half, extensive intrinsic disorder is present in the C-tail region (residues 700–815). Figure S1O shows that both long N- and C-terminally located IDPRs contain multiple MoRFs and PTM sites. Expansion of a CAG trinucleotide repeat, which codes for glutamine in the ataxin-1, is the causing a factor of an autosomal dominant neurodegenerative disease, spinocerebellar ataxia type 1 (SCA1) [298]. Furthermore, this protein controls the epithelial-mesenchymal transition of cervical cancer cells [299].

A 1313 residue-long human ataxin-2 (UniProt ID: Q99700) is involved in EGF receptor trafficking [300]. It is predicted to have a MobiDB consensus disorder score of 79.13% and shows widespread disorder throughout the entire sequence that clearly has functional importance due to the presence of multiple MoRFs and astomishing number of PTM sites (Figure 2P and Figure S1P). The amino acid sequence of human ataxin-2 has multiple regions with strong compositional biases, such as three Pro-rich regions (residues 47–158, 551–734, and 929–1085), a poly-Pro stretch (residues 55–64), a polyQ tract (residues 166–187) and a poly-Ser region (residues 213–223). Ataxin-2 is a highly basic protein except for one acidic region (residues 254–475) that contains two predicted globular domains, Lsm (Like Sm, amino acid 254–345) and LsmAD (Lsm-associated domain, amino acid 353–475) [301]. The LsmAD domain contains a clathrin-mediated *trans*-Golgi signal (YDS, amino acid 414–416) and an endoplasmic reticulum (ER) exit signal (ERD, amino acid 426–428). This domain is composed mainly of α-helices according to the results from secondary structure prediction servers. The rest of ataxin-2 outside of the Lsm and LsmAD domains is only weakly conserved in eukaryotic ataxin-2 homologues and is predicted to be highly disordered [301]. Curiously, polyQ tract expansion in human ataxin-2 is associated with two neurodegenerative diseases, amyotrophic lateral sclerosis 13 associated with the intermediate expansions of CAG repeat (between 24 and 35 repeats) [302] and spinocerebellar ataxia 2 (SCA2) [92,93,303]. Furthermore, this protein may play a role in pathology of Parkinson’s disease likely via perturbations of RNA-binding and poly(A) RNA-binding functions of several groups of proteins [304,305]. It is also related to primary open-angle glaucoma susceptibility [306], and its levels are reduced in neuroblastoma tumors with amplified MYCN [307].

Human ataxin-3 (UniProt ID: P54252) is a 364 residue-long deubiquitinating enzyme with a wide spectrum of functions related to maintenance of protein homeostasis, cytoskeleton regulation, degradation of misfolded chaperone substrates, myogenesis, and transcription [308,309,310,311]. Despite being an enzyme that possesses a catalytic Josephin domain (residues 1–180), ataxin-3 is predicted to have really high levels of intrinsic disorder. Figure 2Q and Figure S1Q show that disorder is mostly contained within the C-terminal half of protein, which is characterized by the MobiDB-based consensus disorder score of 42.03%. Analysis of human ataxin-3 by a multitude of biophysical and biochemical techniques supported results of these computational analyses and suggested that this protein is indeed composed of a structured N-terminal domain followed by a flexible tail [312]. The Josephin domain is highly conserved from nematodes to human and is also found in plants [313]. The intrinsically disordered C-tail is non-conserved contains long stretches of low complexity regions [313], including a polyQ tract (residues 292–305) that contains 12–40 glutamines in normal individuals and is expanded to 55–84 glutamines in the pathogenic form associated with spinocerebellar ataxia 3 (SCA3) [136,137]. Importantly, besides its involvement in SCA3, ataxin-3 has some other pathological functions, with decreased expression being correlated with the clinicopathologic features of gastric cancer [314].

Voltage-dependent P/Q-type calcium channel subunit α1A (CACNA1A, UniProt ID: O00555) is a 2505 residue-long polypeptide with a multitude of transmembrane regions and two long cytoplasmic domains (residues 715–1242 and 1808–2505), both predicted to be highly disordered (see Figure 2R). This observation is also in line with the high MobiDB consensus disorder score of this protein (42.08%). CACNA1A is a second protein for which no disorder-related information is provided by D^2^P^2^. However, similar to many other proteins considered in this article CACNA1A is characterized by the presence of several regions with compositional biases, such as poly-Gly (residues 13–18), two poly-Glu stretches (residues 727–732 and 1204–1207), poly-Arg, poly-His, and poly-Pro regions (residues 1002–1007, 2211–2220, and 2221–2224, respectively) in addition to the polyQ tract (residues 2314–2324). Involvement of CACNA1A in spinocerebellar ataxia type-6 (SCA6) is related to the trinucleotide CAG repeat expansion within its exon 47 [315] from the normal 4–16 to the 21–28 pathological SCA6-related repeats [316]. Pathological CACNA1A mutations were found to be associated with familial hemiplegic migraine type-1 [317,318,319,320], episodic ataxia type-2 [317], and early infantile epileptic encephalopathy type-42 [321,322]. CACNA1A is also associated with the exfoliation syndrome, which is the most common cause of open-angle glaucoma worldwide [323]. Furthermore, bioinformatics meta-analysis of public microarray datasets revealed that together with other members of the voltage-gated calcium channel family, CACNA1A can be implicated in the development and progression of diverse types of cancer and might undergo dramatic up-regulation in breast cancer [324].

Ataxin-7 is a 892 residue-long protein (UniProt ID: O15265) serving as a component of the STAGA transcription coactivator-HAT complex [167] that includes SPT3, TAF9, ADA, and GCN5 acetyltransferase [325]. Although human protein has a molecular mass of 95.4 kDa, at the SDS-PAGE it migrates at about 110 kDa [325], suggesting that ataxin-7 possesses significant amount of intrinsic disorder. In agreement with these observations and similar to other ataxins, this protein is predicted to be highly disordered, being characterized by the MobiDB consensus disorder score of 71.30% and being mostly disordered in the PONDR^®^ VSL2 and D^2^P^2^ plots (see Figure 2S and Figure S1S). In agreement with these high levels of predicted intrinsic disorder, the amino acid sequence of human ataxin-7 is heavily enriched in regions with compositional biases, such as two poly-Ala (residues 16–20 and 23–28), Gln-rich (residues 30–49), polyQ (residues 30–49), two Pro-rich (residues 40–65 and 402–486), two poly-Pro (residues 40–65 and 51–55), and two Ser-rich regions (residues 171–219 and 640–851) containing five poly-Ser tracts (residues 171–174, 213–219, 647–654, 717–730, and 840–845). Expansions of the polyQ tract from 4–35 to 36–306 repeats are associated with the spinocerebellar ataxia type-7 (SCA7) [326]. Furthermore, Lys264Arg mutation in ataxin-7 is among several common non-synonymous SNPs associated with breast cancer susceptibility [327], and a fusion between ataxin-7 and DNA repair protein Rad51C is expressed in colorectal tumors [328], whereas spleen-specific isoform of ataxin-7 was suggested to serve as a potential marker of the lymphoma-affected spleen [329].

The TATA-box-binding protein (TBP, UniProt ID: P20226) is a 339 amino acid-long general transcription factor engaged in the formation of the DNA-binding multiprotein factor TFIID related to the activation of eukaryotic genes transcribed by RNA polymerase II [330,331,332,333,334], as well as several other transcription factor complexes, such as a BRF2-containing transcription factor complex regulating the RNA polymerase III-mediated transcription [335] and the SL1/TIF-IB complex engaged in the assembly of the pre-initiation complex (PIC) during RNA polymerase I-dependent transcription [336]. Therefore, being required for transcriptional initiation by the three major RNA polymerases (RNAP I, II, and III) in eukaryotic nuclei, TBP is involved in the expression of most eukaryotic genes [337]. A part of the polyQ expansion-related pathology is abnormal interaction of TBP with the general transcription factor TFIIB and reduced DNA binding [338]. As many other transcription factors, TBP is predicted to be highly disordered (see Figure 2T and Figure S1T), possessing a wholly disordered N-tail (residues 1–160) that contains a polyQ tract (residues 55–95), and being characterized by the overall MobiBD consensus disorder score of 46.31%. This disorder distribution within the TBP sequence follows its domain organization, with the C-terminal domain that mediates virtually all of the transcriptionally relevant interactions of TBP being highly conserved among eukaryotes and [339], and with the N-terminal domain being evolutionarily divergent and showing sequence conservation only in vertebrates. In agreement with high levels of intrinsic disorder, human TBP, a protein with the calculated molecular mass of 37.7 kDa, was shown to possess an apparent molecular mass of ~49 kDa [340].

### 5.3. Disorder in Proteins Associated with Non-Coding Region Repeat Expansions

Since these proteins do not have pathological expansions in their coding regions, their intrinsic disorder status will be considered very briefly, with the exception being made for C9orf72, because of the known fact that although the GGGGCC hexanucleotide repeat expansion is located within the non-coding region of the *C9ORF72* gene, it can be bi-directionally transcribed, and both sense and antisense repeat RNAs can be translated into the dipeptide repeat proteins (DPRs or C9RANT proteins) via the repeat-associated non-ATG (RAN) translation.

Synaptic functional regulator FMR1 (UniProt ID: Q06787) is a 632 residue-long polyribosome-associated RNA-binding protein regulating alternative mRNA splicing, mRNA stability, mRNA dendritic transport and postsynaptic local protein synthesis of a subset of mRNAs [341,342,343,344,345] among a myriad of other functions. FMR1 is predicted to be rather highly disordered, possessing MobiDB score of 38.92% and highly disordered C-terminal region that has multiple MoRFs and is heavily decorated with different PTMs (see Figure 2U and Figure S1U). However, it does not possesses regions with compositional bias.

Disco-interacting protein 2 homolog B (UniProt ID: Q9P265) is a moderately disordered 1576 residue-long protein involved in DNA methylation [346], with the MobiDB score of 19.73% and highly disordered N-terminal tail with several disorder-based protein binding regions and multiple sites of different PTMs (see Figure 2V and Figure S1V). This protein has a Ser-rich (residues 145–234), a poly-Ala (residues 1118–1121), and two poly-Val regions (residues 1503–1506 and 1534–1540).

AF4/FMR2 family member 2 (UniProt ID: P51816) is a 1311 residue-long RNA binding protein involved in alternative splicing regulation [347]. FMR2 is predicted by MobiDB to have 58.12% disordered residues occupying the N-terminal and central parts of this protein that have multiple disorder-based binding regions and several PTM sites (see Figure 2W and Figure S1W). There is no compositional bias in this protein.

Although the 481 residue-long protein C9orf72 (UniProt ID: Q96LT7) is the most ordered protein analyzed in this study (it has a MobiDB score of 2.70% and is expected to have short disordered tails, three to four short disordered loops, and a long disordered/flexible region located between the residues 130 and 210 as shown in Figure 2X and Figure S1X), it clearly deserves more attention in relation to the topic of this article. Besides the fact that the GGGGCC (G_4_C_2_) hexanucleotide repeat expansions in the non-coding intronic region between the non-coding exons 1a and 1b of the *C9ORF72* gene represent the major cause of ALS and FTD [348,349] and that these expansions can vary from 10 to thousands of repeats [108,109,181,350,351], the expanded GGGGCC hexanucleotide repeats can be bi-directionally transcribed, and both sense and antisense repeat RNAs are engaged in the formation of RNA foci [352,353,354,355]. Furthermore, resulting hexanucleotide repeat RNA can be translated in a series of the dipeptide repeat proteins (DPRs or C9RANT proteins) by the RAN translation and these DPRs are commonly found as major constituents of proteinaceous inclusions throughout the CNS of the ALS and FTD patients [356]. This RAN translation of sense GGGGCC repeat RNAs generates three different proteins, poly(GA), poly(GR), and poly(GP) [194,195], and poly(PA), poly(PR), and poly(GP) proteins are synthesized as a result of translation of the antisense RNAs [353,355,357]. It is known that repeat-containing proteins are often intrinsically disordered, with the more perfect repeats being more disordered [47]. In agreement with these earlier observations, all DPRs synthesized as a result of the RAN translation of the sense and antisense GGGGCC hexanucleotide repeat RNAs, poly(GA), poly(GR), poly(GP), poly(PA), and poly(PR), were predicted to be highly disordered [358]. It was also emphasized that based on their positions within the CH-CDF phase space, DPRs can be classified either as native molten globules (Poly(GA) and Poly(PA)) or native coils or pre-molten globules (Poly(GP), Poly(GR), and Poly(PR)) [358].

Frataxin is a 210 residue-long iron-binding protein (UniProt ID: Q16595) that takes part in the heme biosynthesis [359] and biosynthesis and repair of iron-sulfur clusters [360] and acts as an iron chaperone [361]. Despite being involved in catalytic detoxification of redox-active iron [362], frataxin is predicted to have a MobiDB score of 40.00%, with the majority of disordered residues being concentrated within the N- and C-tails containing several MoRFs and PTMs (see Figure 2Y and Figure S1Y). There are no regions with compositional bias in this protein.

The cellular nucleic acid-binding protein is a 177 residue-long single-stranded DNA-binding protein (CNBP, UniProt ID: P62633) with moderate level of intrinsic disorder as evidenced by the MobiDB score of 19.77% and presence of several IDPRs enriched in MoRFs and PTM sites (see Figure 2Z and Figure S1Z). CNBP has an Arg/Gly-rich region (residues 22–42) seven zinc finger motifs of the CCHC type.

Ataxin-10 is a 475 residue-long protein (UniProt ID: Q9UBB4) needed for the survival of cerebellar neurons. With the MobiDB score of 5.26%, mostly short IDPRs (see Figure 2a and Figure S1a), and lack of compositional biases, this protein is the most ordered ataxin considered in this article.

Nucleolar protein 56 is a 594 residue-long protein (UniProt ID: O00567) serving as a core component of the box C/D small nucleolar ribonucleoprotein (snoRNP) particles [363] and related to the early to middle stages of the biogenesis of 60S ribosomal subunit [364]. It is characterized by the moderate-to-high overall levels of intrinsic disorder, has a MobiDB score of 26.26%, and possesses a highly disordered C-terminal domain that contains multiple MoRFs, a multitude of divers PTMs (see Figure 2b and Figure S1b), and a long Lys-rich stretch (residues 438–589).

Transcription factor 4 is a 667 residue-long transcription factor (UniProt ID: P15884) known for its binding to the immunoglobulin enhancer Mu-E5/KE5-motif and involvement in the initiation of neuronal differentiation. This is one of the most disordered proteins analyzed in this study, being characterized by a MobiDB score of 88.01%, containing long IDPRs and a whole host of different PTMs, using almost an entire sequence for the disorder-based protein-protein and protein-DNA interactions (see Figure 2c and Figure S1c), and containing a short poly-Ser region (residues 228–231).

Myotonin-protein kinase is a 629 residue-long non-receptor serine/threonine protein kinase (UniProt ID: Q09013) needed for the upkeep of skeletal muscle structure and function. It has a moderate intrinsic disorder content of 14.63%, with the majority of disordered residues, disorder-based binding regions, and PTM sites being present in the C-terminal part of this protein (see Figure 2d and Figure S1d). There are no compositional bias regions in this kinase.

Ataxin-8 and ATXN8OS are 80 and 200 residue-long proteins, respectively. Biological functions of the ataxin-8 (UniProt ID: Q156A1) and ATXN8OS proteins (UniProt ID: P0DMR3) are unknown. ATXN8OS is a putative protein with the disorder content of 68% (see Figure 2e). It is predicted to contain a couple of MoRFs located in the N-terminal region. Since ataxin-8 is simply a polyglutamine polypeptide (in fact, according to UniProt, it contains only 80 glutamine residues); it is not surprising that this protein is predicted to be completely disordered (see Figure 2f).

Cystatin-B is a short, 98 residue-long, protein (UniProt ID: P04080) that serves as an inhibitor of intracellular thiol proteinase. Cystatin-B is predicted by MobiDB to have 40.82% disordered residues and is expected to have flexible tails and a less flexible central region (see Figure 2g and Figure S1g).

Serine/threonine-protein phosphatase 2A 55 kDa regulatory subunit B β isoform (PPP2R2B, UniProt ID: Q00005) is a 443 residue-long protein modulating substrate selectivity and catalytic activity of the PP2A phosphatase. It is characterized by a MobiDB score of 7.67% and has several short IDPRs, one short MoRF, and several PTM sites (see Figure 2h and Figure S1h). There are also seven WD repeats that are 40-residue-long conserved domains containing a centrally located Trp-Asp motif.

## 6. Summary

IDPs and IDPRs are characteristically comprised of low complexity domains, often containing repetitive amino acid sequences [47]. Expansion of these repetitive domains frequently results in pathology seen in several neurological and neuromuscular diseases. Many of the genes linked to repeat expansion diseases are translated into proteins predicted to contain a large percentage of disordered regions. Even expansions in regions that are not normally translated, such as those in C9orf72, result in aberrant dipeptide repeat products predicted to be disordered. The resultant disorder in these peptides often decreases in a length-dependent manner, causing many repetitive proteins to aggregate and sequester other proteins into the aggregated structures [365].

Trinucleotide repeats are the most common type seen in coding regions of genes, with polyQ being the most commonly occurring followed by polyAla [65]. Both pathological extensions result in diseases classified as protein misfolding disorders and share many pathological characteristics such as intracellular protein inclusions and the appearance of RNA foci. Since both Ala and Gln repeat expansions correlate with protein aggregation and formation of the β-structure-enriched amyloid-like fibrils [366,367], it is likely that the fibrillation process triggered by expanded repeats of these two completely different residues has some common molecular mechanisms. In agreement with this hypothesis, computational analysis of non-aggregated polyAla and polyQ peptides composed of 7, 10, 14 or 20 amino acids revealed that both types of these homo-oligopeptides are characterized by the presence of similar secondary structural elements (type I and type III β-turns, antiparallel β-strand, α-helix, and 3_10_-helix) and that characteristic H-bonding patterns containing i–i + 3 and i–i + 4 H-bonds are formed [368]. Furthermore, both polyAla and polyQ repeats were shown to form coiled-coil structures, the stability of which increased with the length of the expansion, and which were also able to form higher-order multimers and aggregates in vitro [369]. Also, it was pointed out that in the majority of expanded CAG and GCG repeat proteins, the polyQ or polyAla sequence is typically located within the protein, thereby possessing specific N- and C-terminal flanking sequences that may play a crucial role in regulation of the polyQ and polyAla aggregation propensity [367]. Finally, many of the proteins involved in both types of trinucleotide repeat expansions are associated with signaling, regulation, and RNA metabolism. Many of the diseases associated with both expansion types are developmental, neuromuscular, and neurodegenerative in nature [365].

The similarities shared by the two common trinucleotide repeats involved in disease are more abundant than the characteristics that set them apart. For example, trinucleotide repeats exceeding specific thresholds show replication-related instability that increases in a repeat length-dependent manner, with most instabilities causing repeat expansion [370]. Due to the effect of increased probability of repeat expansion on replication, and due to the fact that the increase in the rate of mutations that add additional codons and thereby increase the expansion length become more likely with each new generation, in successive generations, the age of the onset of trinucleotide expansion diseases typically becomes younger, whereas the severity of these maladies increases [370]. Importantly, one should keep in mind that expanded repeats can undergo further expansion-biased somatic instability, leading to further increases in the expansion length [371,372,373,374,375]. Furthermore, pathogenic processes associated with the expansions of both the polyQ and polyAla repeats are usually age-dependent (i.e., they do not happen before a particular age). This is likely due to the presence of the aforementioned somatic instability of the trinucleotide expansions [372] and because of other age-related processes, such as impairment of proteostasis [376,377,378,379,380,381].

However, not everything is similar for the repeat-containing proteins. In fact, one stark difference between polyQ and polyAla repeats is the threshold size of the repeat needed to cause disease. PolyQ repeats require much longer extensions for disease presentation, while polyAla repeats often can causes disease with negligible extensions. This size difference may have to do with the fact that extensions in polyAla repeats induce structural changes more readily than those in polyQ repeats because of the biophysical properties of the repeated amino acids in the peptide sequence [65].

Repeats in non-coding regions are more diverse than those seen in coding regions. The functions of the genes that are involved in disease vary widely as compared to those found in coding regions that are involved in disease. Often times in non-coding expansion diseases, there is a knock down in expression of the gene involved. However, in most cases this is not sufficient to cause disease and therefore there must be other mechanisms at play. For example, we now know that in the case of C9orf72, not only is the expression of the protein reduced, but aberrant mRNA and repeat peptide species are also produced [108,109,181].

The peptide species produced through non-canonical translation of the repeat region in C9orf72 are predicted to be disordered at short repeat lengths, but upon extension have been shown to form toxic stable aggregates in cells that cause the sequestration of proteins with low complexity domains [196]. Therefore, it is important to consider that these species may account for, at least in part, the pathology that is associated with the expansion of repeats in non-coding regions. In addition, it is important to note that the expansion in the 5’ UTR of *C9orf72* results in five different dipeptide repeat species. Three of the peptides are produced from the three available reading frames of the repeat region and their antisense partners make up the remaining three species present in disease. This could theoretically be the case for all expansions that occur in non-coding regions and should be considered for other similar cases. All of the repeat regions may also have the propensity to undergo translation into toxic repeat peptide species with unique biochemical properties. The predicted peptide species for each non-coding expansion linked to disease can be found in Table 2. Although, except for the DPRs generated as a result of RAN translation of the mRNA produced as a result of hexanucleotide expansion in the 5’ UTR of the *C9orf72* gene, the presence of such polypeptides was not demonstrated as of yet, so one cannot exclude that at least some of these species are present in cells affected by the non-coding expansion mutations and can therefore contribute to the pathology of related diseases.

Curiously, it has been demonstrated that the repeat-associated non-ATG translation can occur not only for the mRNAs produced as a result of the non-coding region repeat expansions, but also for other RNAs containing expanded CAG and CTG trinucleotide repeats, resulting in expression of homopolymeric expansion proteins in all three reading frames [382]. Among characteristic examples of these RAN translation events are biosynthesis of homopolymeric polyglutamine, polyalanine, and polyserine proteins in the absence of an ATG codon. It was also pointed out that polyAla and polyserine proteins can contribute to the pathogenesis of some of the CAG expansion-associated polyQ diseases [382]. For example, in SCA8, SCA8_GCA-Ala_ expansion protein was found in cerebellar Purkinje cells, whereas in DM1, the DM1_CAG-Gln_ expansion protein was found in heart, myoblasts, and skeletal muscles [382].

Diseases found in microsatellite expansion regions are numerous and, in most cases, devastating. The broad commonalities between the functions of the genes involved, the structural change of the translated products as a result of the expansion, and the resulting disease phenotypes are overwhelmingly evident. This points to the question of whether there is a shared mechanism that occurs in most disease as a result of the expansions. Elucidation of the common mechanisms involved in pathology of each expansion disorder may shed light on this question and open new avenues for a broader therapeutic approach to treating repeat expansion disorders.

## Figures and Tables

**Table 1:** Major characteristics of genes with pathological repeat expansions and proteins they encode.

	Repeat Location	Gene	Disease ^a^	Repeat Sequence	WT Length	Pathogenic Length ^b^	% Disorder ^c^	References
Poly-alanine	Exon	*HOXD13*	SPD II	GCG	15	>21	33.82	[64,65,66]
	Exon	*HOXA13*	HFGS	GCG	12	>17	34.28	[67]
	Exon	*RUNX2*	CCD	GCG	17	>26	62.96	[65,68]
	Exon	*ZIC2*	HPE	GCG	9	--	54.89	[69,70]
	Exon	*PHOX2B*	CCHS	GCG	20	--	54.15	[71,72,73]
	Exon-X chrom.	*SOX3*	XLMR + GHD	GCG	15	>25	51.12	[74,75]
	Exon-X chrom.	*ARX*	XLMR	GCG	16	>17, >22	59.07	[76,77]
	Exon	*FOXL2*	BPEIS	GCG	14	>21, >24	47.34	[78,79]
	Exon	*PABPN1*	OPMD	GCG	10	>11, >16	59.80	[80,81]
Poly-glutamine	Exon	*KCNN3*	Schizo. ^d^	CAG	--	--	37.50	[82]
	Exon	*JPH3*	HDL2	CAG/CTG	6 to 28	>41	51.07	[83,84,85]
	Exon	*HTT*	HD	CAG	6 to 35	>35	19.10	[86,87]
	Exon	*ATN1*	DRPLA	CAG	3 to 36	>48	86.05	[88,89]
	Exon	*AR*	SBMA	CAG	9 to 36	>37	54.13	[90]
	Exon	*ATXN1*	SCA1	CAG	6 to 39	>39	54.97	[91]
	Exon	*ATXN2*	SCA2	CAG	14 to 32	>33	79.13	[92,93]
	Exon	*ATXN3*	SCA3	CAG	12 to 40	>54	42.03	[82,94]
	Exon	*CACNA1A*	SCA6	CAG	4 to 18	>20	42.08	[95,96]
	Exon	*ATXN7*	SCA7	CAG	7 to 17	>33	71.30	[97,98]
	Exon	*TBP*	SCA17	CAG	25 to 42	>44	46.31	[99,100]
Non-coding	5′ UTR	*PPP2R2B*	SCA12	CAG	7 to 32	>54	7.67	[101,102]
	5′ UTR-X chrom.	*FMR1*	FXMR, FXTAS	CGG	6 to 55	>200, >55	38.29	[103]
	5′ UTR	*DIP2B*	FRA12A MR	CGG	6 to 23	>200	19.73	[104]
	5′ UTR-X chrom.	*FMR2*	FRAXE MR	GCC	--	>200	58.12	[105,106,107]
	5′ UTR	*C9orf72*	C9ALS/FTD	GGGGCC	--	Unknown	2.70	[108,109]
	Intron	*FXN*	FRDA	GAA	7 to 22	>66	40.00	[110,111]
	Intron	*CNBP/zfn9*	DM2	CCTG	<27	>75	19.77	[112,113]
	Intron	*ATXN10*	SCA10	ATTCT	10 to 29	>279	5.26	[114,115]
	Intron	*NOP56*	SCA36	GGCCTG	3 to 8	>1500	26.26	[116]
	Intron	*TCF4*	FECD	CTG	--	>50	88.01	[117,118]
	3′ UTR	*DMPK*	DM1	CTG	5 to 37	>50	14.63	[119,120]
	3′ UTR	*ATXN8OS*	SCA8 ^e^	CTG	6 to 37	>74	68.00 ^f^	[121,122,123]
	Exon	*ATXN8*	SCA8 ^e^	CAG	15 to 50	71 to 1300	100.00 ^f^	[124]
	Promoter	*CSTB*	EPM1	CCCCGCCCCGCG	2 to 3	>14	40.82	[125,126]

^a^ SPD II, synpolydactyly; HFGS, hand-foot genital syndrome; CCD, cleidocranial dysplasia; HPE, holoprosencephaly cephalic disorder; CCHS, congenital central hypoventilation syndrome; XLMR + GHD, X-linked mental retardation with isolated growth hormone deficiency; XLMR, ARX-nonsyndromic X-linked mental retardation; BPEIS, blepharophimosis, ptosis, and epicanthus inversus syndrome; OPMD, oculopharyngeal muscular dystrophy; Schizo., schizophrenia; HDL2, huntinton’s disease-like 2; HD, Huntington’s disease; DRPLA, dentatorubral-pallidoluysian; SBMA, spinal and bulbar muscular atrophy; SCA, spinocerebellar ataxia; FXMR, fragile X mental retardation; FTXAS, fragile X-associated tremor/ataxia syndrome; FRA12A MR, fragile X mental retardation; FRAXE MR, fragile X mental retardation; ALS, amyotrophic lateral sclerosis; FTD, frontotemporal dementia; FRDA, Friedreich ataxia; DM, myotonic dystrophy; FECD, fuchs endothelial corneal dystrophy; EPM1, myoclonus epilepsy of Unverricht-Lundborg type, WT and pathogenic length refer to number of sequence repeats. ^b^ Pathogenic length indicates the threshold of the repeat length, above which the protein-carrier will cause development of pathology. ^c^ MobiDB-based predicted consensus disorder content is shown for query proteins (http://mobid.bio.unipd.it/) [127,128]. ^d^ Although CAG repeat tract length in *KCNN3* was correlated with schizophrenia, this is not a pathological repeat expansion and a cause of disease. ^e^ SCA8 is caused by the bidirectional transcription at the SCA8 locus containing *ATXN8OS* and *ATXN8* genes and therefore considered as the ′CTG*CAG′ repeat expansion disease, referring to the complementary base pairs of the *ATXN8OS* and *ATXN8* genes. ^f^ For ATXN8OS and ataxin-8 proteins, disorder content was calculated as an averaged value of the overall percent of residues predicted to be disordered by PONDR^®^ VLXT, PONDR^®^ VL3 and PONDR^®^ VSL2.

**Table 2:** Potential translation products of non-coding repeat expansions.

Gene	Repeat Sequence	Sense Translation	Antisense Translation
*FXS*	CGG-CGG-CGG-CGG	R-R-R-R	A-A-A-A
	GGC-GGC-GGC-GGC	G-G-G-G	P-P-P-P
	GCG-GCG-GCG-GCG	A-A-A-A	R-R-R-R
*DIP2B*	CGG-CGG-CGG-CGG	R-R-R-R	A-A-A-A
	GGC-GGC-GGC-GGC	G-G-G-G	P-P-P-P
	GCG-GCG-GCG-GCG	A-A-A-A	R-R-R-R
*FMR2*	GCC-GCC-GCC-GCC	A-A-A-A	R-R-R-R
	CCG-CCG-CCG-CCG	P-P-P-P	G-G-G-G
	CGC-CGC-CGC-CGC	R-R-R-R	A-A-A-A
*C9orf72*	GGG-GCC-GGG-GCC	G-A-G-A	P-R-P-R
	GGG-CCG-GGG-CCG	G-P-G-P	P-G-P-G
	GGC-CGG-GGC-CGG	G-R-G-R	P-A-P-A
*FXN*	GAA-GAA-GAA-GAA	E-E-E-E	L-L-L-L
	AAG-AAG-AAG-AAG	K-K-K-K	F-F-F-F
	AGA-AGA-AGA-AGA	R-R-R-R	S-S-S-S
*CNBP*	CCT-GCC-TGC-CTG	P-A-C-L	G-R-T-G-G
	CTG-CCT-GCC-TGC	L-P-A-C	G-G-R-T-G
	TGC-CTG-CCT-GCC	C-L-P-A	T-G-G-G-R
*ATXN10*	ATT-CTA-TTC-TAT-TCT	I-L-F-F-T	STOP-D-K-I-R
	TTC-TAT-TCT-ATT-CTA	F-F-S-I-L	K-I-R-STOP-D
	TCT-ATT-CTA-TTC-TAT	S-I-L-F-F	R-STOP-D-K-I
*TCF4*	CTG-CTG-CTG-CTG	L-L-L-L	D-D-D-D
	TGC-TGC-TGC-TGC	C-C-C-C	P-P-P-P
	GCT-GCT-GCT-GCT	A-A-A-A	R-R-R-R
*DMPK*	CTG-CTG-CTG-CTG	L-L-L-L	D-D-D-D
	TGC-TGC-TGC-TGC	C-C-C-C	P-P-P-P
	GCT-GCT-GCT-GCT	A-A-A-A	R-R-R-R
*JPH3*	CTG-CTG-CTG-CTG	L-L-L-L	D-D-D-D
	TGC-TGC-TGC-TGC	C-C-C-C	P-P-P-P
	GCT-GCT-GCT-GCT	A-A-A-A	R-R-R-R
*ATXN8*	CTG-CTG-CTG-CTG	L-L-L-L	D-D-D-D
	TGC-TGC-TGC-TGC	C-C-C-C	P-P-P-P
	GCT-GCT-GCT-GCT	A-A-A-A	R-R-R-R
*CSTB*	CCC-CGC-CCC-GCG	P-R-P-A	G-A-G-R
	CCC-GCC-CCG-CGC	P-A-P-R	G-R-G-A
	CCG-CCC-CGC-GCC	P-P-R-A	G-G-A-R

## Supplementary Materials

Supplementary materials are available online. Figure S1: Intrinsic disorder propensity and some important disorder-related functional information generated for human proteins encoded by genes with nucleotide expansions by the D2P2 database (http://d2p2.pro/). Here, the outputs of several disorder predictors are shown by differently colored bars, whereas the blue-green-and-white bar in the middle of the plot shows the predicted disorder agreement between nine predictors, with blue and green parts corresponding to disordered regions by consensus. Yellow bar shows the location of the predicted disorder-based binding sites (molecular recognition features, MoRFs), whereas colored circles at the bottom of the plot show location of various PTMs.
