# Supplementary material for: Intrinsic Disorder in Proteins with Pathogenic Repeat Expansions
Source: Molecules. 2017 Nov 24;22(12):2027. doi: 10.3390/molecules22122027 (PMC6149999; doi:10.3390/molecules22122027)
Supplement: Supplementary file 1 [file molecules-22-02027-s001.pdf]

Supplementary Materials

Intrinsic disorder in proteins with pathogenic repeat expansions

April L. Darling[1,2,*] and Vladimir N. Uversky[1,3,*]

[1]*Department of Molecular Medicine and USF Health Byrd Alzheimer's Research Institute, Morsani College of Medicine, University of South Florida, Tampa, FL 33612, USA*

[2]*James A. Haley Veteran's Hospital, Tampa, FL, 33612, USA*

[3]*Institute for Biological Instrumentation of the Russian Academy of Sciences, Pushchino, Moscow region, 142290, Russia*

*Corresponding authors: A.L.D., Tel: 1-813-396-9249; E-mail: aldarlin@mail.usf.edu; V.N.U., Tel: 1-813-974-5816; E-mail: vuversky@health.usf.edu

Figure S1. Intrinsic disorder propensity and some important disorder-related functional information generated for human proteins encoded by genes with nucleotide expansions by the D^2P^2 database (http://d2p2.pro/) (Oates et al., 2013). Here, the outputs of several disorder predictors are shown by differently colored bars, whereas the blue-green-and-white bar in the middle of the plot shows the predicted disorder agreement between nine predictors, with blue and green parts corresponding to disordered regions by consensus. Yellow bar shows the location of the predicted disorder-based binding sites (molecular recognition features, MoRFs), whereas colored circles at the bottom of the plot show location of various PTMs.

References

Oates, M.E., Romero, P., Ishida, T., Ghalwash, M., Mizianty, M.J., Xue, B., Dosztanyi, Z., Uversky, V.N., Obradovic, Z., Kurgan, L., Dunker, A.K., Gough, J., 2013. D(2)P(2): database of disordered protein predictions. Nucleic Acids Res 41, D508-516.

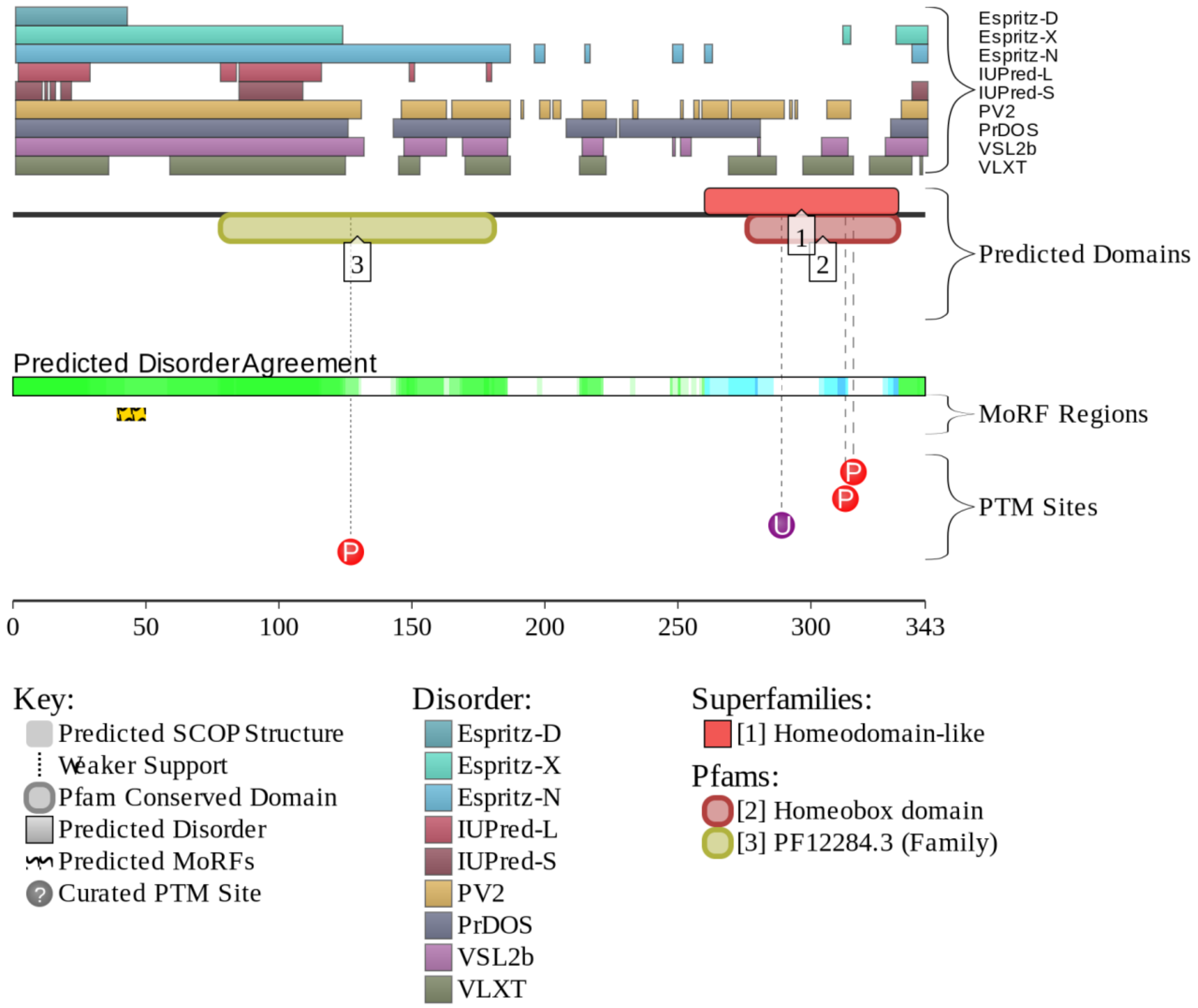

Figure S1A. D^2P^2 output for homeobox protein HOXD13 (UniProt ID: P35453)

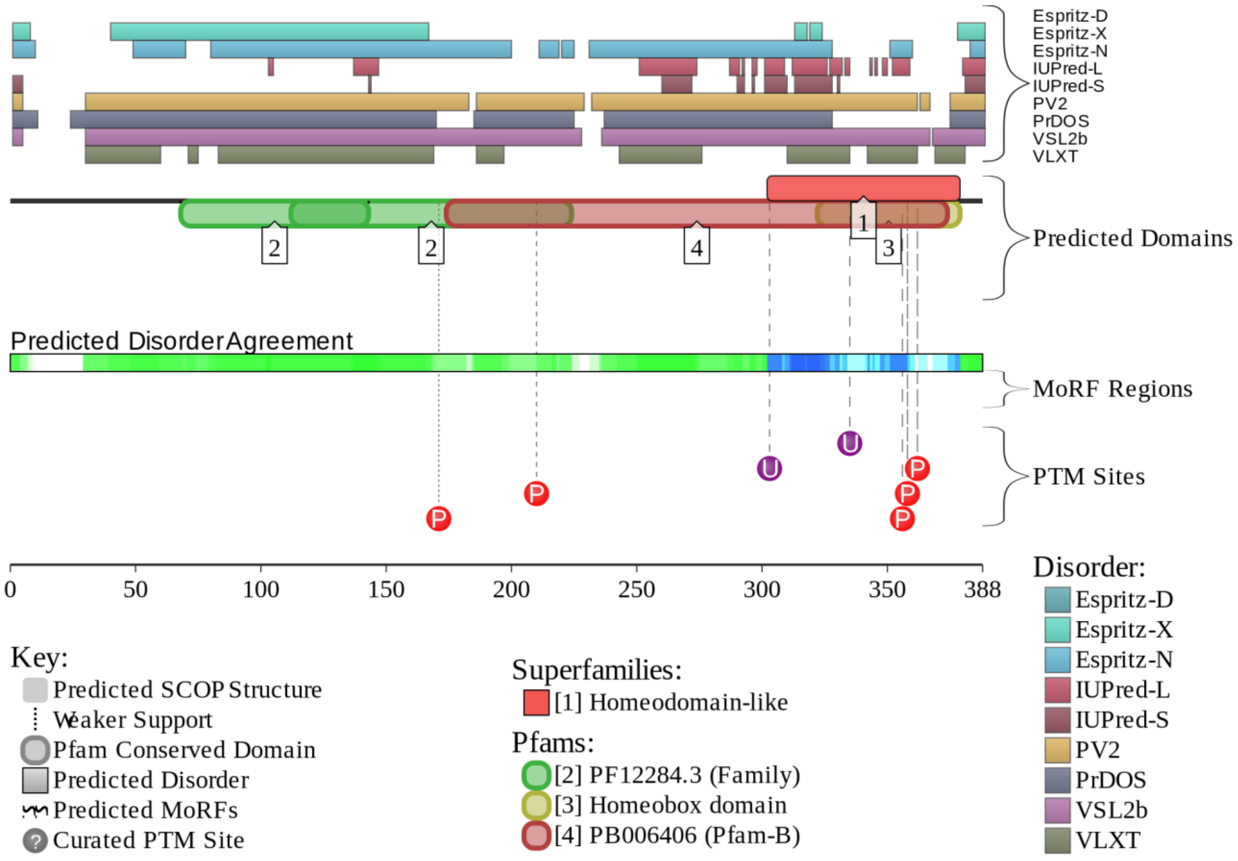

Figure S1B. D^2P^2 output for the homeobox protein HOXA13 (UniProt ID: P31271)

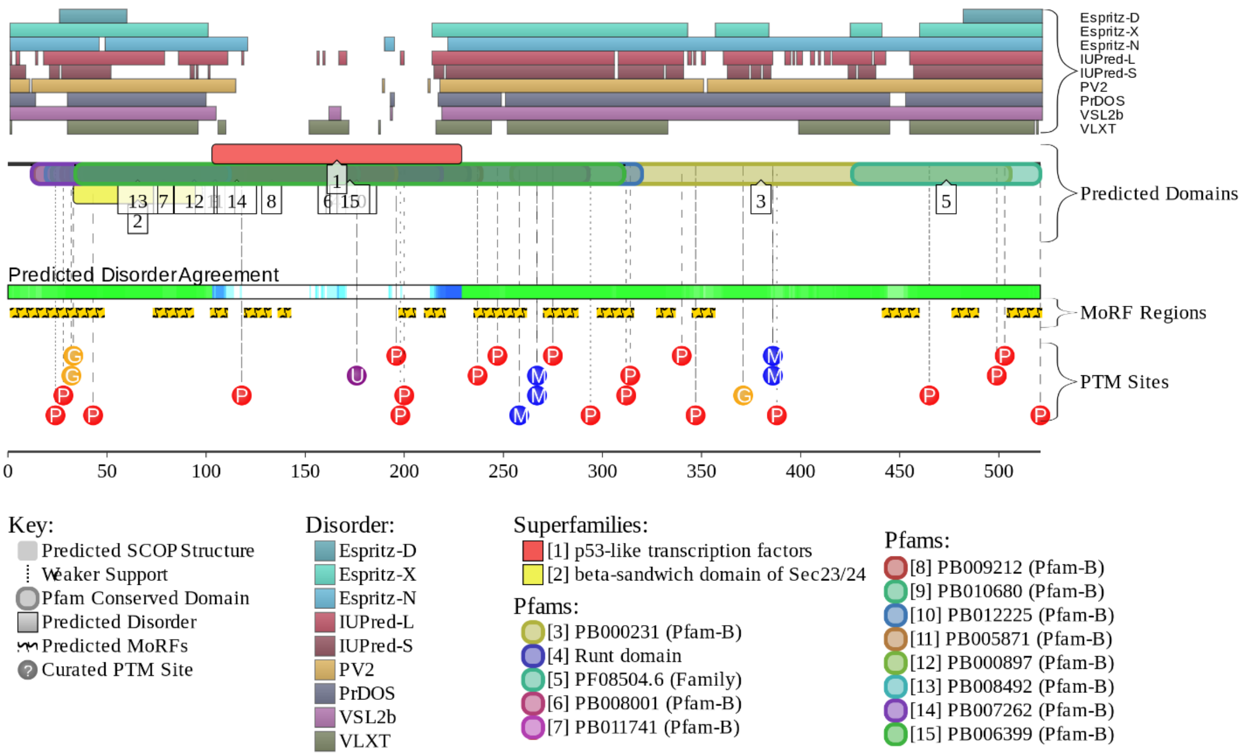

Figure S1C. D^{2}P^2 output for runt-related transcription factor 2, RUNX2 (UniProt ID: Q13950)

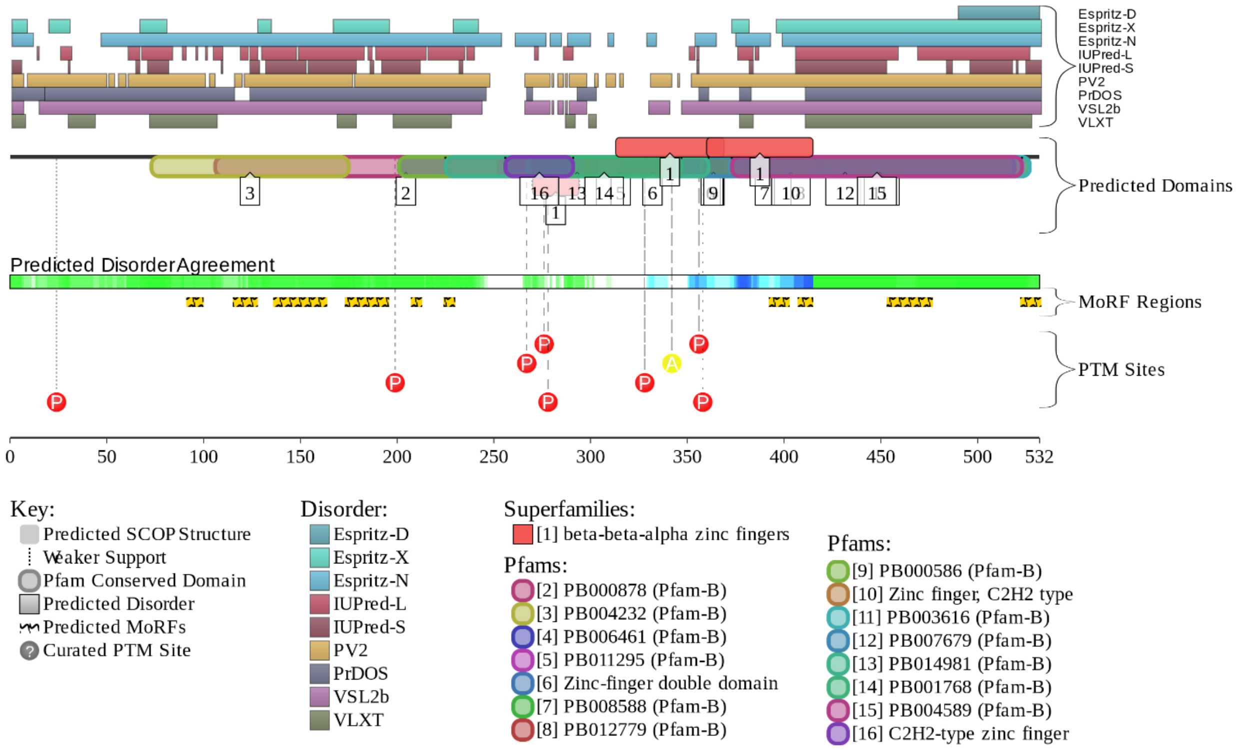

Figure S1D. D^2P^2 output for zinc finger protein ZIC2 (UniProt ID: O95409)

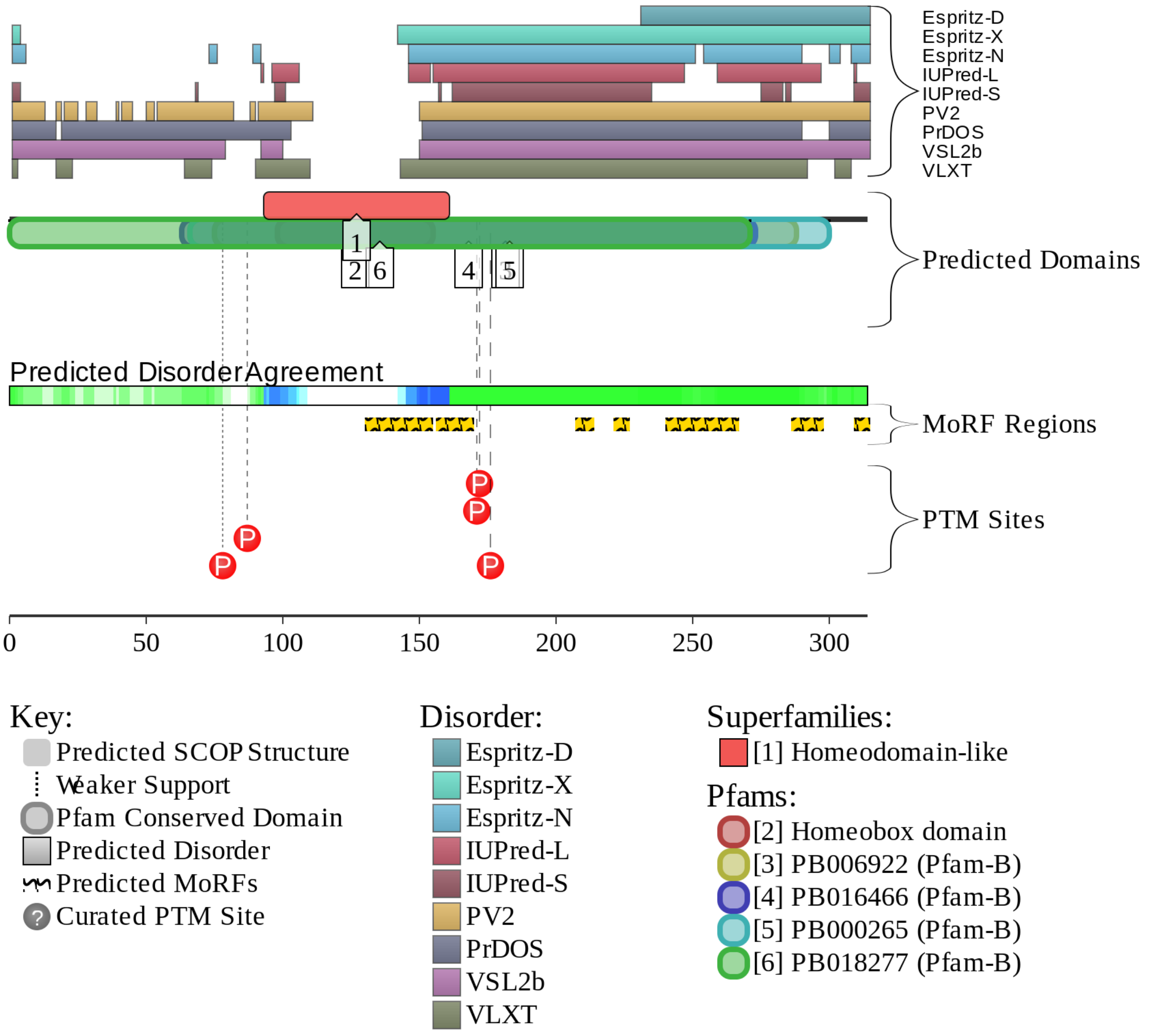

Figure S1E. D^2P^2 output for paired mesoderm homeobox protein 2B (PHOX2B homeodomain protein, UniProt ID: Q99453)

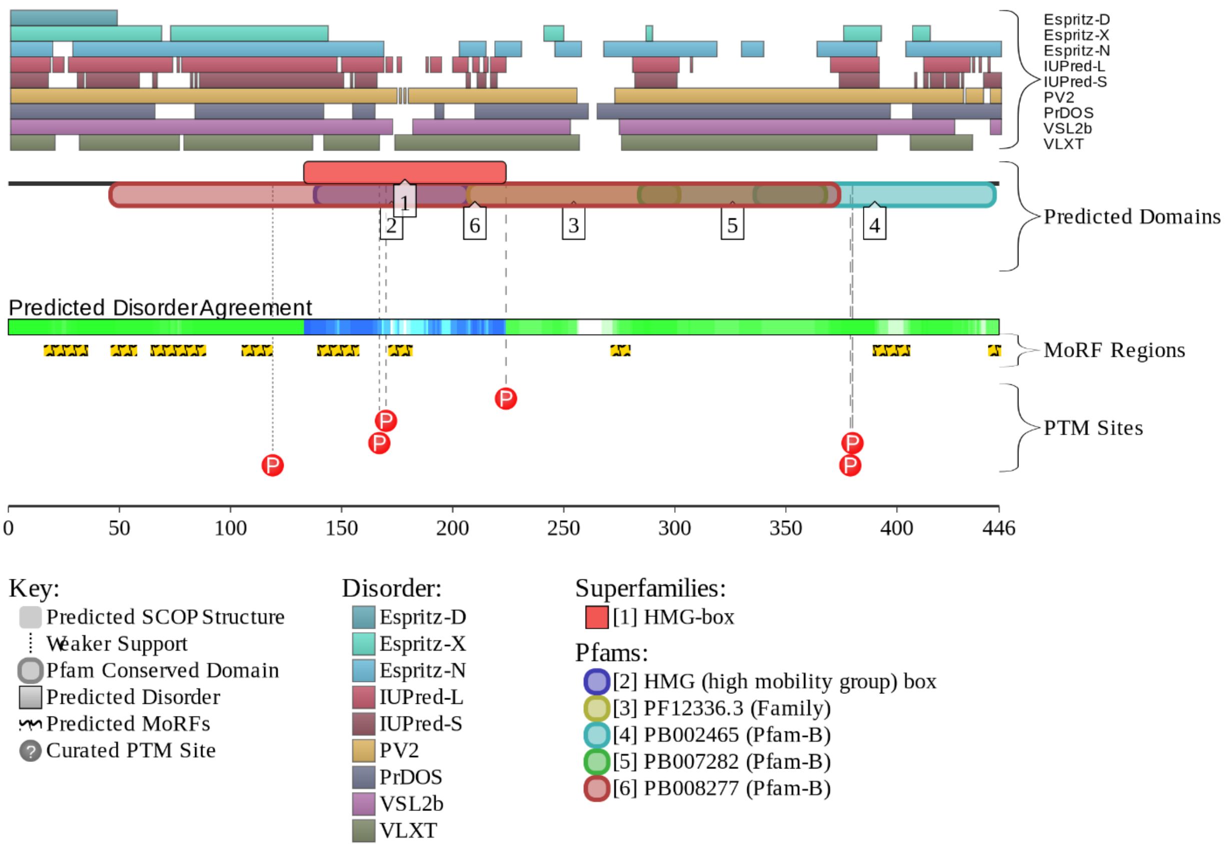

Figure S1F. D^2P^2 output for transcription factor SOX3 (Sex-determining region Y-box3, UniProt ID: P41225)

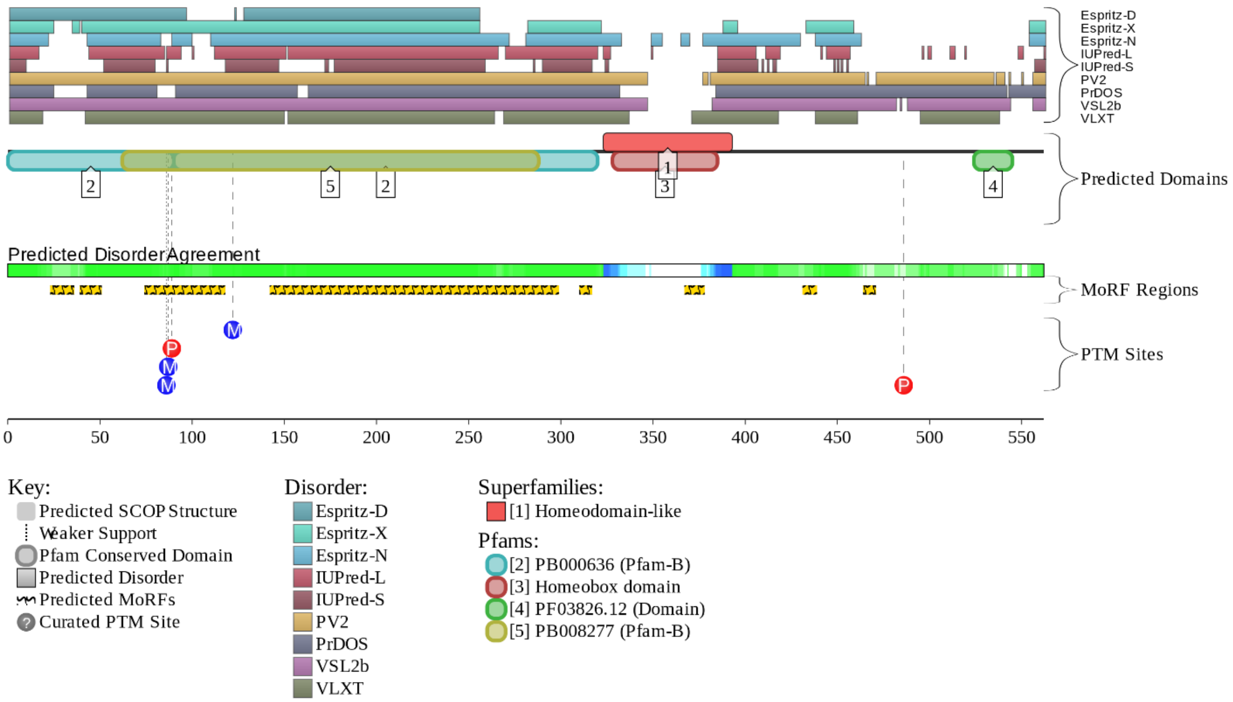

Figure S1G. D^{2}P^2 output for homeobox protein ARX (Aristaless-related homeobox, UniProt ID: Q96QS3)

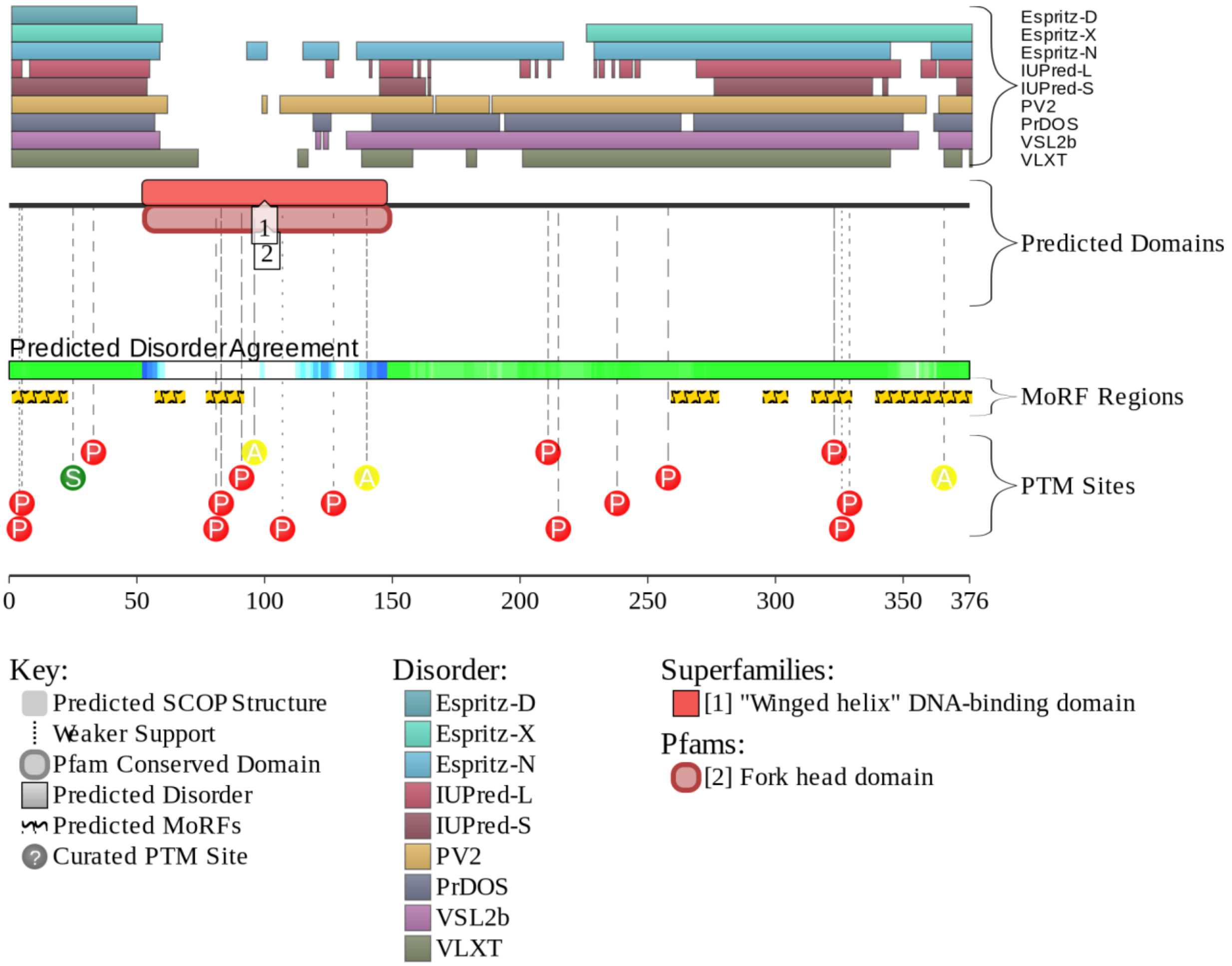

Figure S1H. D^2P^2 output for human forkhead box protein L2 (FOXL2, UniProt ID: P58012)

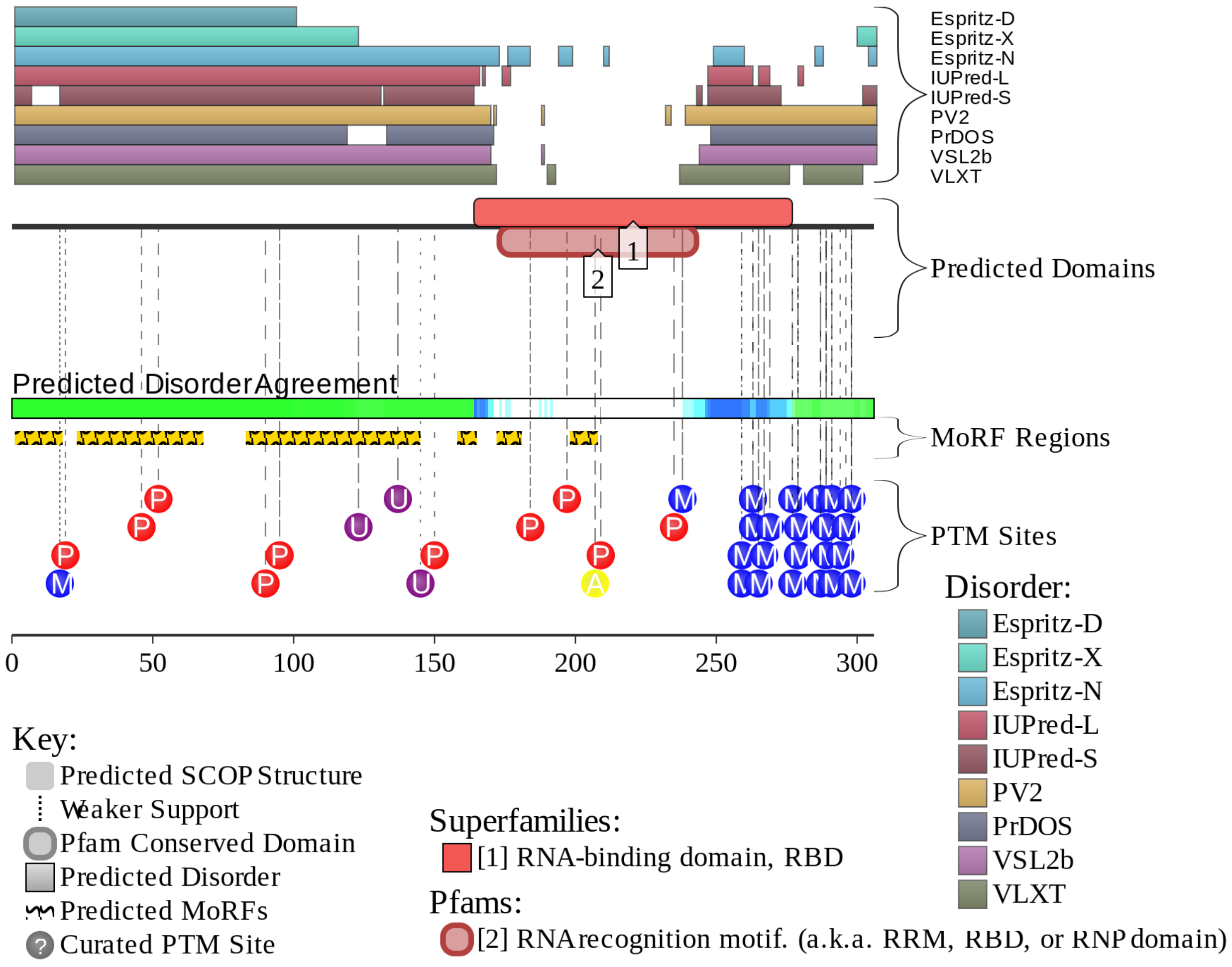

Figure S1I. D^2P^2 output for polyadenylate-binding protein 2/polyadenine-binding protein nuclear-1 (PABP2/PABPN1, UniProt ID: Q86U42)

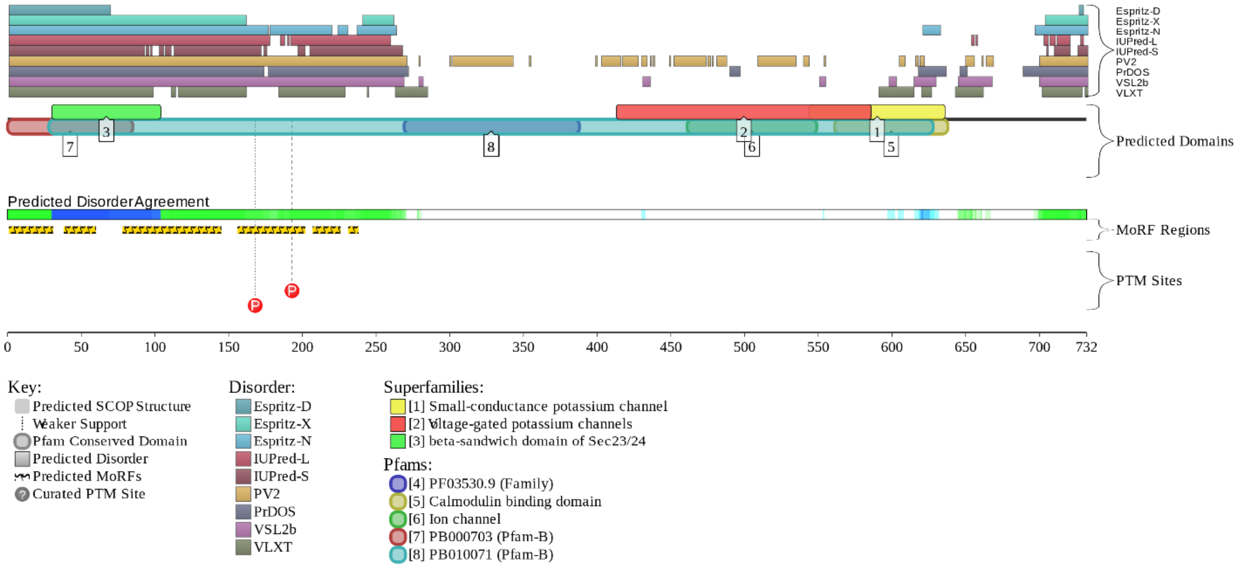

Figure S1J. D^{2}P^2 output for human small conductance calcium-activated potassium channel protein 3 (SK3, UniProt ID: Q9UGI6) is not available. Presented is the D^{2}P^2 output for mouse homologue (UniProt ID: P58391).

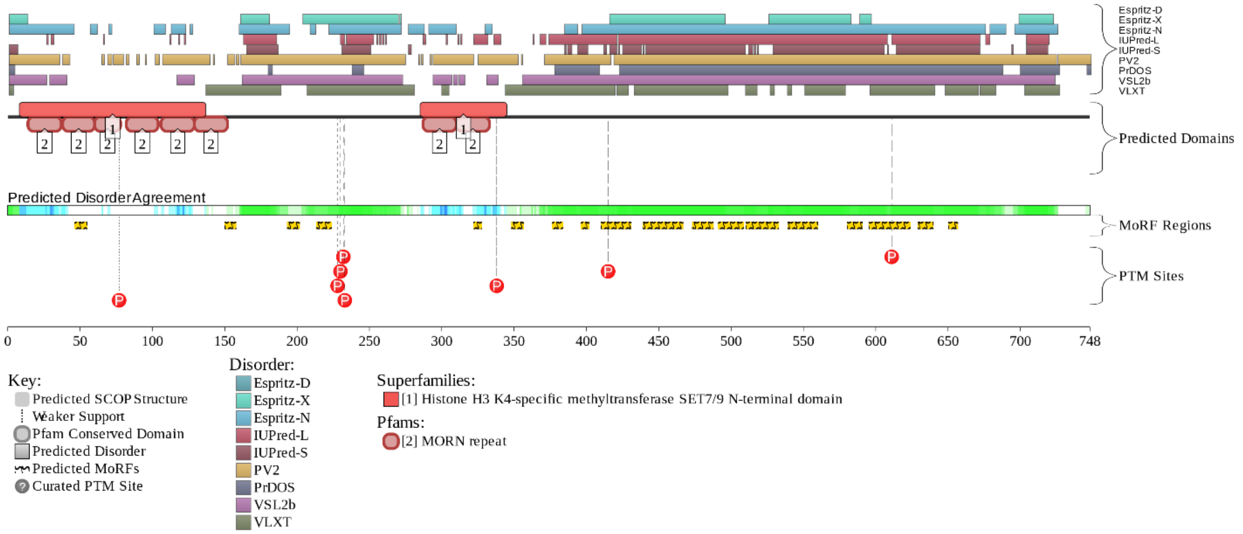

Figure S1K. D^2P^2 output for human junctophilin-3 (JP-3, UniProt ID: Q8WXH2)

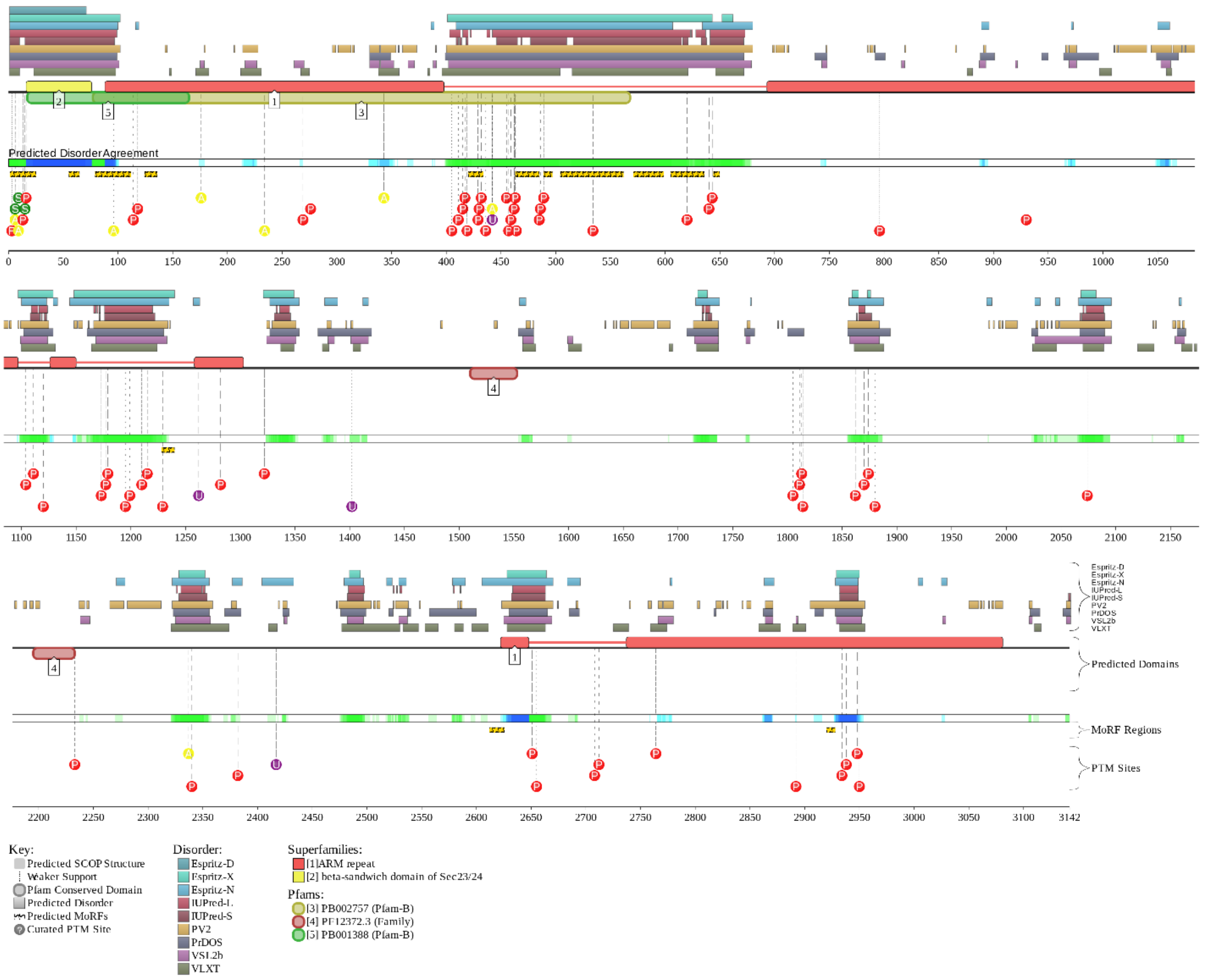

Figure S1L. D^2P^2 output for human huntingtin (UniProt ID: P42858)

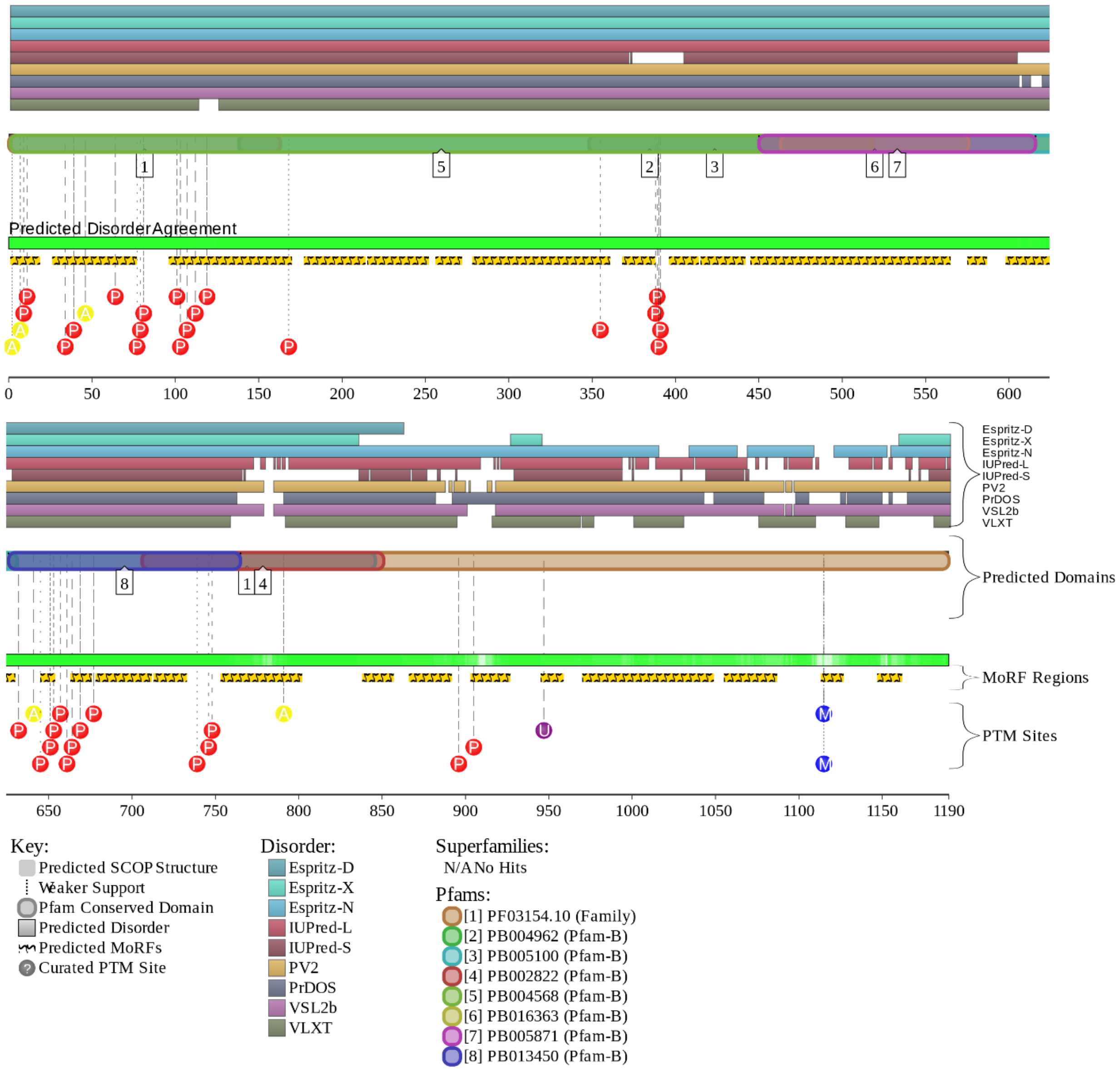

Figure S1M. D^{2}P^2 output for atrophin-1 (UniProt ID: P54259)

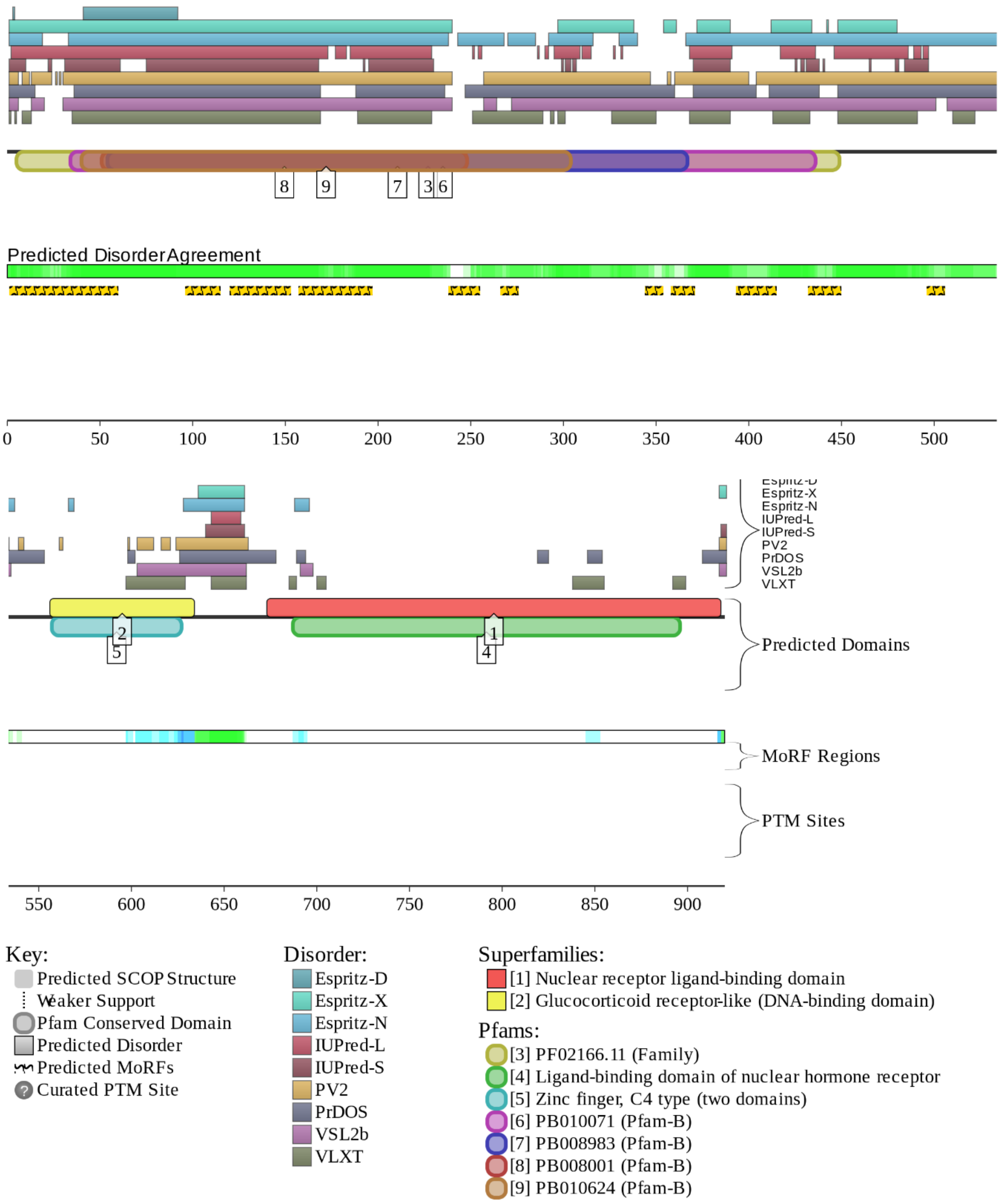

Figure S1N. D^{2}P^2 output for human androgen receptor (AR, UniProt ID: P10275)

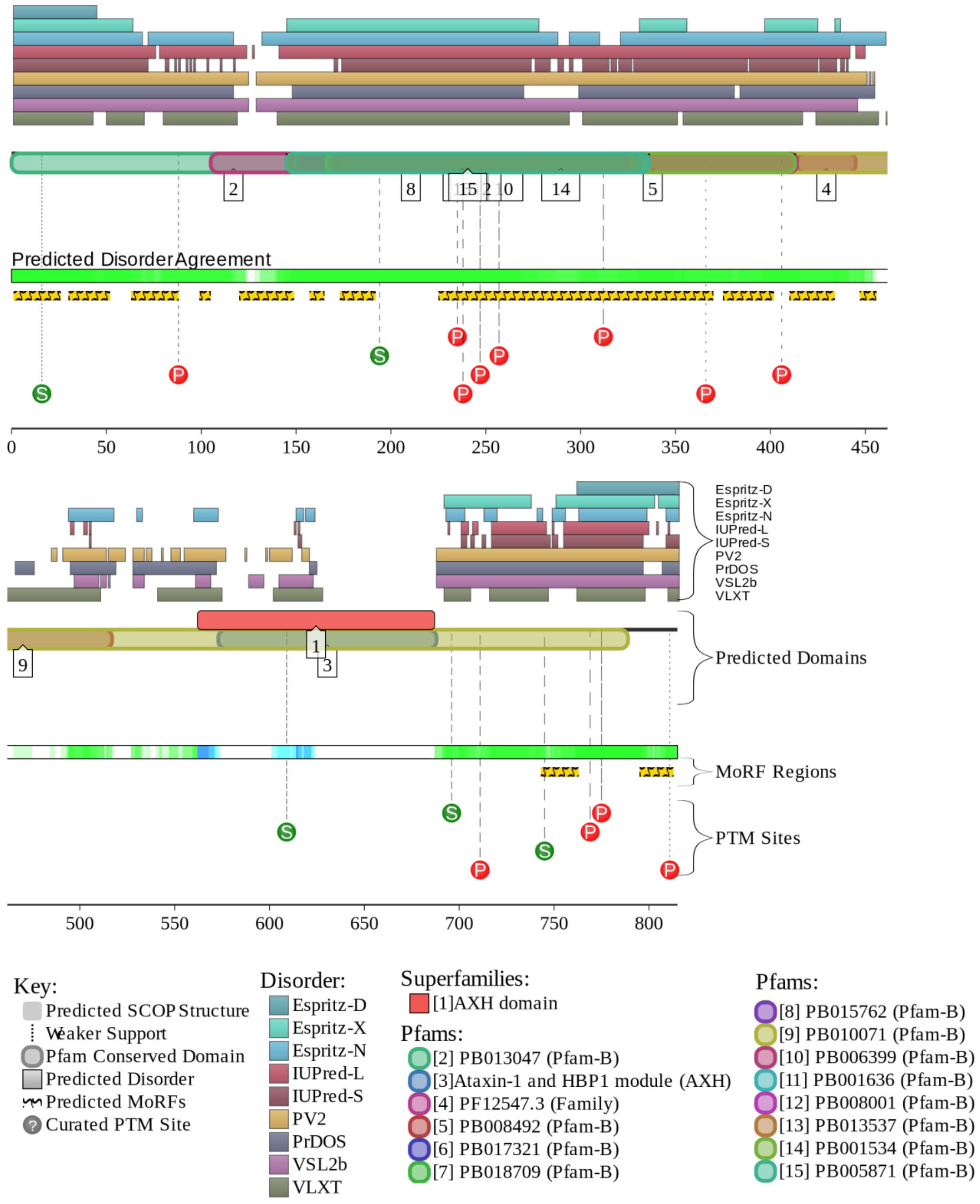

Figure S1O. D^{2}P^2 output for ataxin-1 (UniProt ID: P54253)

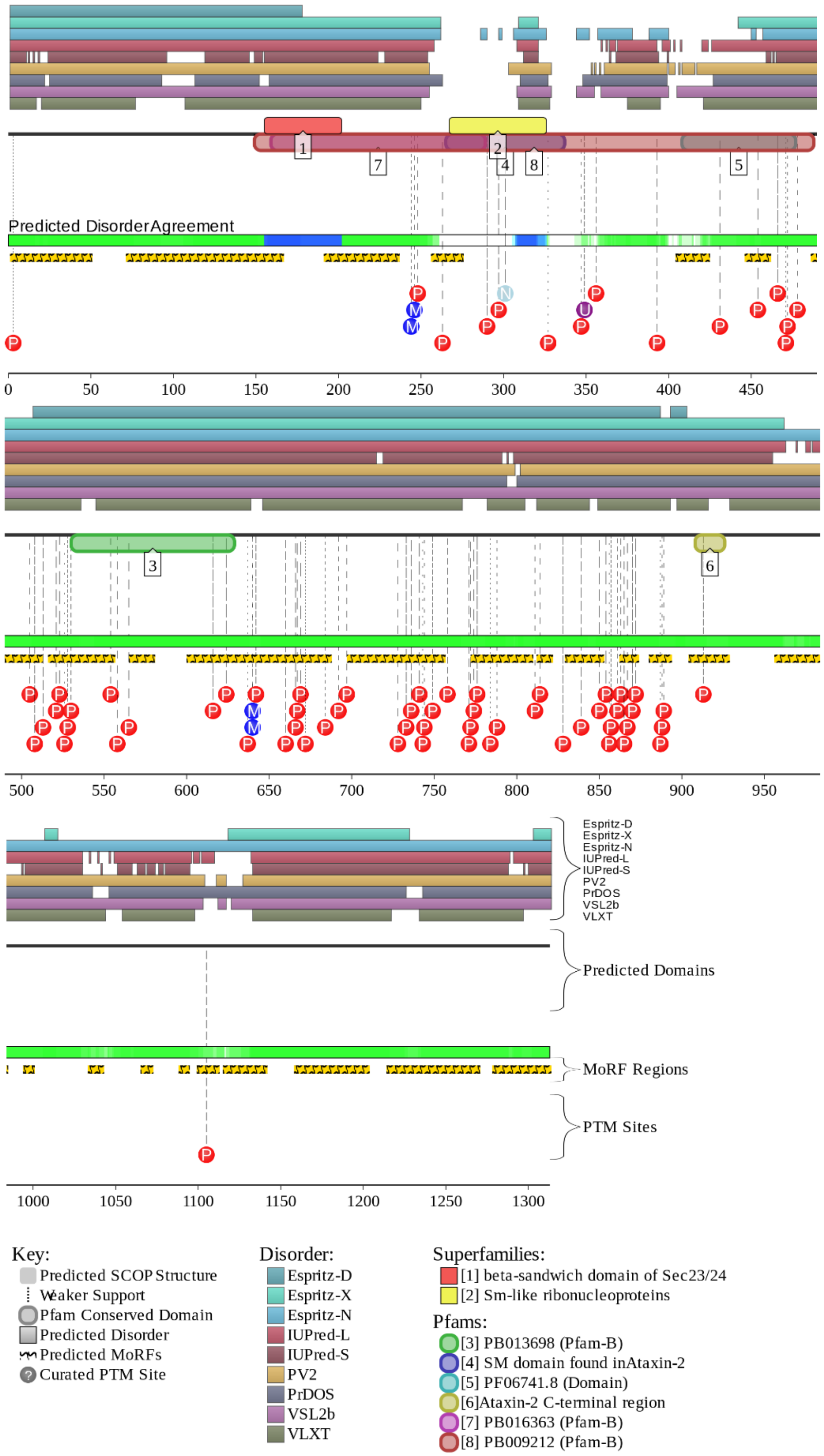

Figure S1P. D^{2}P^2 output for human ataxin-2 (UniProt ID: Q99700)

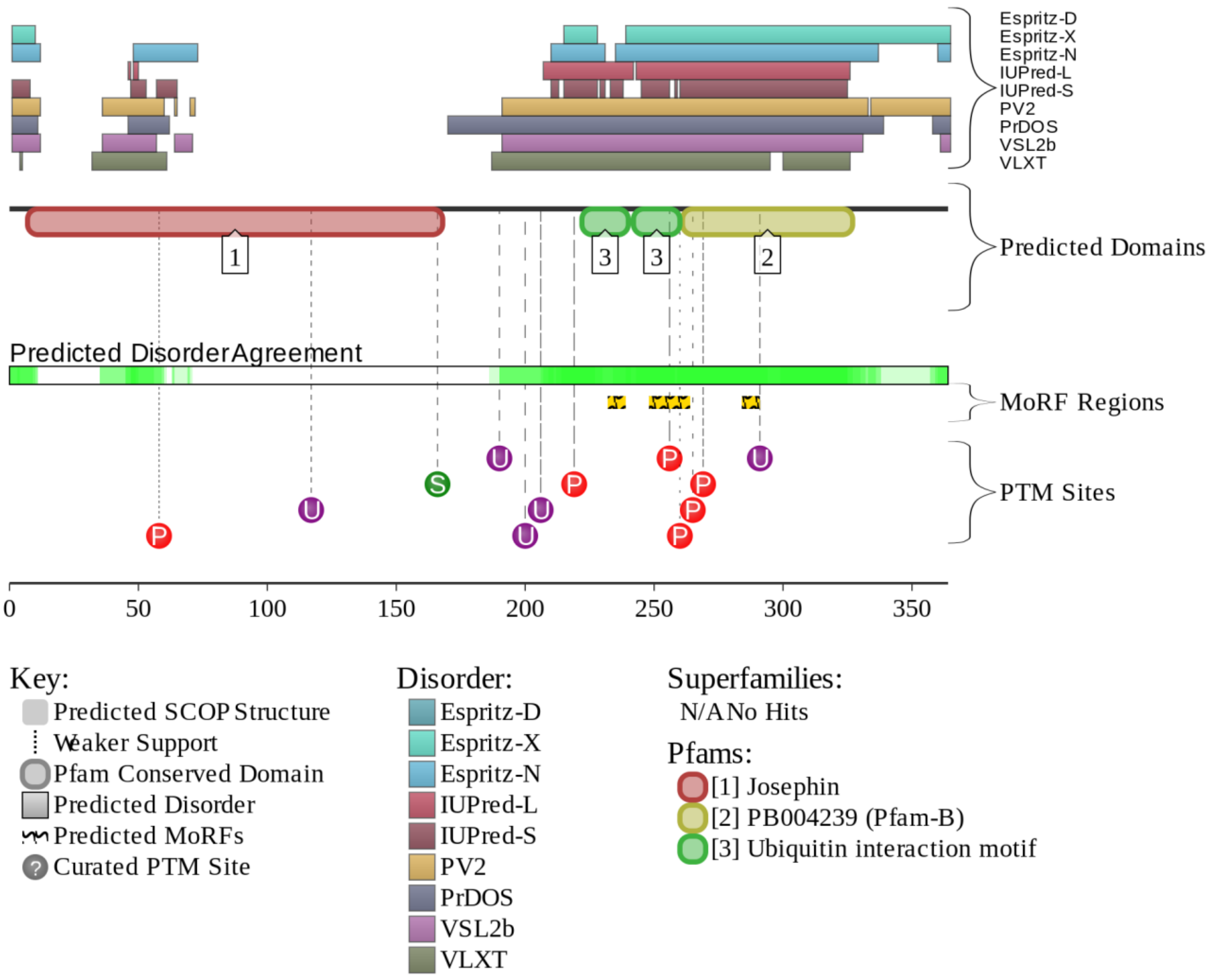

Figure S1Q. D^{2}P^2 output for human ataxin-3 (UniProt ID: P54252)

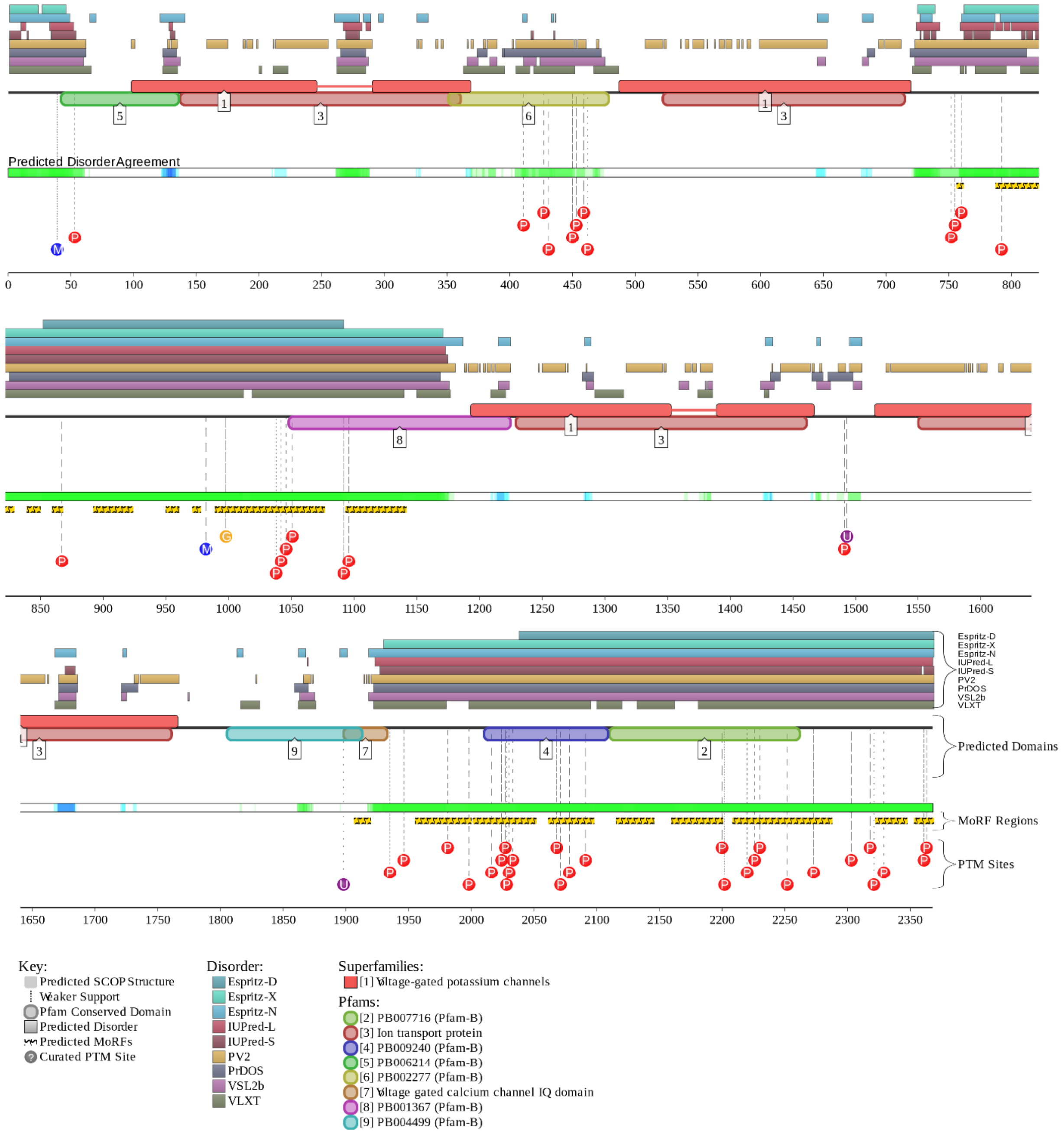

Figure S1R. D^2P^2 output for voltage-dependent P/Q-type calcium channel subunit α1A (CACNA1A, UniProt ID: O00555) is not available. Presented here is the D^2P^2 output for mouse CACNA1A (UniProt ID: P97445).

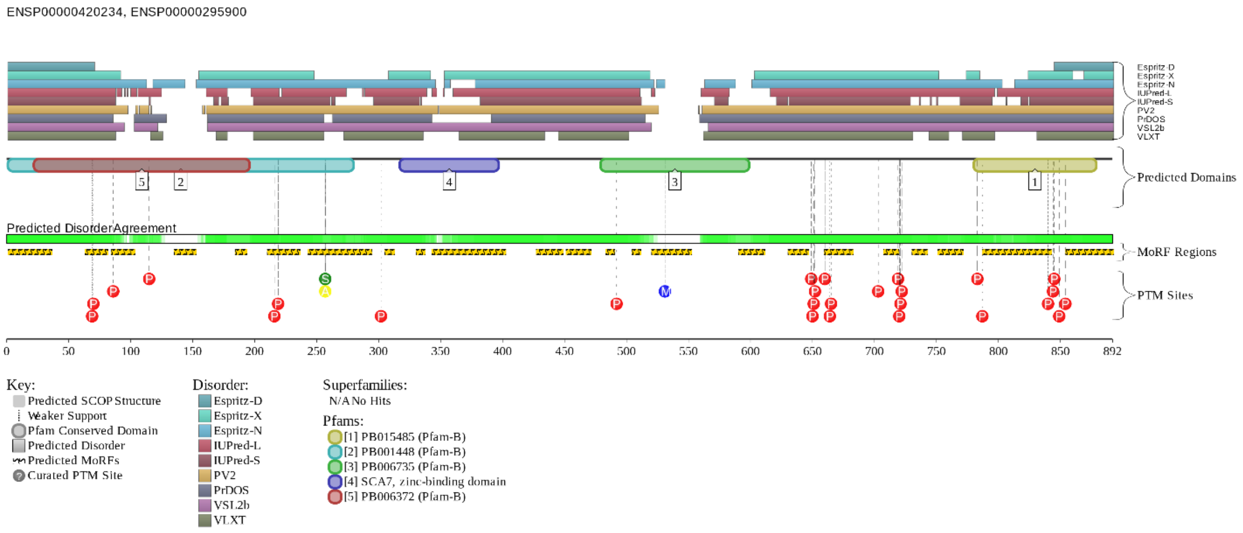

Figure S1S. D^{2}P^2 output for ataxin-7 (UniProt ID: O15265)

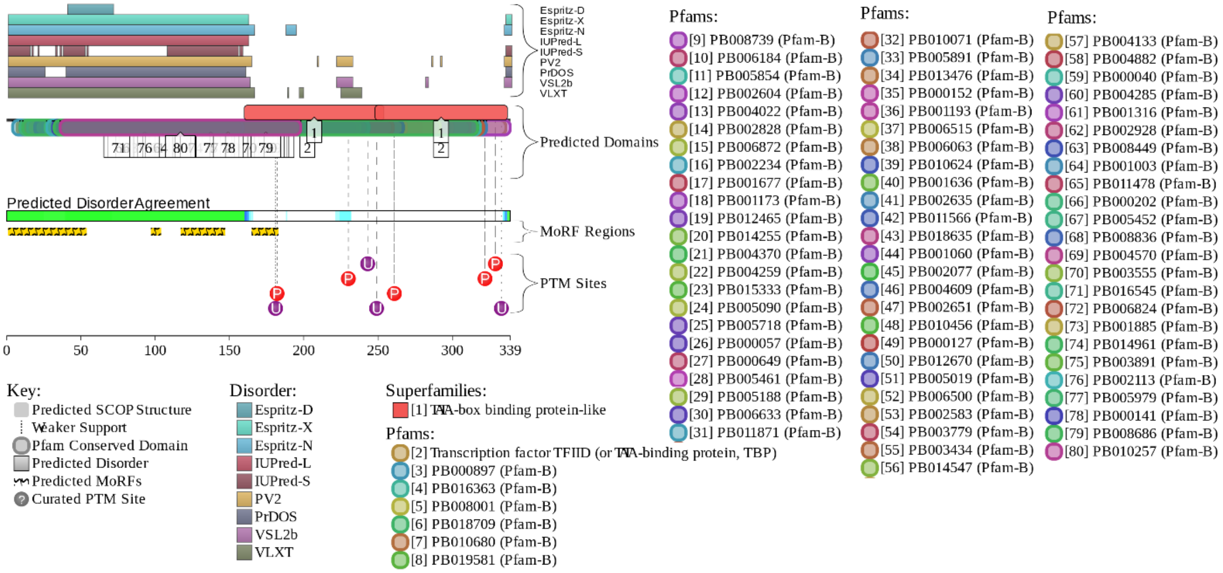

Figure S1T. D^{2}P^2 output for TATA-box-binding protein (TBP, UniProt ID: P20226)

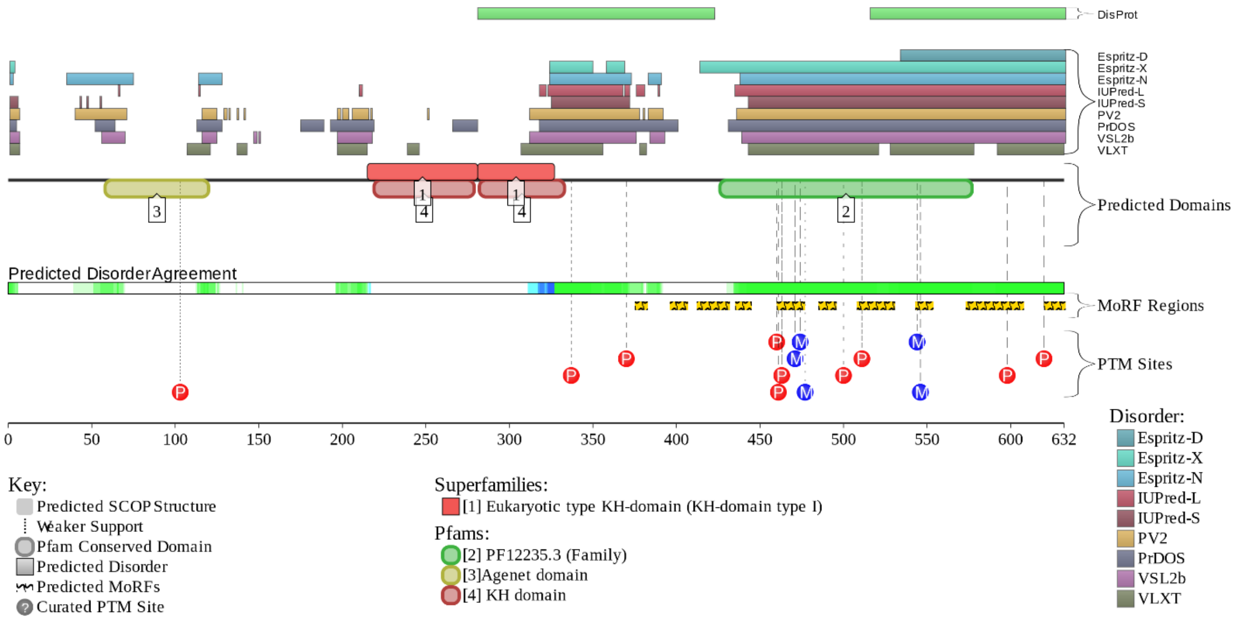

Figure S1U. D^{2}P^2 output for synaptic functional regulator FMR1 (UniProt ID: Q06787)

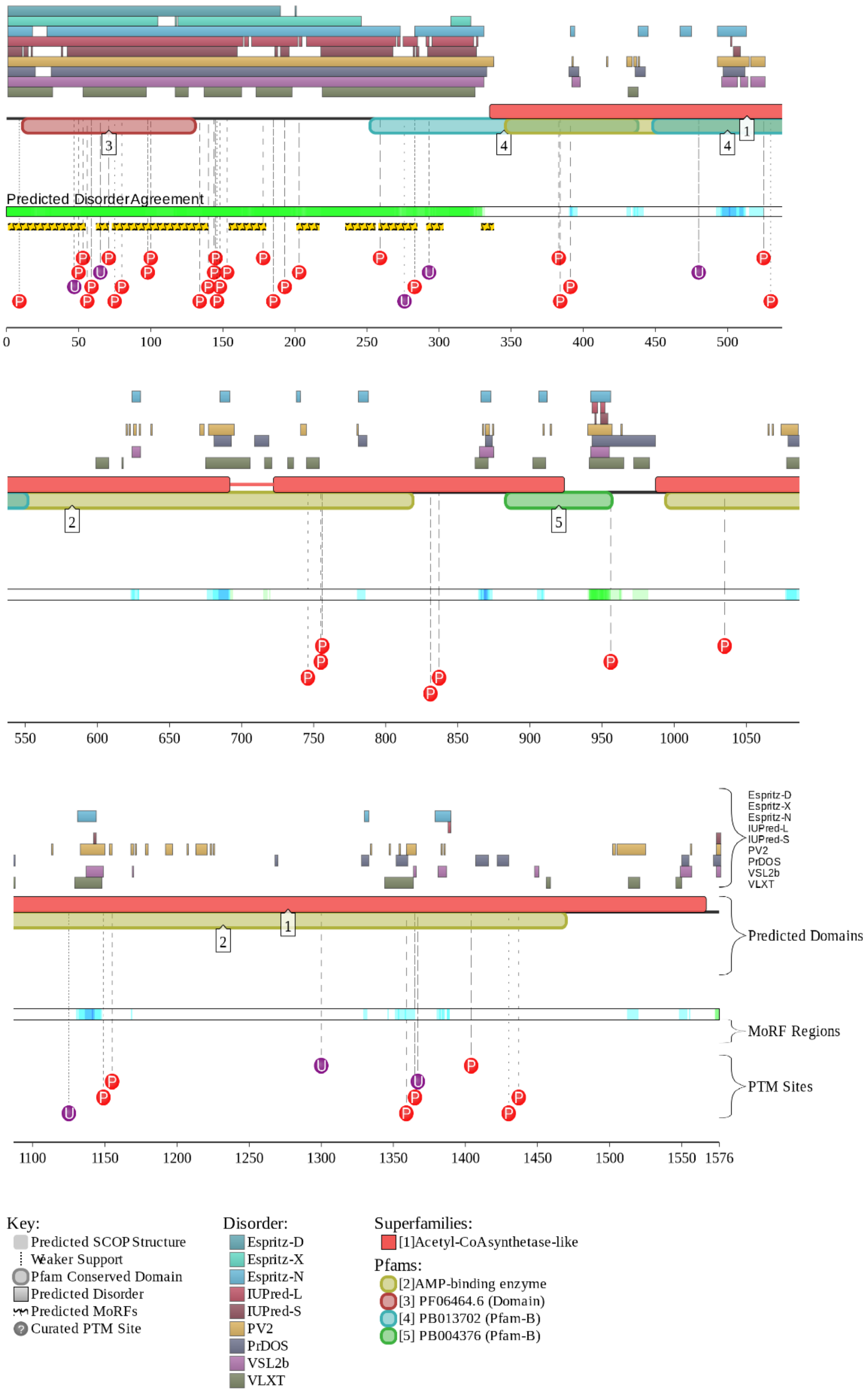

Figure S1V. D^{2}P^2 output for disco-interacting protein 2 homolog B (UniProt ID: Q9P265)

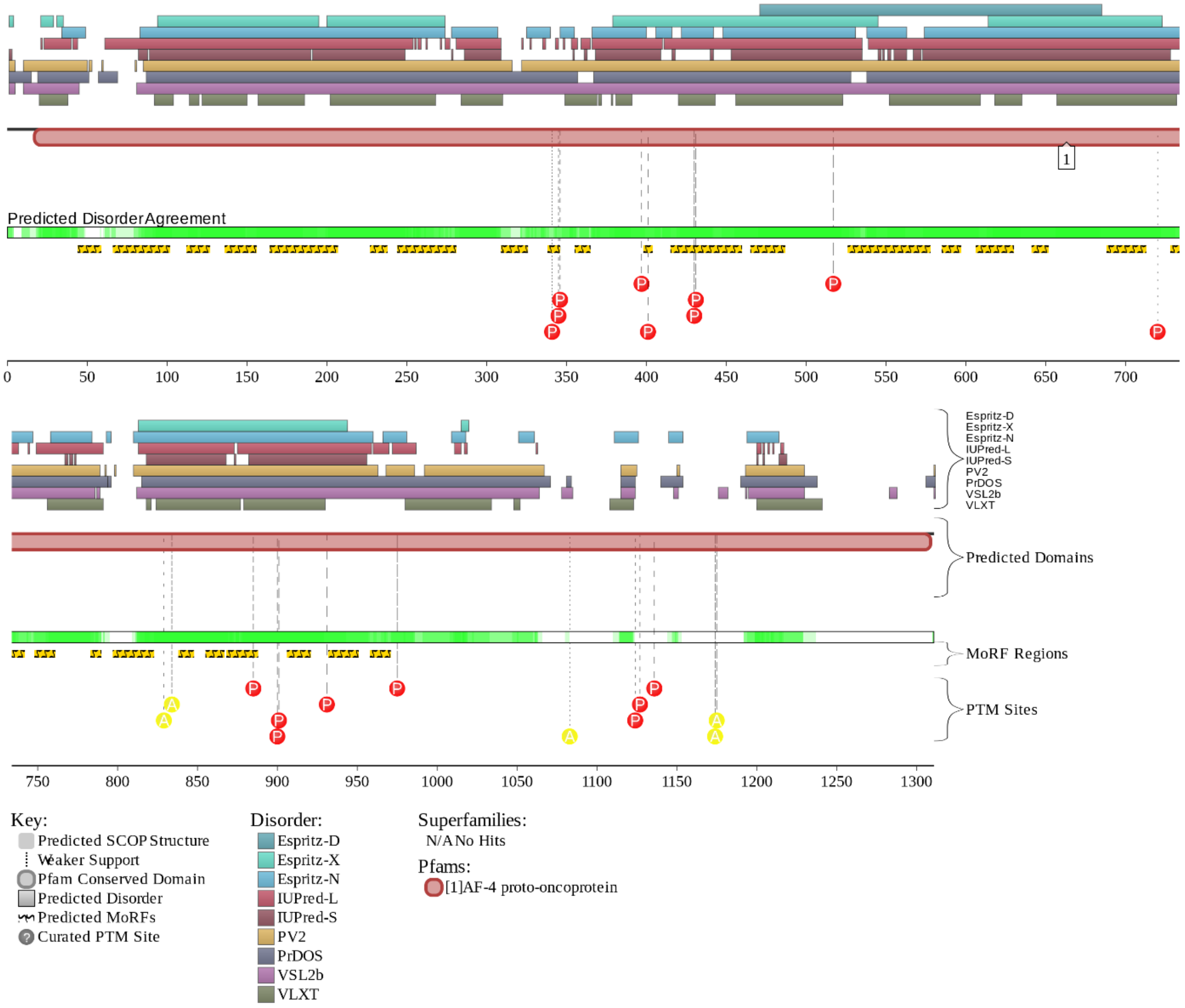

Figure S1W. D^{2}P^2 output for AF4/FMR2 family member 2 (UniProt ID: P51816)

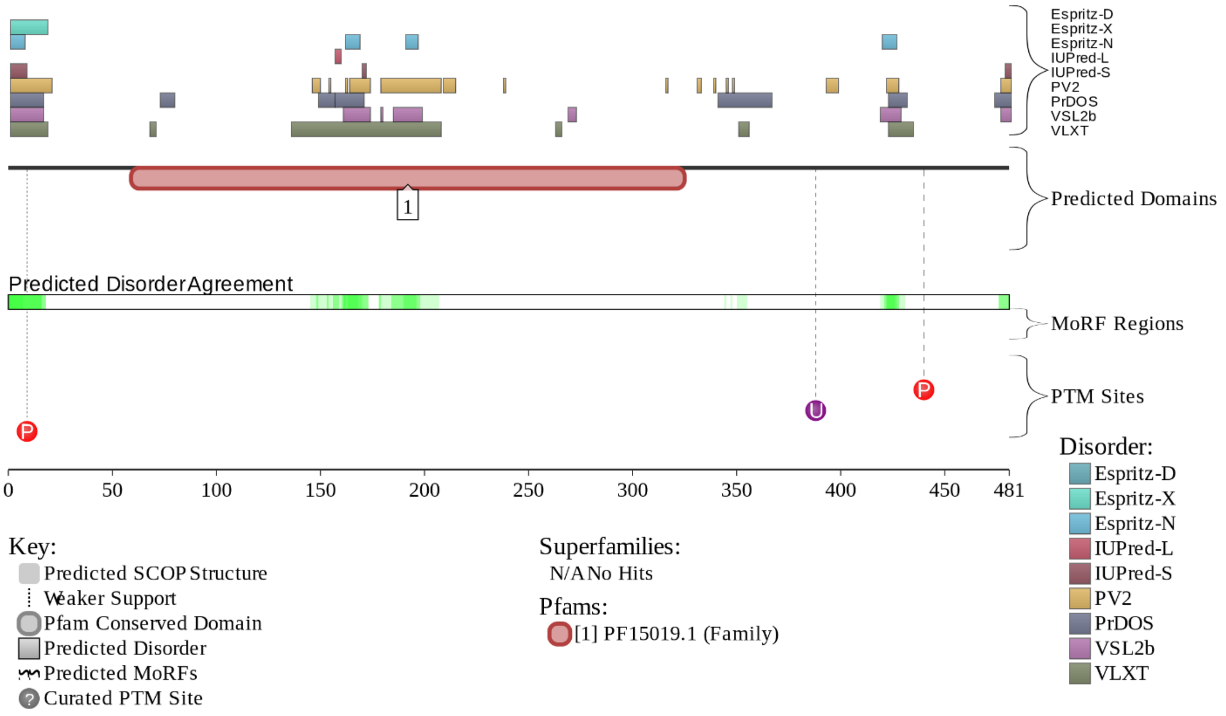

Figure S1X. D^2P^2 output for C9orf72 (UniProt ID: Q96LT7)

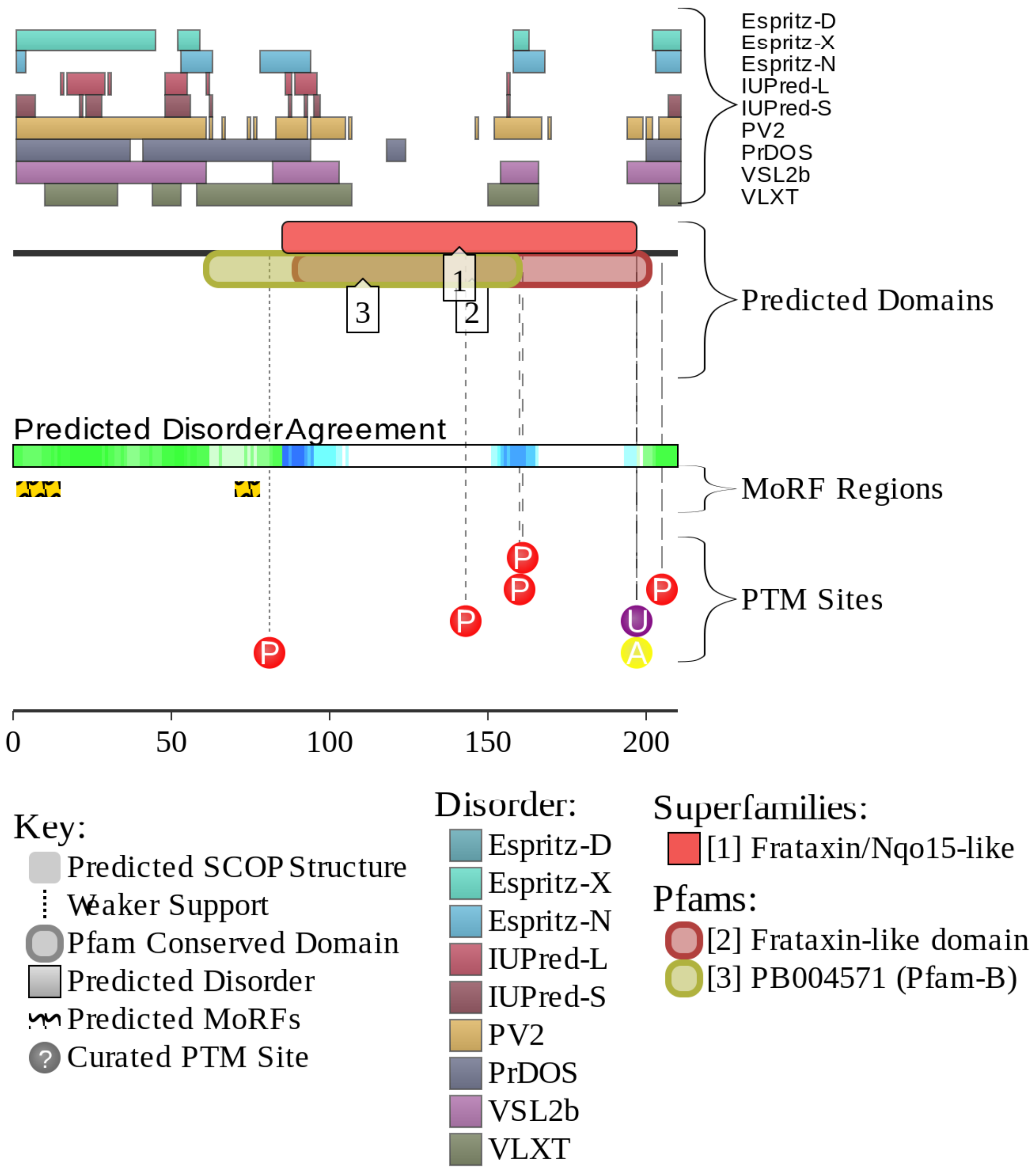

Figure S1Y. D^{2}P^2 output for frataxin (UniProt ID: Q16595)

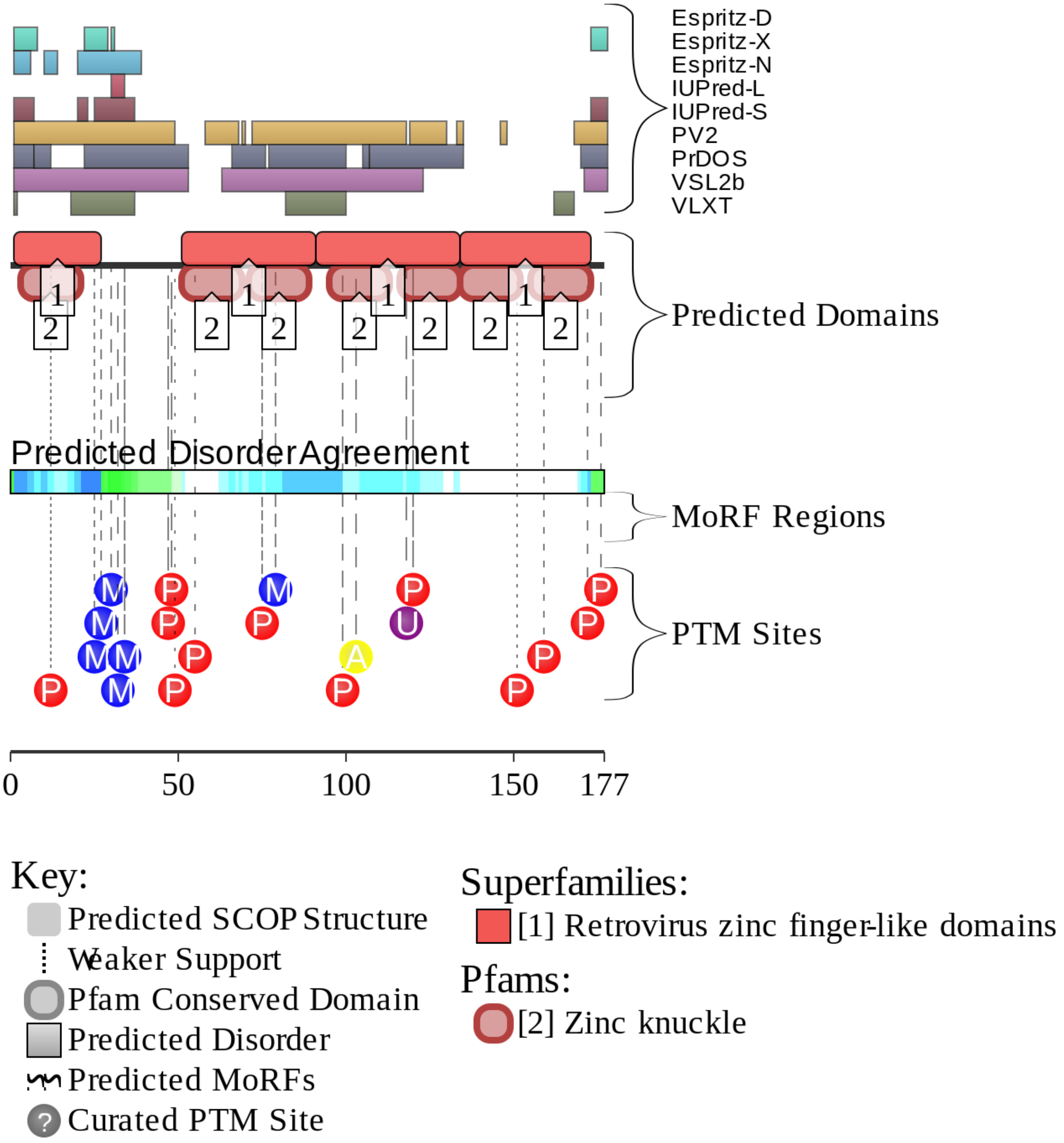

Figure S1Z. D^2P^2 output for cellular nucleic acid-binding protein (CNBP, UniProt ID: P62633)

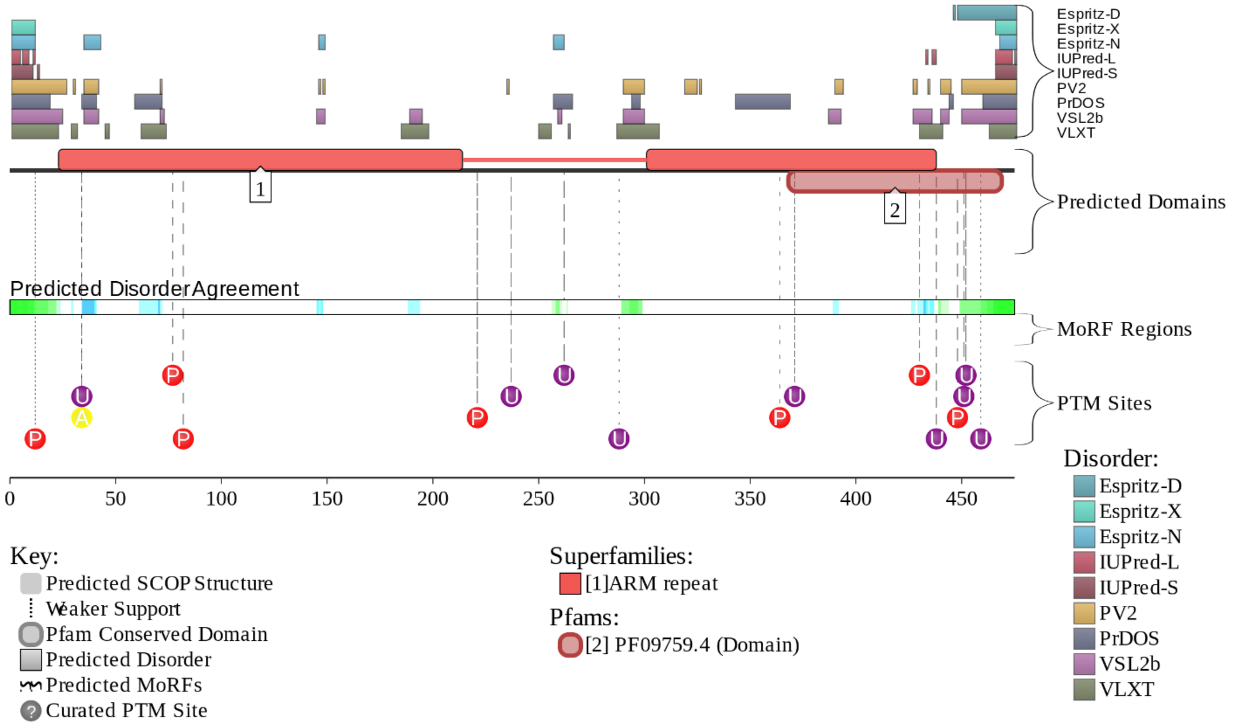

Figure S1a. D^2P^2 output for ataxin-10 (UniProt ID: Q9UBB4)

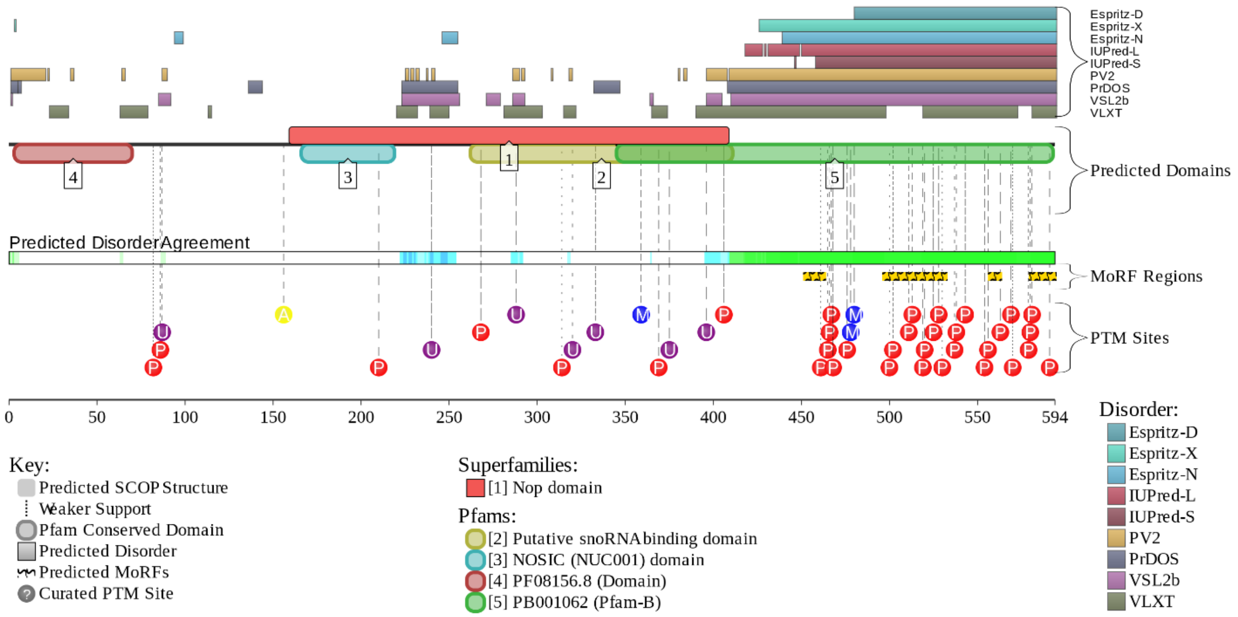

Figure S1b. D^2P^2 output for nucleolar protein 56 (UniProt ID: O00567)

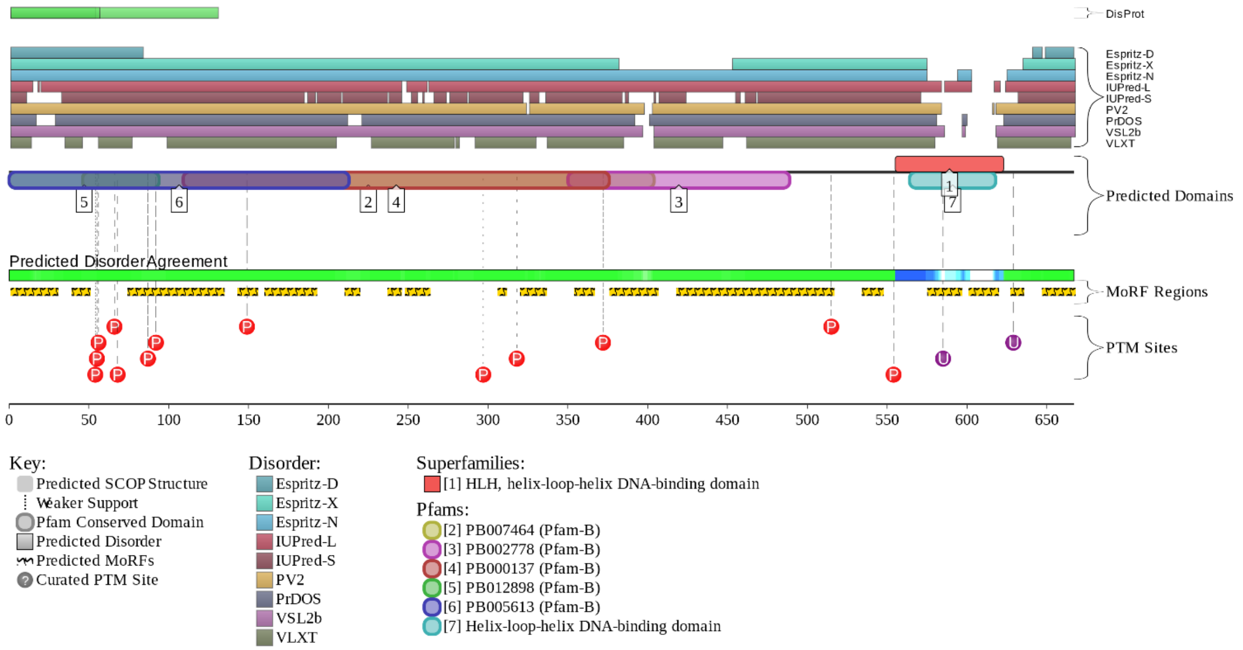

Figure S1c. D^{2}P^2 output for transcription factor 4 (UniProt ID: P15884)

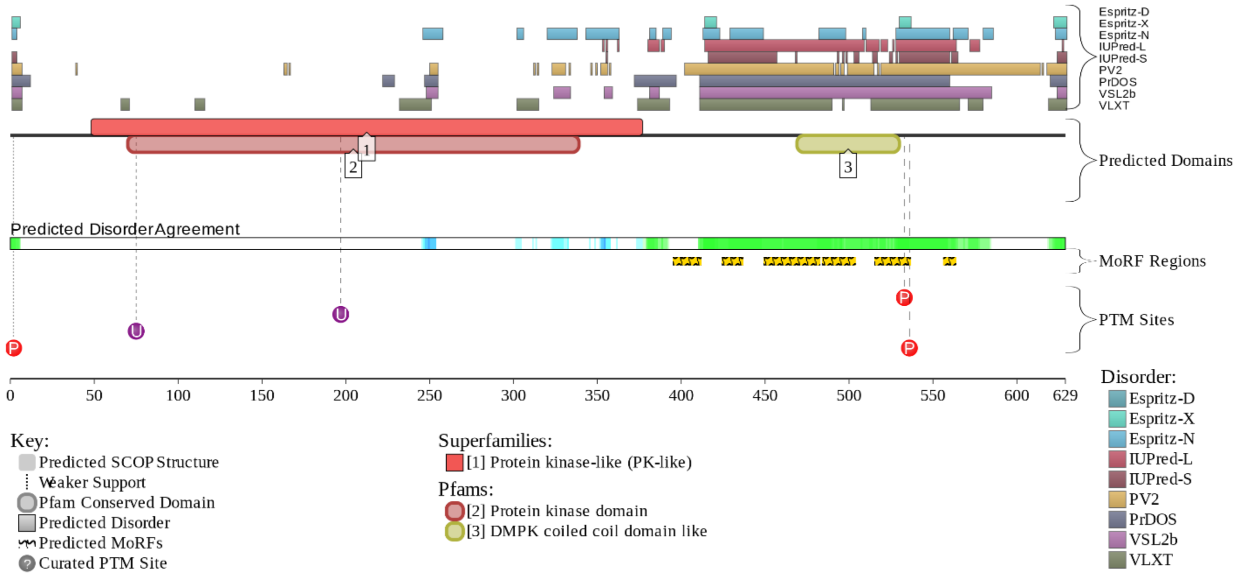

Figure S1d. D^{2}P^2 output for myotonin-protein kinase (UniProt ID: Q09013)

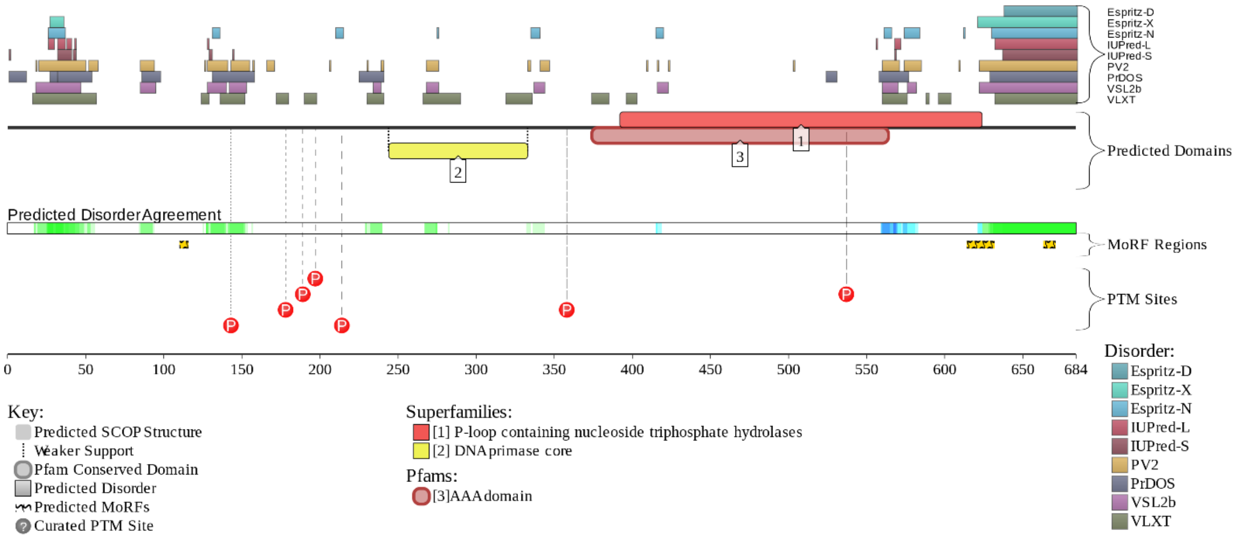

Figure S1e. D^2P^2 output for mitochondrial twinkle protein (UniProt ID: Q96RR1)

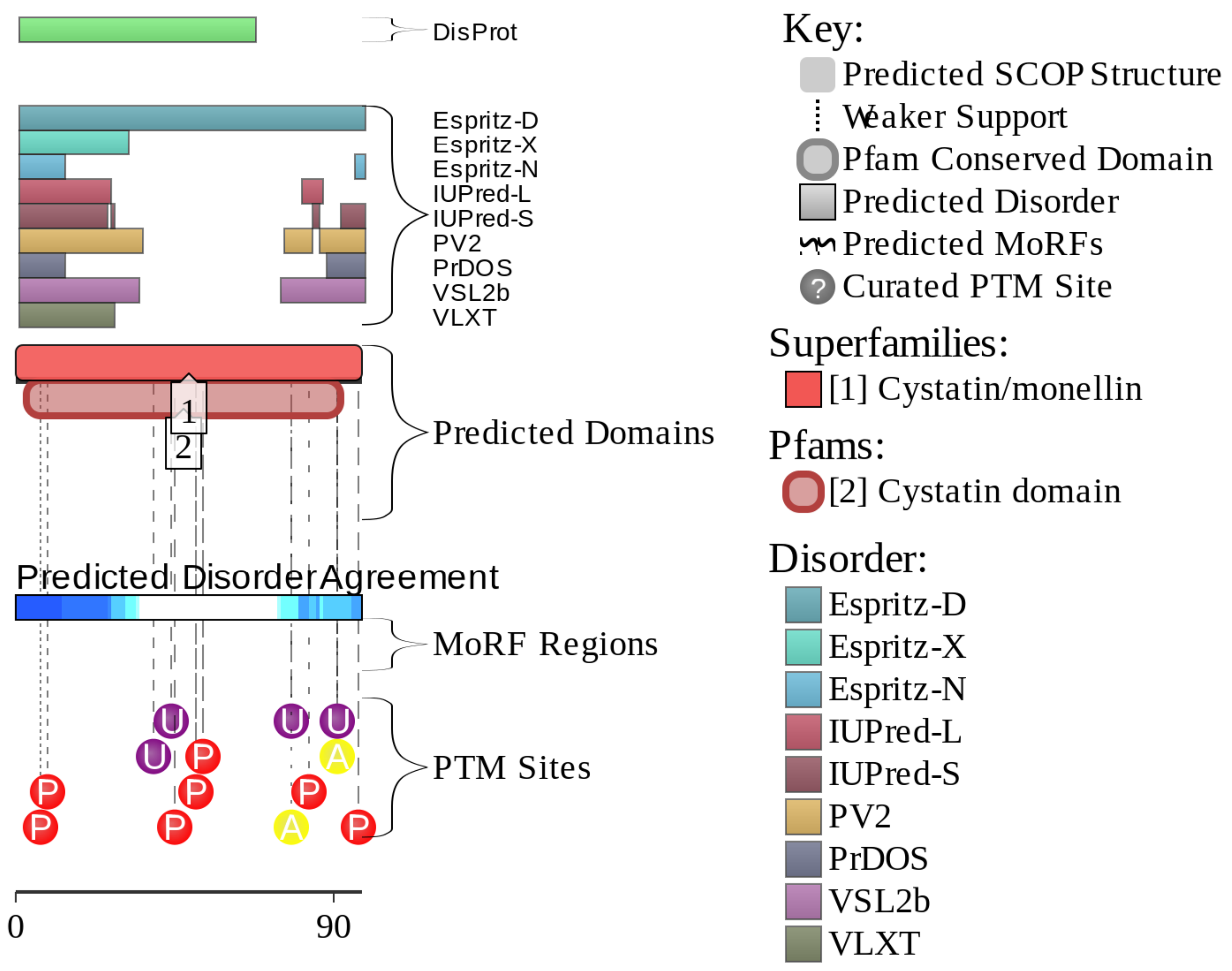

Figure S1f. D^{2}P^2 output for cystatin-B (UniProt ID: P04080)

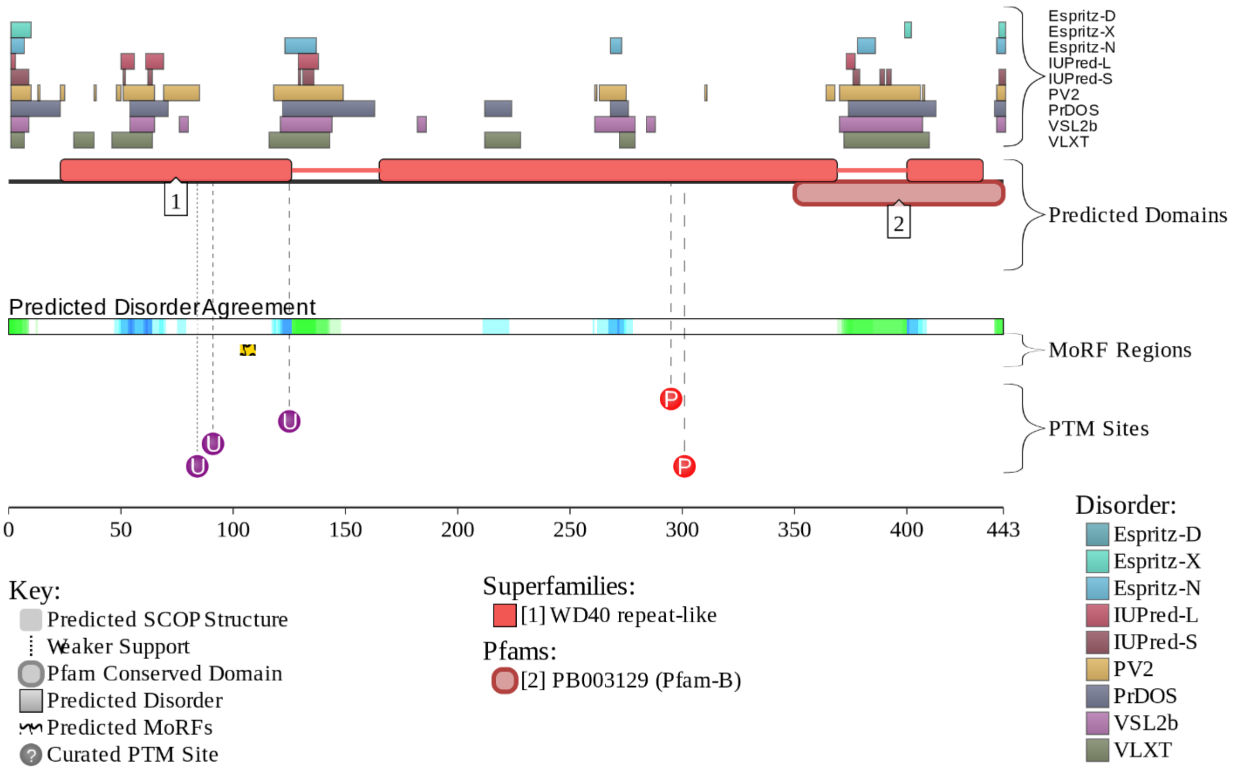

Figure S1g. Serine/threonine-protein phosphatase 2A 55 kDa regulatory subunit B β isoform (PPP2R2B, UniProt ID: Q00005).
